# Square-pyramidal copper(ii)-Schiff base complexes with methoxy/ethoxy-phenolate and chloro-benzophenone substituents

**DOI:** 10.1039/d6ra04681a

**Published:** 2026-07-23

**Authors:** Imdadul Haque, Galib Abdullah, Kashfia Azad Tuba, Yizhou Wang, Yanping Ma, Wen-Hua Sun, Christoph Janiak, Mohammed Enamullah

**Affiliations:** a Department of Chemistry, Jahangirnagar University Dhaka-1342 Bangladesh enamullah@juniv.edu; b Institute of Chemistry, Chinese Academy of Sciences Haidian Beijing 100190 P. R. China; c Institut für Anorganische Chemie und Strukturchemie, Heinrich-Heine-Universität D 40204 Düsseldorf Germany; d Vice Chancellor, Hajee Mohammad Danesh Science & Technology University (HSTU) Dinajpur Bangladesh

## Abstract

The Schiff bases, (*E*)-2-(((2-benzoylphenyl)imino)methyl)-6-methoxyphenol (HL1), (*E*)-2-(((2-benzoylphenyl)imino)methyl)-6-ethoxyphenol (HL2) and (*E*)-2-(((2-benzoyl-4-chlorophenyl)imino)methyl)-6-ethoxyphenol (HL3) react with copper(ii) acetate to give [Cu(L1)_2_] (1), [Cu(L2)_2_] (2) and [Cu(L3)_2_] (3) complexes, respectively. Structural analyses revealed that the Schiff bases adopt the conventional enol-imine O–H⋯N(imine) form stabilized by an intramolecular hydrogen bond. The structures of the complexes show coordination of two *N*,*O*-chelate ligands to the Cu(ii) atom with *trans-N*,*N*′ and -*O*,*O*′ configuration and a long fifth elongated coordination Cu⋯O=C(benzoyl) in an overall square-pyramidal geometry (*τ*_5_ = 0.005–0.031). Each structure contains a single crystallographically independent molecule within the asymmetric unit. The experimental and simulated PXRD profiles are in good agreement, confirming the phase purity and integrity of bulk microcrystalline samples consistent with those of the single crystals. DSC analyses suggest a simple melting of solid crystals (*i.e.*, m.p.) with an irreversible phase change. Cyclic voltammetry (CV) measurements show two successive one-electron charge-transfer redox processes of the complexes. DPPH (2,2-diphenyl-1-picrylhydrazyl) radical-scavenging assays demonstrate that the free Schiff bases exhibit higher antioxidant activity than corresponding copper(ii) complexes. Finally, molecular modeling by DFT/TD-DFT and MO calculations reproduce the experimental results of the absorption spectra and molecular structures.

## Abstract 2

Square-pyramidal copper(ii) complexes were obtained from Schiff bases containing the methoxy/ethoxy-phenolate and chloro-benzophenone substituents.

## Graphical abstract

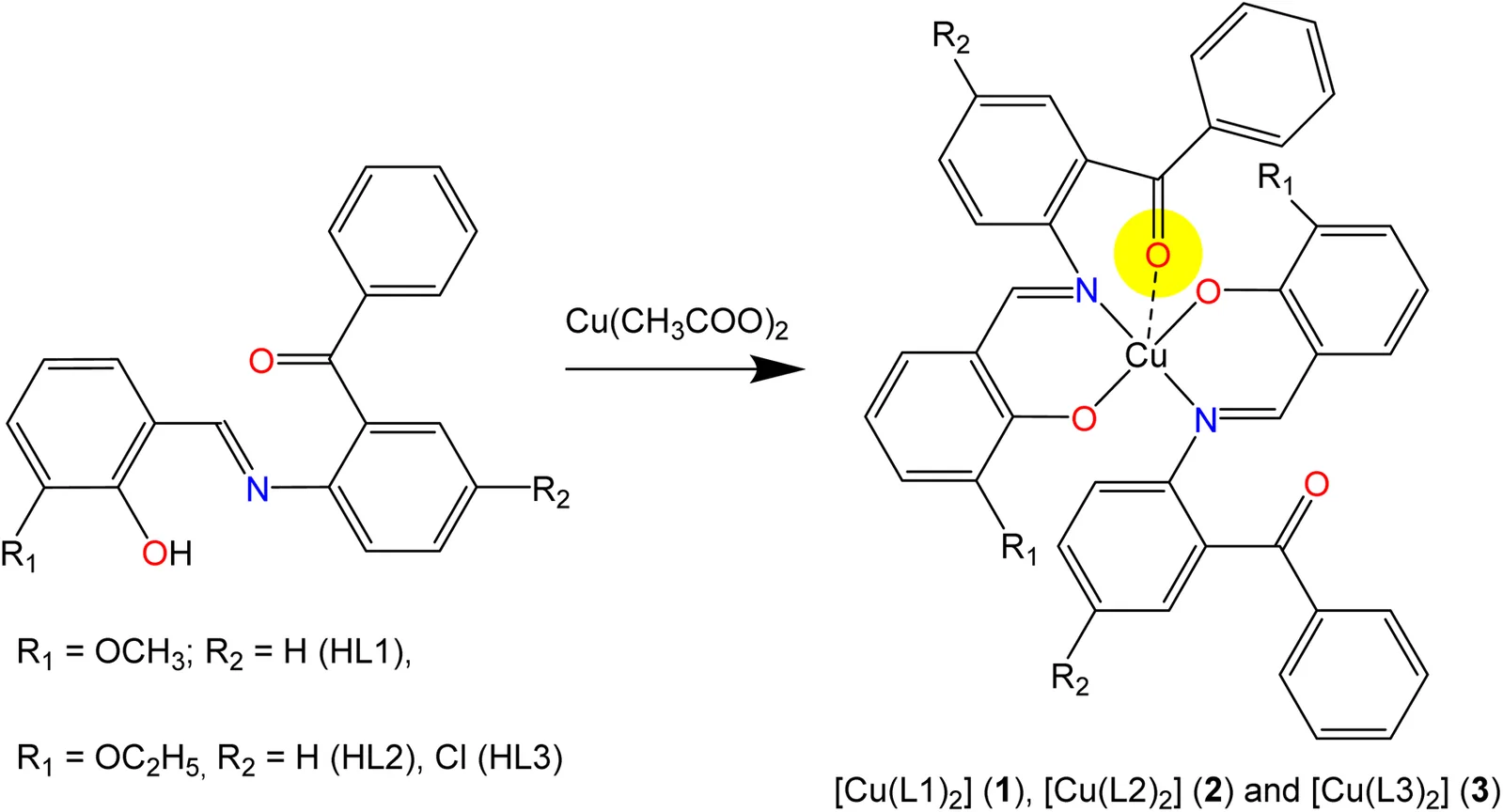

## Introduction

Schiff base ligands obtained from 2-hydroxy aromatic aldehydes and primary amines constitute one of the most widely explored ligand systems in coordination chemistry, owing to their facile synthesis, structural versatility, and strong chelating ability *via N*,*O*-donor sets.^1–3^ Salicylaldehyde/naphthaldehyde based Schiff bases frequently exhibit keto–enol tautomerism and provide stable intramolecular O–H⋯N hydrogen-bonds, playing a vital role in stabilizing their structural conformation and dictating coordination modes toward transition-metal ions.^3–5^ These features make such ligands highly suitable for constructing metal complexes with well-defined geometries and tunable physicochemical properties.^6^ Among transition metals, copper(ii) occupies a central position in Schiff base coordination chemistry because of its accessible redox states, flexible coordination environments, and relevance to biological and catalytic systems.^2–5,7,8^ Copper(ii) complexes with *N*,*O*-chelating ligands typically adopt square-planar or square-pyramidal geometries depending on steric and electronic factors imposed by the ligand framework.^2–5,7–10^ Subtle modifications of ligand substitutes can therefore significantly influence coordination mode, molecular and electronic structure, and intermolecular interactions, leading to diverse structural architectures and functional behaviors.^7–12^ In fact, analogous copper(ii) Schiff base complexes have demonstrated that the presence of substituents (methoxy/ethoxy) on the salicylal/naphthalal ring not only governs structures, ranging from square-planar to distorted square-planar or square-pyramidal geometries with different compositions of molecule in the unit cell or asymmetric unit, but also provides polymorphic and mesomorphic (liquid-crystal) behaviors.^2,4,7,9^ In addition to their structural diversity, copper(ii)-Schiff base complexes have attracted sustained interest owing to their wide-ranging applications in catalysis, materials chemistry, and bioinorganic science.^1,2,4,9,11,13^ Their redox-nature enables participation in electron-transfer processes, while conjugated ligand frameworks facilitate optical and electronic transitions relevant to photophysical and electronic applications.^9,11,14,15^ Furthermore, the presence of hydrogen-bonding sites and aromatic surfaces often promotes supramolecular aggregation *via* non-covalent interactions (*i.e.*, hydrogen-bonding and π–π stacking), which can extend into higher-dimensional architectures.^1,2,9,16^

Single-crystal X-ray diffraction studies suggest that electronic variations not only affect coordination geometry but also influence supramolecular assembly through non-covalent interactions.^17–19^ These interactions further contribute to extended supramolecular network formation, which is particularly relevant for applications in molecular electronics, luminescence and host–guest chemistry.^20–22^ For instance, halogen-substituted ligands tend to enhance stability through halogen-bonding, resulting in highly ordered frameworks.^23–26^ In catalytic applications, copper(ii)-Schiff base complexes demonstrate efficiency in oxidation reactions, asymmetric catalysis and polymerization processes. The electronic behaviors of Schiff bases influence redox potential of the metal center, thereby modulating catalytic activities.^27–30^ Studies suggest that electron-donating substituents enhance catalytic efficiency by stabilizing reactive intermediates, while electron-withdrawing groups facilitate oxidative addition and improve catalytic turnover.^31,32^ Beyond catalysis, metal-Schiff base complexes exhibit significant biological activities, including antimicrobial, anticancer and antioxidant properties.^2,4,9,11,33–35^ Substituent modifications on ligands further offer a strategy for optimizing biological activity by fine-tuning these properties.^36,37^ A comprehensive understanding of electronic effects of substituents on the salicylal/naphthalal unit in the Schiff base ligands on copper(ii) coordination profile is essential for rational ligand design.^38–40^ The incorporation of electron-withdrawing (halogen/nitro) and electron-donating (alkyl/methoxy/ethoxy) entities on the salicylal/naphthalal moiety was aimed at fine-tuning the electronic, steric, redox and coordination ability of Schiff bases towards metal ions.^41,42^

We report herein three newly synthesized Schiff bases with OCH_3_/OC_2_H_5_-phenolate and Cl-benzophenone substituents (HL1–HL3) and their copper(ii) complexes (1–3). Structure analysis reveals a square-pyramidal geometry for the complexes, while the free Schiff bases exist in the conventional O–H⋯N(imine) form. The Schiff bases and complexes were further analyzed using PXRD, DSC, CV, and DPPH assays. In addition, molecular modeling by DFT/TD-DFT and MO calculations was applied to rationalize molecular and electronic structure, particularly the electronic absorption spectra.

## Results and discussion

**Scheme 1:**
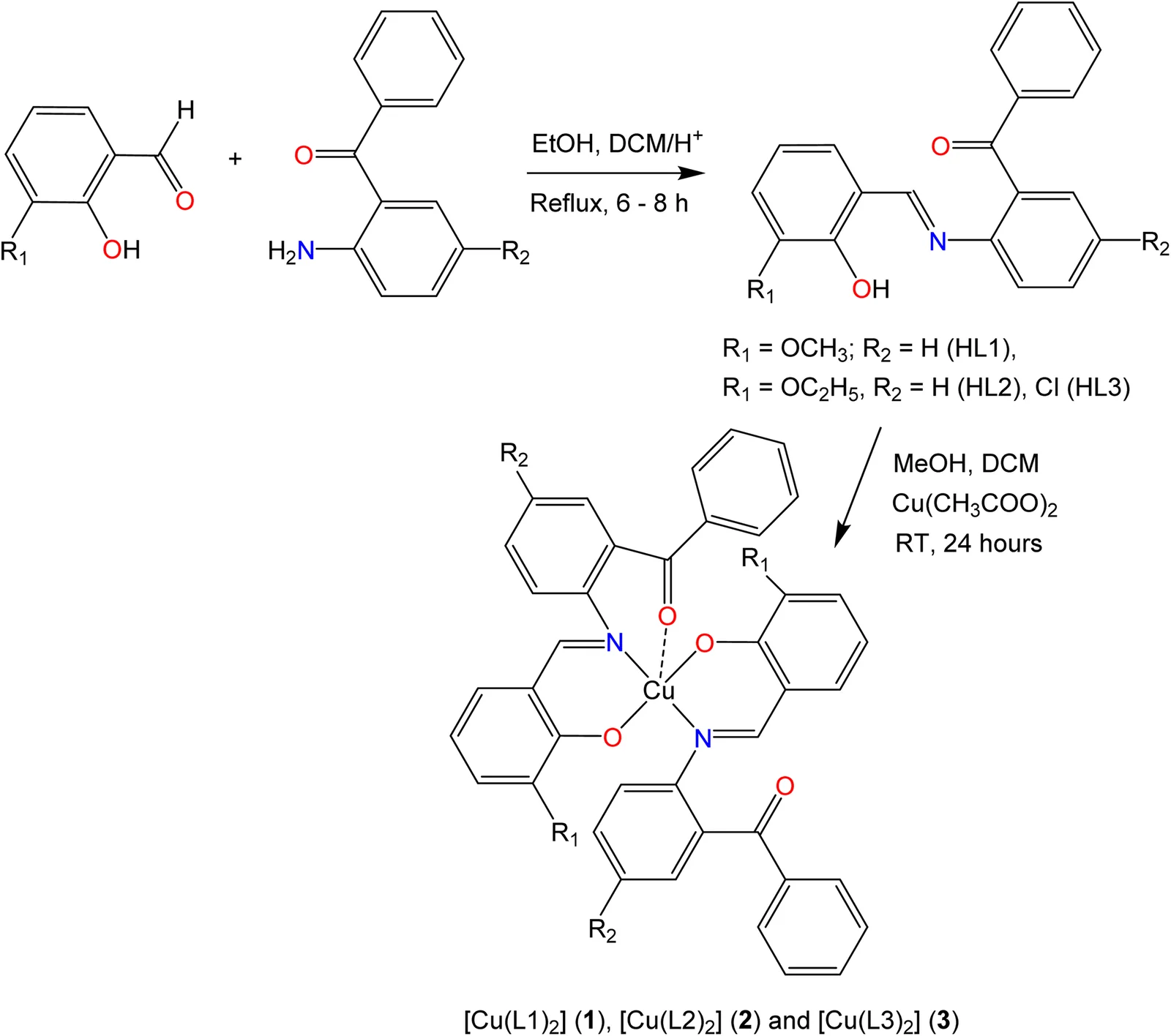
Formation of the Schiff bases (HL1–HL3) and their copper(ii) complexes (1–3).

Three new Schiff base ligands (HL1, HL2 and HL3) were synthesized from 3-methoxy/ethoxy-salicylaldehyde and 2-amino-benzophenone/5-chlorobenzophenone, which in turn react with Cu(ii) salt to afford the mononuclear [Cu(L1)_2_] (1), [Cu(L2)_2_] (2) and [Cu(L3)_2_] (3) complexes (Scheme 1). FT-IR spectra of the free ligands show characteristic bands at 1659–1647 *ν*(C=O), 1612–1582 *ν*(C=N) and 1578–1560 cm^−1^*ν*(C=C), which undergo systematic shifts to 1665–1641, 1605–1591 and 1543–1541 cm^−1^, respectively, in complexes (Fig. S1). Low molar conductance values (*Λ*_m_ = 1.32–1.61 S cm^2^ mol^−1^ in DMF) confirm the non-electrolytic profile of the complexes, supporting neutral coordination species as proposed in Scheme 1. Mass spectrometric (EI or HR-ESI) data exhibit molecular ion peaks at *m*/*z* = 331 (HL1), 345 (HL2), 379 (HL3), 723 (1), 753 (2) and 822 (3), accompanied by characteristic fragment ions attributable to stepwise ligand dissociation and complex fragmentation pathways (Fig. S2).

### ^1^H/^13^C NMR studies

^1^H NMR spectra for the ligands (HL1–HL3) show the imine-proton (HC=N) peak at *δ* 8.90 (HL1), 8.90 (HL2) and 8.91 ppm (HL3) in DMSO-*d*_6_ (Fig. S3). HL1 exhibits a singlet at *δ* 3.73 ppm for –OCH_3_. HL2 or HL3 shows a triplet at *ca. δ* 1.29 ppm (*J*_HH_ = 6.8 Hz) for the CH_3_ and quartet at *ca. δ* 3.98 ppm (*J*_HH_ = 7.2 Hz) for the –OCH_2_ group. The phenolic O–H presents the highest downfield peak at *δ* 11.95 (HL1), 12.06 (HL2) and 11.77 ppm (HL3). The aromatic proton region shows multiple signals at *δ* 6.85–7.74 (HL1), 6.83–7.74 (HL2) and 6.83–7.76 ppm (HL3), corresponding to distinct proton environments in the Schiff bases.^3–5,43,44^ (see Experimental section and Fig. S3). A notable feature in the aromatic region is the proton at *para* position to the OH group, designated as H4, which is the highest upfield aromatic proton and appears as a triplet at *δ* 6.83–6.85 (*J*_HH_ = 8.0 Hz), suggesting the highest electron withdrawing inductive effect at this position. ^13^C NMR spectra (Fig. S4) show resonance at the highest downfield at *δ* 197.12 (HL1), 197.01 (HL2) and 195.55 ppm (HL3) for the C=O (C14), consistent with sp^2^-hybridized ketonic carbonyl functionality. The HC=N (C7) appears at *ca. δ* 164.82 (HL1), 164.93 (HL2) and 165.27 (HL3) ppm. The C–OH (C1) appears at *δ* 148.16 (HL1) and *δ* 147.29 ppm (HL2 or HL3), while the adjacent (C2) carbon resonates slightly up-field at *δ* 145–147 ppm. The aromatic-carbons show several signals at *δ* 116–137 (HL1), 118–137 (HL2) and 118–137 ppm (HL3), attributed to the salicylal- and benzophenyl-rings.^2,9,45^ The OCH_3_ group presents a signal at *δ* 56.19 ppm in HL1, while two signals appear at *δ* 64.54–64.53 (OCH_2_) and 15.14–15.11 pm (CH_3_) for the ethoxy group in HL2 or HL3.

### Experimental and calculated UV-vis. spectra

**Fig. 1:**
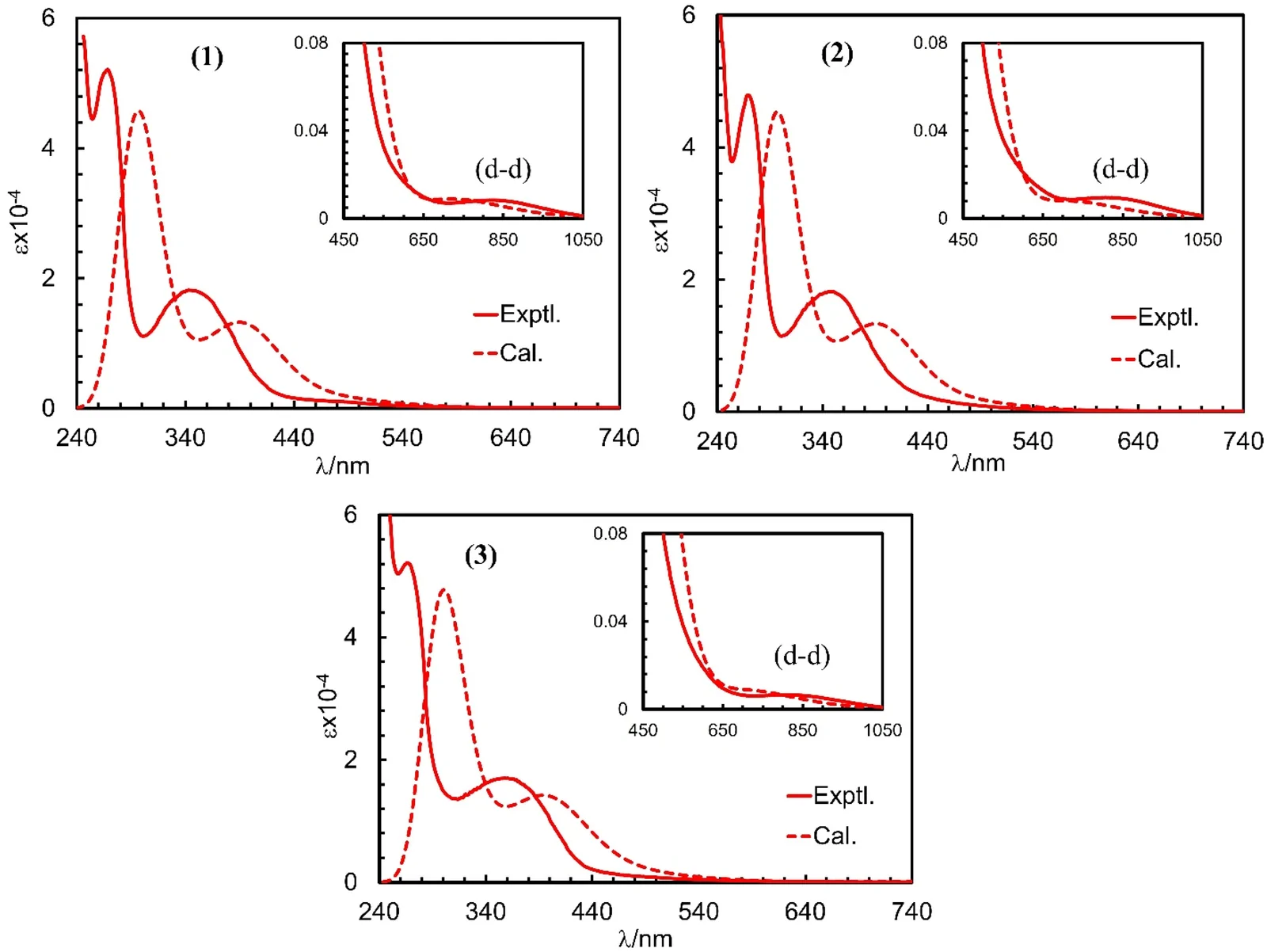
Experimental and calculated spectra for complexes 1 (*ca.* 0.029 mM), 2 (0.017 mM), and 3 (0.010 mM) in CHCl_3_ at 25 °C (d–d bands are presented in the inset). Spectra were calculated at M06/6-31G(d) with PCM in CHCl_3_ (*σ* = 0.33 eV).

**Fig. 2:**
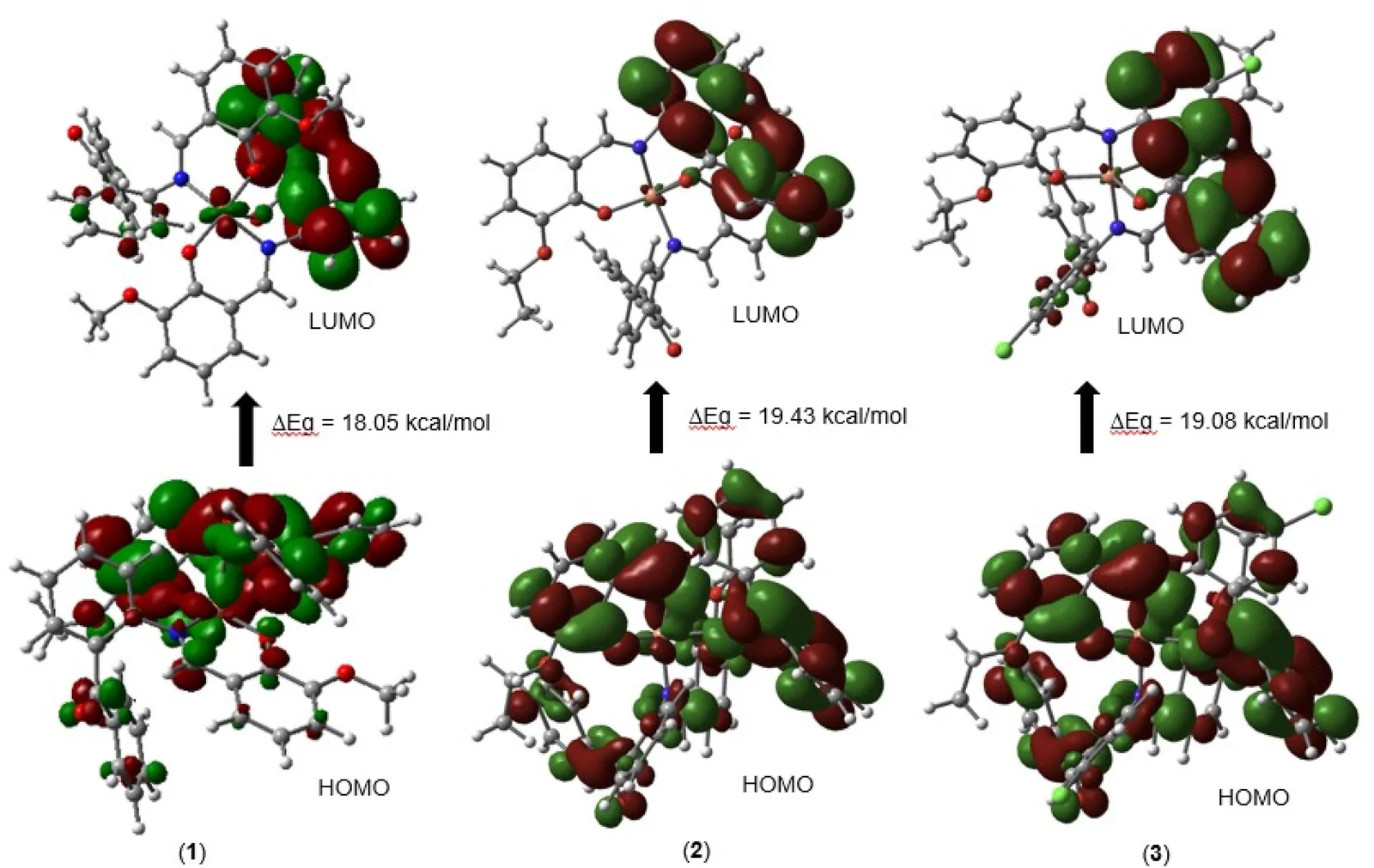
Illustration of HOMOs and LUMOs for the complexes (1–3). MO calculations were executed with β-spin electron excitation.

UV-vis. spectra (in CHCl_3_) for the pro-ligands and complexes are parallel (Fig. S5), with the complexes exhibiting an additional weak-broad band in the higher wavelength, attributed to the d–d transition of the Cu^2+^(d^9^) center (Fig. S5b, inset).^2–5,44,46–52^ However, two intense absorption bands were observed at <450 nm, attributed to intra-ligand n → π*/π → π* (LL) and metal–ligand charge transfer transitions (MLCT). To support the experimental assignments, TD-DFT calculations were performed for the complexes (Fig. 1 and S7). Though different combinations of exchange–correlation functionals (*e.g.*, B3LYP, CAM-B3LYP, and M06) and basis sets (*e.g.*, 6-31G(d), SDD, and SVP) were employed (Fig. S7), the final computational protocol such as M06/6-31G(d) was selected based on reasonable reproduction of the experimental spectra as shown in Fig. 1 (see Experimental section for detailed calculations). In this connection, some selected and simplified excited states including band maxima, oscillator strength and molecular orbital (MO) contributions were examined based on orbital and population assessments through MO calculations (Fig. 2 and Table 1).^11,12,43–46,49,50,52^ For quantitative comparison, the experimental absorption maxima (*λ*/max) are also listed in Table 1 along with the calculated data, showing little differences due to functional and basis set limitations, solvent effects and vibronic contributions. In addition, the calculated spectra were assigned based on gas phase geometry, while the experimental ones are based on the solid-state structure of the complexes. Assignment of excited state properties (Table 1) reveals that the excited state 8 (for example) shows a band at 489–496 nm for HOMO to LUMO electron excitation with the minimum energy gap (Δ*E*_g_) of 18.05–19.43 kcal mol^−1^. This band arises from combination of metal–metal (MM), ligand–metal/metal–ligand (LM/ML) and intra-ligand (LL) electron excitations with the highest MO contribution of 68–70% (*f* = 0.0187–0.0212). The HOMO is mainly localized with metal-d_*xy*_ and ligand-σ/π electron moieties, while the LUMO is with metal-d_*z*^2^_ (1) or metal-d_*xy*_ (2 or 3) and ligand-σ electron moieties (Fig. 2).

**Table 1:** Excited state properties (data) from TD-DFT calculations for the complexes (1–3)^a^

Bands^b^ (*λ*_max_/nm)	Excited state	Oscillator strength (*f*)	MO contributions^c^ (%)	Assignment^d^
**Complex**1				
725 (*ca.* 830)	4	0.0013	H – 16 → L (61)	LM, LL
489	8	0.0212	H → L (68)	MM, LM/ML, LL
403	13	0.0793	H → L + 2 (38), H → L + 3 (19)	MM, LM/ML, LL
351 (344)	31	0.0149	H – 3 → L (46), H – 1 → L + 4 (27)	MM, ML/LM, LL
300 (268)	51	0.1629	H – 8 → L (13), H – 2 → L + 3 (35)	LM, LL

**Complex**2				
777 (*ca.* 825)	2	0.0003	H – 23 → L (16), H – 15 → L (44)	LM, LL
715	4	0.0011	H – 16 → L (61)	LM, LL
490	8	0.0201	H → L (70)	MM, LM/ML, LL
405 (350)	13	0.0920	H → L + 2 (28), H → L + 3 (24)	MM, LM/ML, LL
300 (269)	51	0.2223	H – 8 → L (32), H – 2 → L + 3 (22)	MM, LM/ML, LL

**Complex**3				
768 (*ca.* 810)	2	0.0003	H – 15 → L (40), H – 27 → L (18)	LM, LL
714	4	0.0013	H – 16 → L (57)	LM, LL
496	8	0.0187	H → L (68)	MM, LM/ML, LL
412 (361)	13	0.1036	H – 1 → L + 1 (22), H → L + 3 (26)	MM, ML/LM, LL
304 (266)	50	0.1910	H – 11 → L (16), H – 6 → L (21)	LM, LL

a Spectra were calculated at M06/6-31G(d). MO calculations were executed with β-spin electron excitation.

b Experimental data are given in parentheses.

c H = HOMO/L = LUMO.

d MM = metal–metal, ML/LM = metal–ligand/ligand–metal and LL = ligand–ligand electrons excitation.

### Molecular structure

**Fig. 3:**
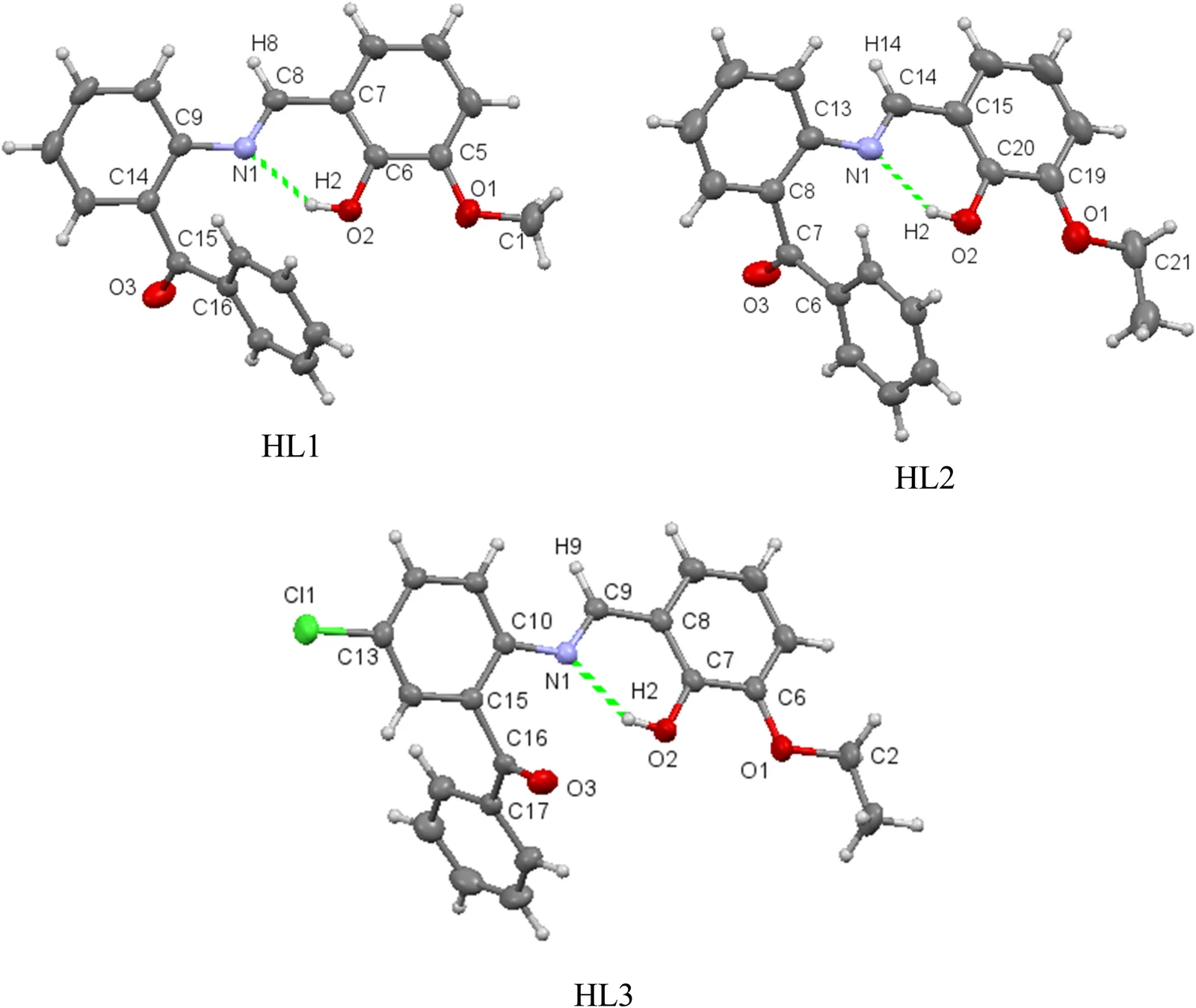
Molecular structures of HL1–HL3 (50% probability ellipsoids), with H atoms shown at arbitrary radii; intramolecular hydrogen-bonds are highlighted as green dashed lines.

In the solid-state, the Schiff base ligands crystallize in the triclinic *P*1̄ (HL1) or monoclinic *P*2_1_/*n* space group (HL2 and HL3) (Fig. 3). All Schiff base ligands adopt the usual enol-imine form with an O–H⋯N(imine) intramolecular hydrogen-bond with O2–H2⋯N1 = 1.89 Å (146°) in HL1, O2–H2⋯N1 = 1.89 Å (146°) in HL2 and O2–H2⋯N1 = 1.87 Å (146°) in HL3 (Fig. 3).^3,4,9,43,45^ The covalent skeleton structural parameters (Table 2) are in good agreement with those reported for related Schiff base ligands.^3,4,9,43–45^ There are significant intermolecular contacts among the symmetry related molecules with C18–H18⋯O3 = 2.42 Å (153°), founding a linear chain in HL1 and with C2–H2A⋯O1 = 2.43 Å (155°) and C12–H12⋯O1 = 2.47 Å (166°), resulting in a one-dimensional double strand in HL2 (Fig. S8). Interestingly, reciprocal C–H····O contacts are present between two inversion symmetric molecules with C14–H14⋯O3 = 2.38 Å (153°), establishing a *R*_2_^2^(10) ring according to the Etter notation in HL3 (Fig. S8). In the Etter symbolism *G*^a^_d_(*n*) the letter *G* encodes the motif (R = ring, C = chain, D = dimer or other finite set, S = intramolecular interaction), the subscript d gives the number of hydrogen-bond donors, the superscript a the number of hydrogen-bond acceptors, and the number *n* in parentheses gives the number of atoms involved in the motif.^53–55^ The structures are further assembled by C–H····π contacts with C4–H4⋯Cg = 2.84 Å (146°) (Cg represents the centroid of the C16–C21 ring) in HL1 and C18–H18⋯Cg = 2.80 Å (162°) (Cg represents the centroid of the C1–C6 ring) in HL2, both forming a linear chain motif, while with C1–H(1A)⋯Cg = 2.75 Å (158°) (Cg represents the centroid of the C10–C15 ring) making a zig-zag chain in HL3 (Fig. S9).

**Table 2:** Structural data of bond lengths/Å and angles/° in HL1–HL3

HL1				HL2			HL3		
Bond lengths, angles	X-ray structure	Opt. structure^a^	Opt. structure^b^	Bond lengths, angles	X-ray structure	Opt. structure^a^	Bond lengths, angles	X-ray structure	Opt. structure
O2–H2	0.840	1.011	1.021	O2–H2	0.840	1.011	O2–H2	0.840	0.996
O2–C6	1.346 (1)	1.357	1.363	O2–C20	1.345 (1)	1.358	O2–C7	1.350 (2)	1.335
N1–C9	1.416 (1)	1.417	1.423	N1–C14	1.280 (2)	1.305	N1–C9	1.286 (2)	1.292
N1–C8	1.286 (1)	1.305	1313	N1–C13	1.419 (2)	1.417	N1–C10	1.411 (2)	1.398
C1–O1	1.421 (2)	1.450	1.455	C7–O3	1.218(2)	1.252	C16–O3	1.215 (2)	1.223
C5–O1	1.365 (2)	1.385	1.390	C21–O1	1.440 (2)	1.460	C2–O1	1.435 (2)	1.425
O3–C15	1.217 (1)	1.252	1.259	O1–C19	1.364 (2)	1.382	O1–C6	1.365 (1)	1.360
H2–O2–C6	109.47	109.05	108.86	H2–O2–C20	109.5	109.01	Cl1–C13	1.740 (1)	1.758
C9–N1–C8	121.23 (9)	121.38	121.16	C14–N1–C13	121.2 (1)	121.34	H2–O2–C7	109.5	108.66
C5–O1–C1	118.80 (1)	118.40	118.36	C21–O1–C19	117.7(1)	119.02	C10–N1–C9	122.2 (1)	121.78
O3–C15–C14	118.30 (1)	118.70	118.73	O3–C7–C8	118.2 (1)	118.69	C2–O1–C6	117.1 (1)	118.58
O3–C15–C16	121.30 (1)	120.70	120.85	O3–C7–C6	121.7 (1)	120.67	O3–C16–C15	119.3 (1)	120.16
							O3–C16–C17	121.7 (1)	120.41
							Cl1–C13–C12	119.8 (1)	119.73
							Cl1–C13–C14	118.7 (1)	119.56

a Structure was optimized at: B3LYP/6-31G(d).

b B3LYP/LANL2DZ (both calculations show similar data).

**Fig. 4:**
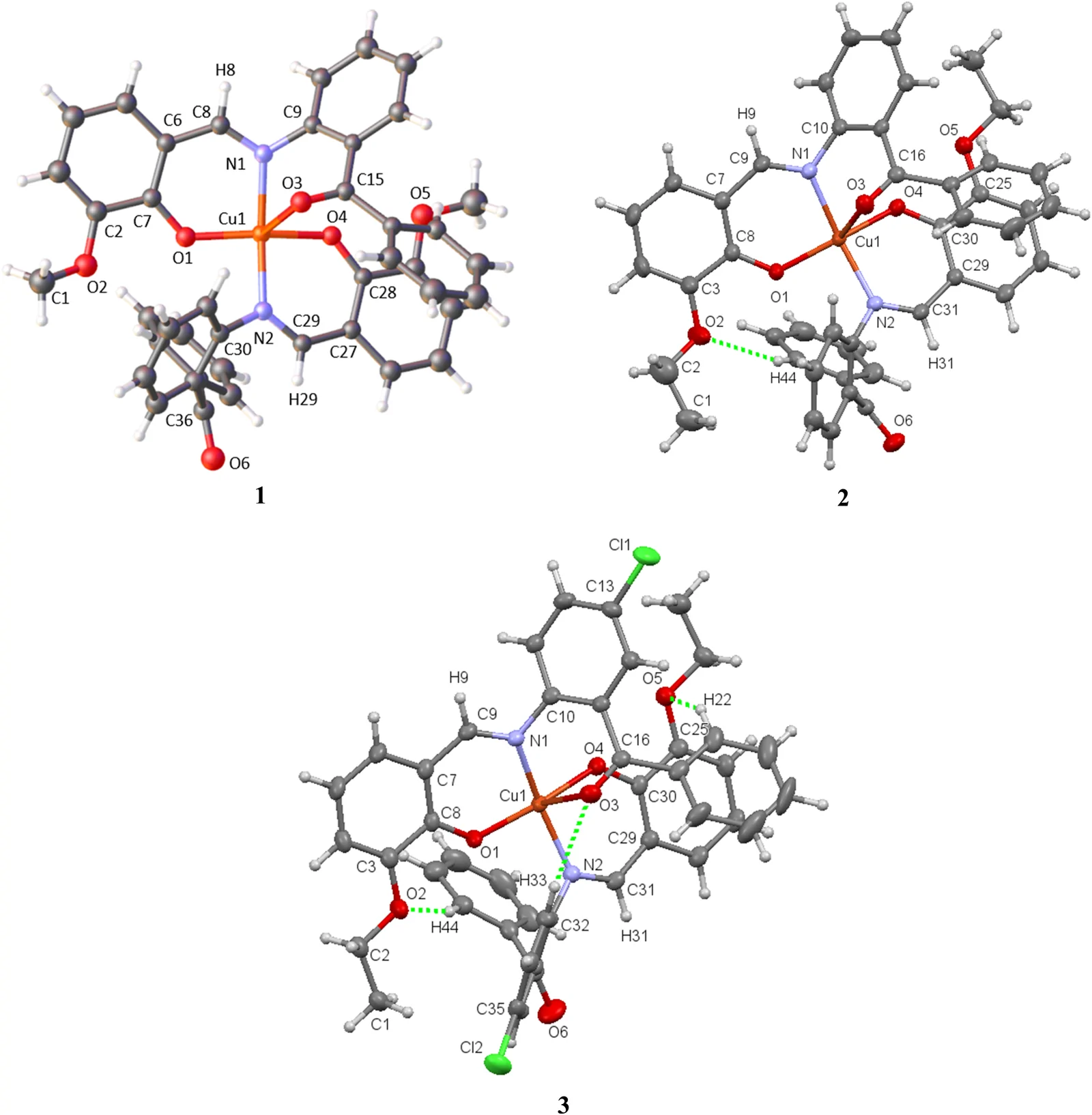
Molecular structures for complexes 1, 2, and 3 (50% thermal ellipsoids, H atoms with arbitrary radii). Significant intramolecular hydrogen-bonds are observed in complexes 2 and 3 (green dashed lines). The disorder in the ethoxy group in 2 is not shown for clarity.

All complexes crystallize in the monoclinic *Pc* space group (Fig. 4). In each case, only a single crystallographically independent molecule is present in the asymmetric unit, which thus contains a full formula unit. Single-crystal structural analysis reveals that the two *N*,*O*-chelating ligands bind to the Cu(ii) ion in a *trans-N*,*N*′ and *O*,*O*′ configuration. In addition, there is a fifth long bond between the Cu and O atoms of the benzoyl-keto group. This results in an overall square-pyramidal geometry at the Cu atom.^9,45,56–59^ If one considers only the four short Cu–O/N bonds of 1.86–2.00 Å around the Cu(ii) ion, the geometry is a distortion from square-planar to tetrahedral with dihedral angle (*θ*/°) values of 27.73° (1), 27.87° (2) and 23.30° (3) (Table 5). The additional elongated fifth Cu–O bond has a distance of 2.85 (1), 2.77 (2) and 2.79 Å (3). This reshapes the molecules to a distorted square-pyramidal geometry.^9,45,60–62^ Two geometric indices *τ*_4_ (ref. 63) and *τ*_5_ (ref. 64) have successfully been used to determine the square-planar and square-pyramidal geometry quantitatively, respectively (where *τ*_4_ = 0 for square-planar and *τ*_5_ = 0 for square-pyramidal). These parameters are nearly identical for 1 and 2, while they differ slightly from those of 3 (Table 3) due to the presence of intermolecular Cl⋯H halogen bond. However, the experimental and calculated values (from optimized structure) of these parameters differ for 1 (Table 5), as all the atoms and bonds are allowed to rotate freely during optimization in the gas phase, while there are constraints in the solid-state. Significant intramolecular C–H⋯O hydrogen-bonding interaction are observed with C44–H44⋯O2 = 2.57 Å (148°) in 2 and C22–H22⋯O5 = 2.59 Å (134°), C33–H33⋯O3 = 2.55 Å (158°) and C44–H44⋯O2 = 2.58 Å (166°) in 3 (Fig. 4).

**Table 3:** Structural data of bond lengths/Å and angles/° in 1, 2, and 3

Complex 1			Complex 2			Complex 3		
Bond lengths/Å, angles/°	X-ray structure	Opt. structure^a^	Bond lengths/Å, angles/°	X-ray structure	Opt. structure^a^	Bond lengths/Å, angles/°	X-ray structure	Opt. structure^a^
Cu1–O1	1.882 (4)	1.889	Cu1–O1	1.896 (2)	1.889	Cu1–O1	1.895 (3)	1.884
Cu1–O4	1.896 (4)	1.885	Cu1–O3	2.778 (2)	2.981	Cu1–O3	2.785 (2)	3.012
Cu1–N2	1.983 (5)	1.981	Cu1–O4	1.900 (2)	1.887	Cu1–O4	1.908 (3)	1.888
Cu1–N1	2.002 (5)	1.990	Cu1–N1	2.002 (2)	1.991	Cu1–N1	1.995 (3)	1.980
Cu1–O3	2.849	3.055	Cu1–N2	2.000 (2)	1.981	Cu1–N2	1.988 (3)	1.989
C36–O6	1.226 (6)	1.228	C25–O5	1.372 (3)	1.366	C25–O5	1.370 (5)	1.364
C15–O3	1.231 (7)	1.230	C3–O2	1.393 (6)	1.364	C3–O2	1.367 (5)	1.365
C2–O2	1.375 (6)	1.366	C38–O6	1.226 (3)	1.228	C38–O6	1.223 (5)	1.227
O1–Cu1–O4	159.7 (2)	148.64	O4–Cu1–O1	159.33 (8)	149.39	C13–Cl1/C35–Cl2	1.735 (4)/1.738 (5)	1.758/1.760
N2–Cu1–O1	88.4 (2)	93.63	O4–Cu1–N1	90.38 (8)	92.69	O4–Cu1–O1	164.1 (1)	149.94
O1–Cu1–N1	93.9 (2)	93.52	O4–Cu1–N2	92.69 (8)	93.80	O4–Cu1–N1	90.7 (1)	93.04
O4–Cu1–N2	92.6 (2)	93.85	O1–Cu1–N2	90.52 (8)	92.88	O4–Cu1–N2	92.9 (1)	93.52
O4–Cu1–N1	92.1 (2)	92.87	O1–Cu1–N1	93.39 (8)	93.40	O1–Cu1–N2	88.5 (1)	92.62
N1–Cu1–N2	160.0 (2)	154.12	N2–Cu1–N1	160.46 (8)	155.64	O1–Cu1–N1	92.8 (1)	93.72
						N2–Cu1–N1	162.2 (1)	154.95
						Cl1–C13–C14/Cl1–C13–C12	119.7 (3)/119.6 (3)	119.59/119.57
						Cl2–C35–C36/Cl2–C35–C34	120.2 (3)/118.6(3)	119.81/119.53
*θ* b (°)	27.73	39.80		27.85	38.30		23.30	38.30
*τ*_4_ c	0.285	0.406		0.285	0.39		0.239	0.39
*τ*_5_ d	0.005	0.091		0.019	0.104		0.031	0.084

a Structure was optimized at B3LYP/6-31G(d).

b *θ* (°) = dihedral angle between the two chelating planes, N1–Cu1–O1 and N2–Cu1–O4.

c *τ*_4_ = [{360 − (*α* + *β*)}/141].

d *τ*_5_ = [(*β* − *α*)/60] (where *α*, *β* are the two largest angles in four-coordinated species).

In contrast, the parallel copper(ii)-Schiff base complex to 1 or 3, lacking methoxy or ethoxy substituents on the salicylal-ring crystallized with a square-planar or distorted square-planar geometry, with either half or one complete formula unit present in the asymmetric unit.^9^ Further, a similar complex to 3 without an ethoxy group on the naphthalyl-ring instead of salicylal-ring yielded a square-pyramidal geometry with two symmetry-independent molecules in the asymmetric unit cell.^9^ However, the methoxy-substituted analogue of 3 on the salicylaldehyde ring forms polymorphs under two distinct crystallization conditions, both exhibiting square-planar geometry.^7^ The unit cell of polymorph-1 contains a single unique molecule, while polymorph-2 contains two symmetry-independent molecules. In each case, the molecules are located on inversion centers, resulting in asymmetric units containing either half or a complete formula unit. The overall results suggest that methoxy/ethoxy substituents play an important role in governing the coordination geometry, leading to square-pyramidal structures for the complexes (1–3). The structural data listed in Table 5 are in good accord with the optimized structure (Fig. S6), and parallel to the reported Cu(ii) complexes with similar Schiff base ligands.^9,45,56–62^ Supramolecular packing is governed by strong intermolecular C–H⋯O interactions between symmetry-related molecules with C11–H11⋯O3 = 2.57 Å (133°) in 1, C34–H34⋯O5 = 2.56 Å (143°) in 2 and C12–H12⋯O3 = 2.47 Å (142°) and C34–H34⋯O5 = 2.49 Å (153°) in 3 (Fig. S10). Notably, compound 3 is further organized by an intermolecular C–H⋯Cl(–C) and C–H····π contacts with C43–H43⋯Cl1 = 2.86 Å (C–H⋯Cl 149°, H⋯Cl–C 112°) and C28–H28⋯Cg = 2.82 Å (136°) (Cg represents the centroid of the C32–C37 ring), respectively (Fig. S11). The C–H⋯Cl contact of 2.86 Å is at the shorter end of C–H⋯Cl^0^ distances, which are seen for C–H⋯Cl hydrogen-bonds. The sum of the van der Waals radii of H and Cl atoms is 2.95 to 3.0 Å. Also, C–H⋯Cl angles can deviate from linearity down to 100°.^65^

### Hirshfeld surfaces

**Fig. 5:**
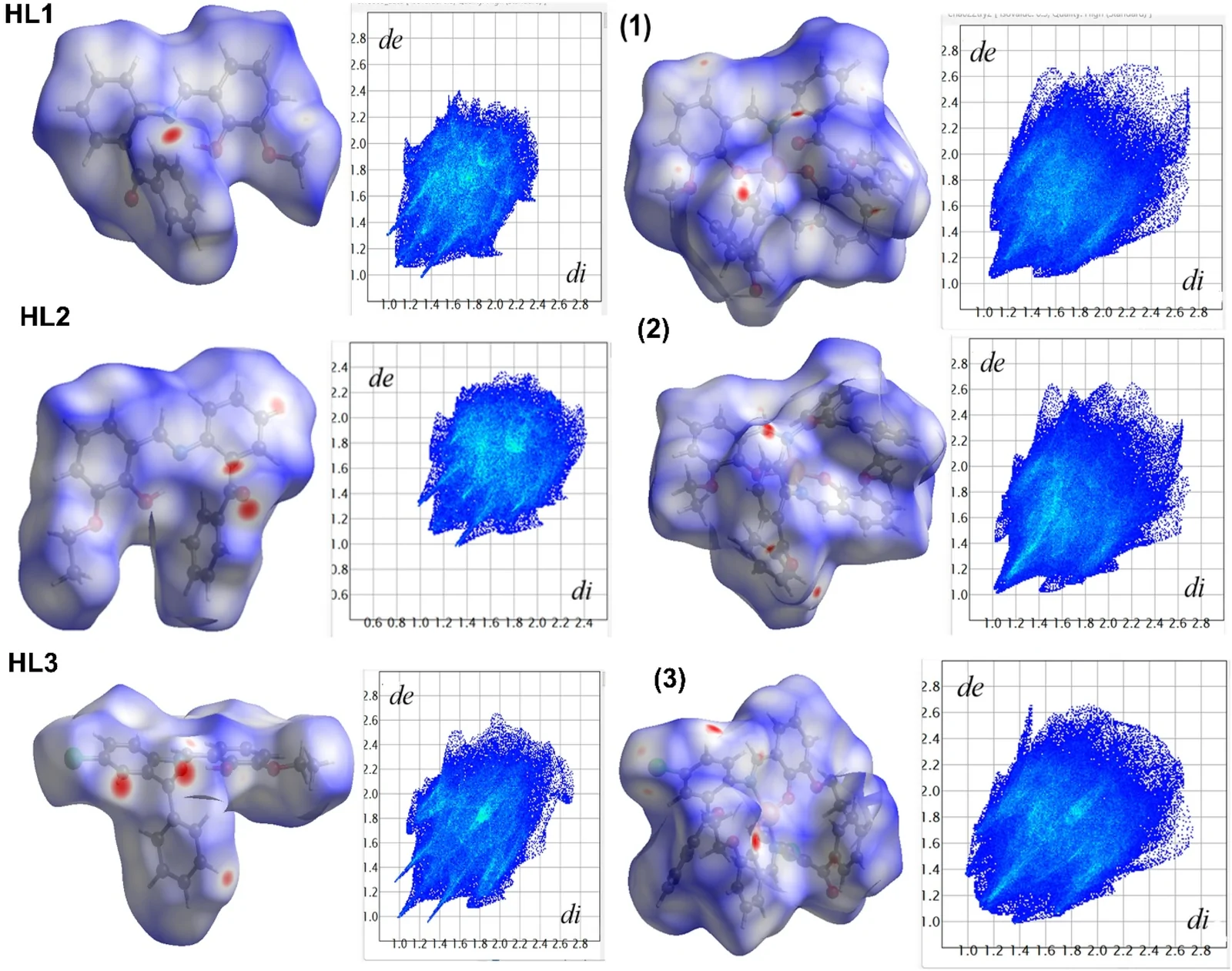
Hirshfeld surfaces mapped over *d*-norm property (left side: red and blue regions represent shorter and longer contacts, respectively) along with the 2D fingerprint plots (right side: displaying all intermolecular contacts) for HL1–HL3 and 1–3. The *di* and *de* give the distances from the nearest internal and external atoms to Hirshfeld surfaces, respectively.

Hirshfeld surface analyses using CrystalExplorer^66–70^ were carried out for the free ligands (HL1–HL3) and copper complexes (1–3) to achieve detailed information on intermolecular contacts directing crystal packing within the unit cell. The Hirshfeld surfaces mapped over *d*-norm property, along with 2D fingerprint plots for the ligands and complexes, are presented in Fig. 5. Detailed breakdowns of all intermolecular contacts and their contributions (%) are provided in Fig. S12a–c, S13a–c, and S14. In all cases, H⋯H contacts are the predominant interactions, contributing *ca.* 49% (HL1), 49% (HL2), and 40% (HL3), which increase upon complexation to *ca.* 52% (1), 56% (2), and 45% (3), respectively (Fig. S14). This increase shows the greater surface exposure of hydrogen atoms in the complex, arising from coordination of phenolic-oxygen and imine-nitrogen atoms to the metal ion. The second most important contributions arise from C⋯H contacts, which are generally linked with weak C–H⋯π interactions and edge-to-face aromatic contacts. They represent *ca.* 22 (HL1), 26 (HL2) and 27% (HL3) of the ligand supramolecular interactions, which rise to *ca.* 31% (1), 29% (2) and 27% (3) in the copper complexes. Notably, the Cl⋯H interaction of 8% (HL3) increased to 10% (3), resulting from electrostatically driven halogen···hydrogen interactions, possibly aided by conformational changes upon coordination. This highlights the character of halogen substituents in guiding precise interactions within the crystal matrix. The O⋯H contacts, arising from both classical (*e.g.*, O–H⋯X) and non-classical hydrogen-bonding, are more prominent in the free ligands (*ca.* 20% in HL1, 19% in HL2, and 16% in HL3) than those in the corresponding complexes (*ca.* 14% in 1, 12% in 2, and 10% in 3). This decrease is attributed to the involvement of phenolic- and carbonyl-oxygen atoms in metal coordination, which reduces their accessibility as hydrogen-bond acceptors in crystal packing. Interestingly, C⋯C interactions, indicative of π⋯π stacking, decrease from *ca.* 7% (HL1), 4% (HL2), and 2% (HL3) to approximately 1% in the complexes, representing a pronounced reduction upon complexation. This trend suggests that coordination induces steric and geometric constraints that hinder effective π–π stacking interactions.

### Powder XRD (PXRD)

**Fig. 6:**
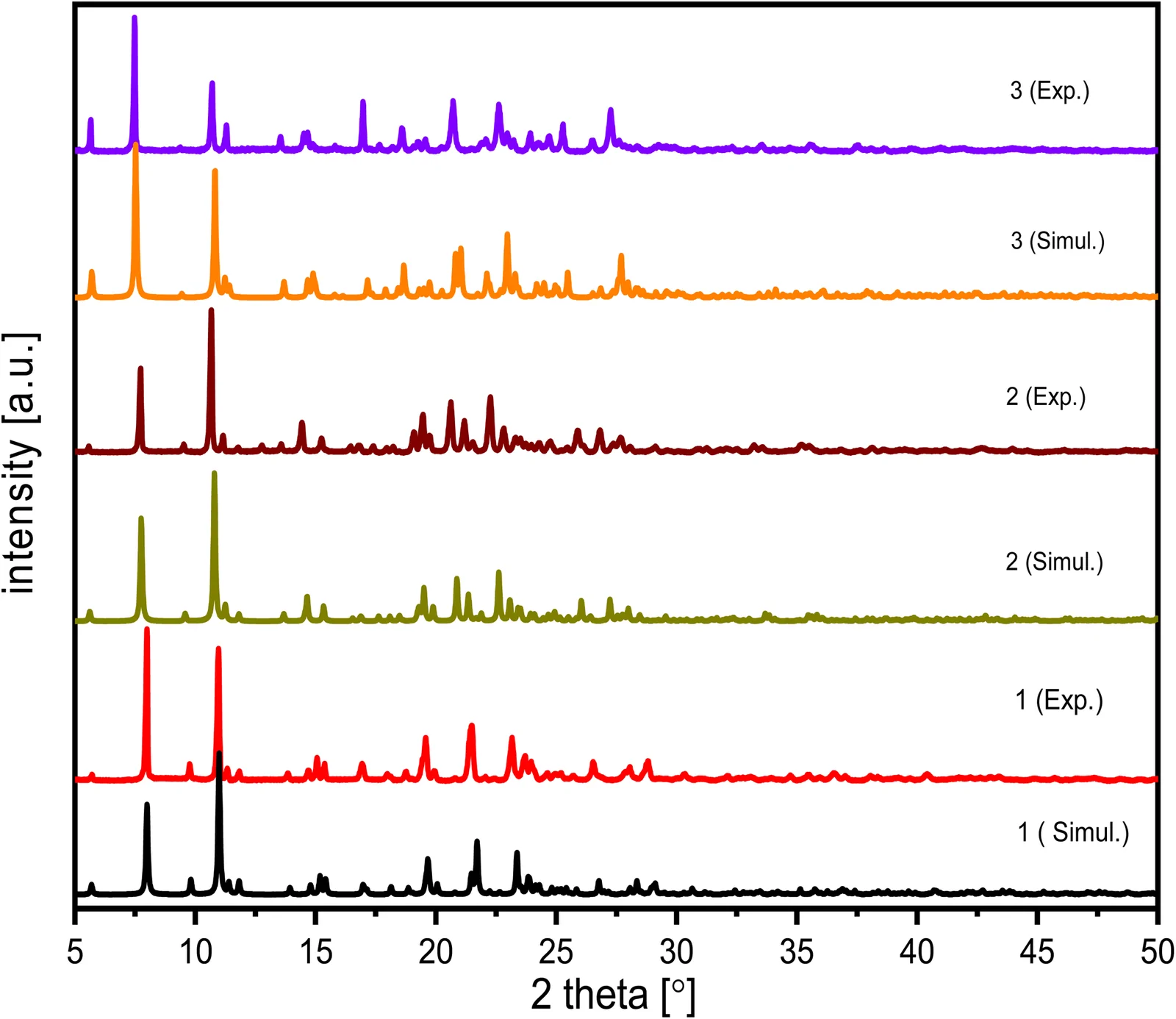
Comparison of experimental and simulated (from X-ray structure) PXRD profiles for the complexes (1–3).

Powder X-ray diffraction patterns were recorded for microcrystals of 1, 2 and 3 over 5–50° (2*θ*) at 298 K (Fig. 6). The experimental patterns show good alignment with the simulated profiles derived from X-ray crystal structures using Mercury,^71^ confirming the phase purity and integrity of bulk microcrystalline samples. The slight shift of the simulated peaks toward higher 2*θ* values is attributed to the deeper temperature used for X-ray data collection (100–200 K), which leads to shrinkage of unit-cell parameters (bond lengths and angles) in the crystal structure.^2,4,12,44,50,72^

### Thermal analyses

**Fig. 7:**
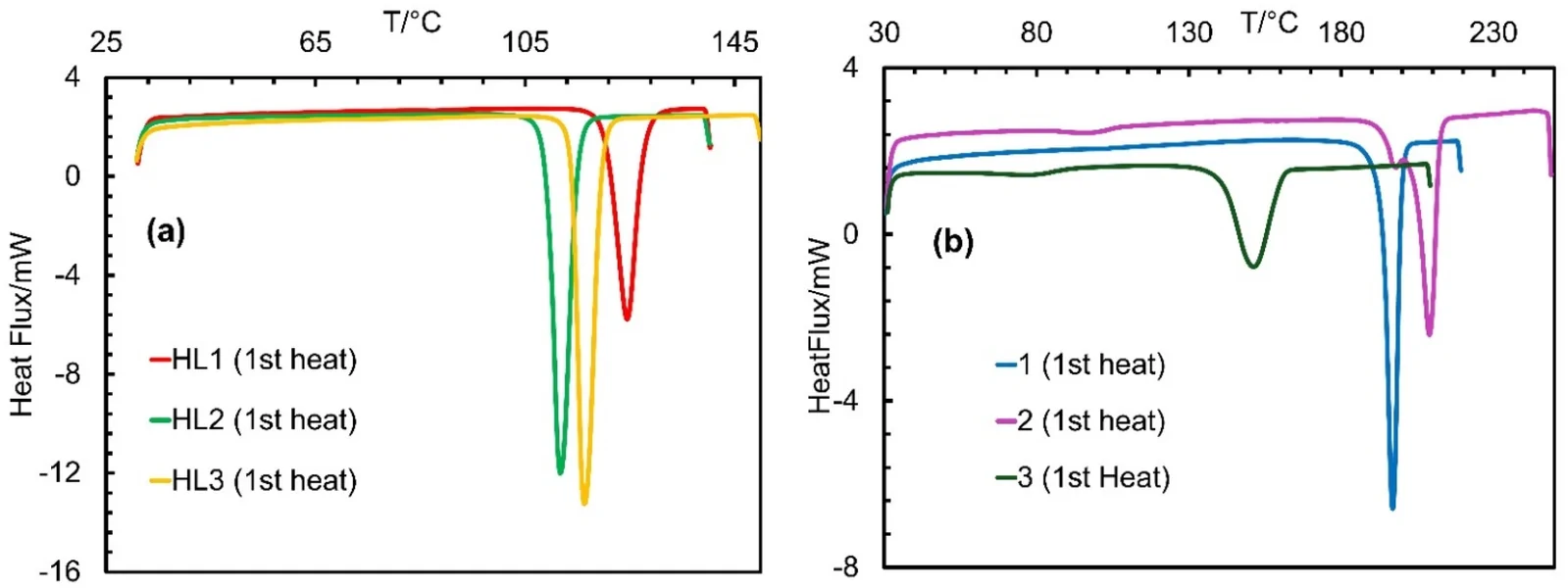
DSC heating curves for (a) Schiff bases and (b) Cu(ii) complexes.

Differential scanning calorimetry (DSC) was employed to investigate the thermal behaviors of the Schiff bases and complexes, featuring phase change temperatures (*T*/°C) and associated enthalpy change (Δ*H*) (Fig. 7 and Table 4).^2,4,9,44,45,50^ DSC heating profiles revealed a single sharp endothermic peak at *ca.* 124 °C (Δ*H* = −18.31 kJ mol^−1^ for HL1), 112 °C (Δ*H* = −28.72 kJ mol^−1^ for HL2) and 116 °C (Δ*H* = −27.16 kJ mol^−1^ for HL3), corresponding to melting point and a phase change from solid crystals to isotropic-liquid (Cs ⇆ I or m.p.). The second heating cycle DSC profiles show no detectable thermal events for HL1 and HL2, indicating the absence of recrystallization or phase transitions upon reheating, while HL3 displays two peaks: (i) a weak-broad exothermic peak at *ca.* 88 °C (Δ*H* = +1.75 kJ mol^−1^), attributing to a crystalline solid-to-cold crystalline (Cs ⇆ Cc) phase transformation, and (ii) a strong endothermic event at *ca.* 116 °C (Δ*H* = −12.47 kJ mol^−1^), corresponding to melting associated with the cold-crystalline to isotropic-liquid (Cc ⇆ I) (Fig. S15). The heating profiles for complexes 1 and 3 are identical and display a sharp endothermic peak at *ca.* 197 °C (Δ*H* = −42.48 kJ mol^−1^) and *ca.* 151 °C (Δ*H* = −32.80 kJ mol^−1^), respectively, corresponding to melting accompanied by a phase change from crystalline-solid to isotropic-liquid (Cs ⇆ I). On the other hand, complex 2 exhibits two endothermic peaks at *ca.* 198/209 °C (Δ*H* = −0.92/–42.63 kJ mol^−1^), assigned for microcrystals to solid-crystal (Cr ⇆ SCr) and then to isotropic-liquid (SCr ⇆ I) (Table 6). The repeated heating curves in the second run (for the same disc) produce a small endothermic peak at 196 °C (Δ*H* = −1.33 kJ mol^−1^ for 1) and 204 °C (Δ*H* = −9.56 kJ mol^−1^ for 2), assigned for Cs ⇆ I phase transition (Fig. S15 and Table 6), while no peaks are observed for 3. Comparison of the DSC data reveals a clear effect of metal coordination on the thermal behaviors of the compounds. While the free ligands (HL1–HL3) exhibit phase transitions in the range of 112–124 °C, the corresponding complexes undergo transitions at significantly higher temperatures (151–209 °C), indicating enhanced thermal stability upon complex formation, in parallel to higher molecular mass.

**Table 4:** DSC data (phase transition temperature, *T* and enthalpy change, Δ*H*) for the compounds

Compounds	Cycles	Heating curve: *T* (°C)/Δ*H* (kJ mol^−1^)
HL1 (microcrystals)	1st	124/−18.31 (Cs ⇆ I)
HL2 (microcrystals)	1st	112/−28.72 (Cs ⇆ I)
HL3 (microcrystals)	1st	116/−27.16 (Cs ⇆ I)
	2nd	88/+1.75 (Cs ⇆ Cc), 116/–12.47 (Cc ⇆ I)
1 (microcrystals)	1st	197/−42.48 (Cs ⇆ I)
	2nd	196/−1.33 (Cs ⇆ I)
2 (microcrystals)	1st	198/−0.92 (Cr ⇆ SCr), 209/–42.63 (SCr ⇆ I)
	2nd	204/−9.56 (Cs ⇆ I)
3 (single crystals)	1st	151/−32.80 (Cs ⇆ I)
	2nd	No peak

### Cyclic voltammetry (CV)

**Fig. 8:**
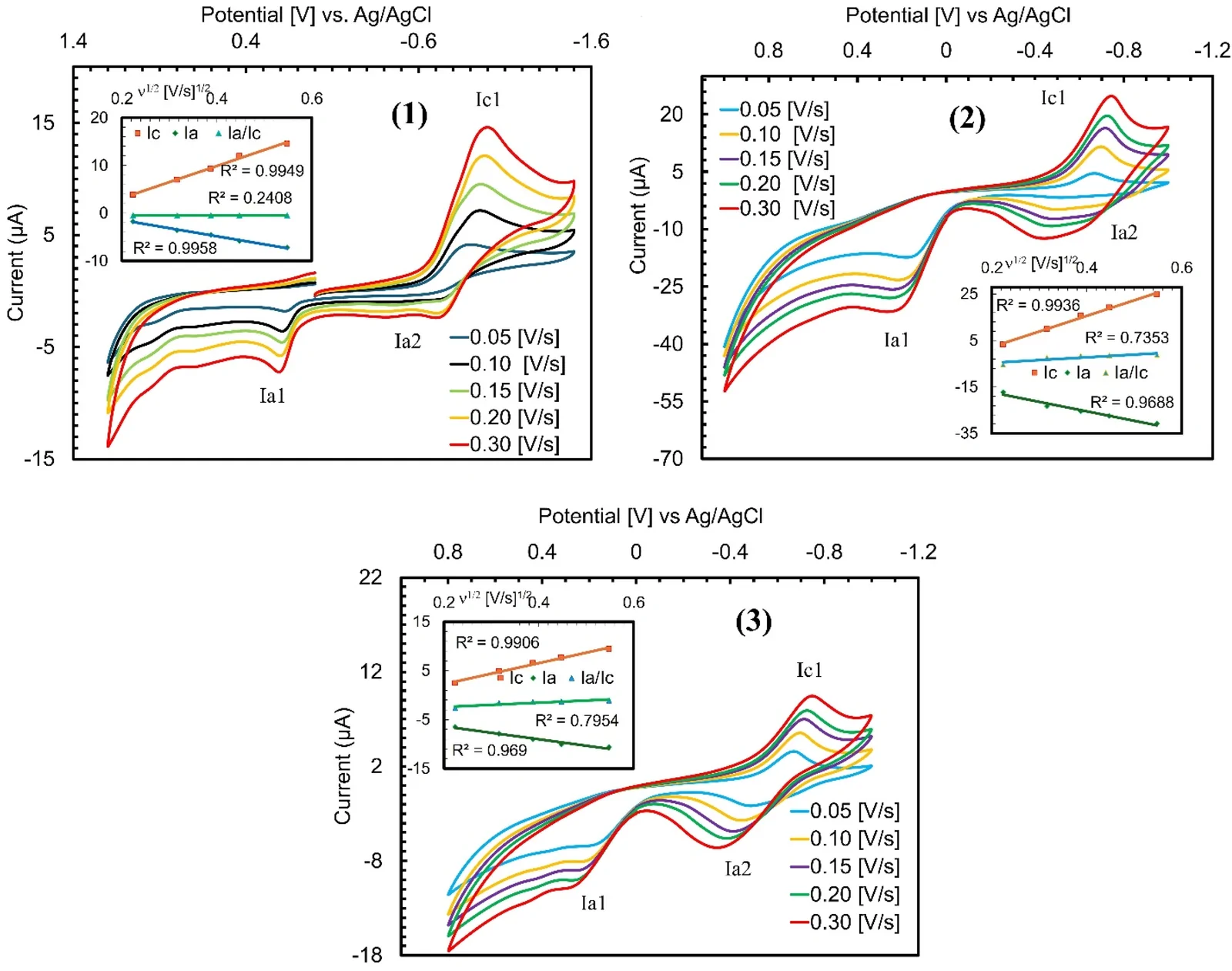
CV profiles for complexes 1–3 (*ca.* 0.50 mM) recorded at various scan rates in DMF at RT using TBAP (*ca.* 0.10 mol L^−1^) as supporting electrolyte (potential direction followed from zero to negative and then to positive).

Cyclic voltammetry measurements of the complexes were performed in DMF at 25 °C, over a potential window of −1.50 to +1.20 V *vs.* Ag/AgCl at varying scan rates (*ν* = 0.05–0.30 V s^−1^) (Fig. 8 and Table S1). All complexes exhibit similar CV profiles, consistent with quasi-reversible behavior, involving two successive one-electron charge-transfer processes corresponding to the Cu(ii)/Cu(i) and Cu(i)/Cu(0) redox couples in solution as observed for related Cu(ii)-Schiff base systems.^4,9,44,49,50,73^ The CV profiles feature two anodic peaks at the range of *E*_a1_ = +0.20 to +0.26 V (*I*_a1_ = −3.56 to −23.24 µA) and *E*_a2_ = −0.32 to −0.74 V (*I*_a2_ = −0.71 to −4.7 µA) with peak separation values of 0.95–1.20 V (*i.e.*, Δ*E*_1_ = *E*_a1_ − *E*_c1_), while a single broad cathodic peak at *E*_c1_ = – 0.74 to −1.00 V (*I*_c1_ = +4.90 to +9.92 µA) (Table S1). The broad cathodic peak may be assigned to the Cu(ii)/Cu(i) reduction process and reflects structural reorganization around the Cu center following electron transfer. The large peak separation values (Δ*E*_1_ = 0.95–1.20 V), significantly exceeding those expected for a reversible one-electron process, support the quasi-reversible nature of the redox event and indicate slow electron-transfer kinetics. Similar broad cathodic waves and large peak separations have been reported for the related Cu(ii)-Schiff base complexes.^4,9,44,49,50,73^ Notably, an increase in scan rate leads to an increase or decrease of peak current (*I*_c_ or *I*_a_), yielding linear plots of *I*_c_ or *I*_a_*versus ν*^1/2^, while the *I*_a_/*I*_c_ ratio remains essentially constant. These results advocate a diffusion-controlled redox profile for the complexes in solution.^4,9,44,49,50,73^ Although DMF is a coordinating solvent, the reproducible voltammetric responses (Fig. 8) obtained at varying scan rates suggest the electrochemical integrity and stability of the complexes under the experimental conditions and exclude any chance for replacement of the coordinated O-donor by DMF.

### Magnetic measurements

Solid-state magnetic susceptibility (*χ*_m_) and magnetic moment (*µ*_eff._) values are 1.16 × 10^−3^ (1), 1.20 × 10^−3^ (2), 9.87 × 10^−4^ cm^3^ mol^−1^ (3) and 1.67 (1), 1.70 (2), 1.54 *µ*_B_ (3), respectively (excluding the diamagnetic contribution from the ligand). These values prove paramagnetic features of the Cu(ii) complexes.^43,44^ The spin-only magnetic moment for (d^9^)Cu^2+^ is 1.73 *µ*_B_ (*S* = 1/2), the observed deviations from this value (*e.g.*, – 0.06 for 1, – 0.03 for 2 and – 0.19 *µ*_B_ for 3) might be due to orbital-angular momentum and/or diamagnetic contribution from ligands.^43,44^

### Antioxidant activities

**Fig. 9:**
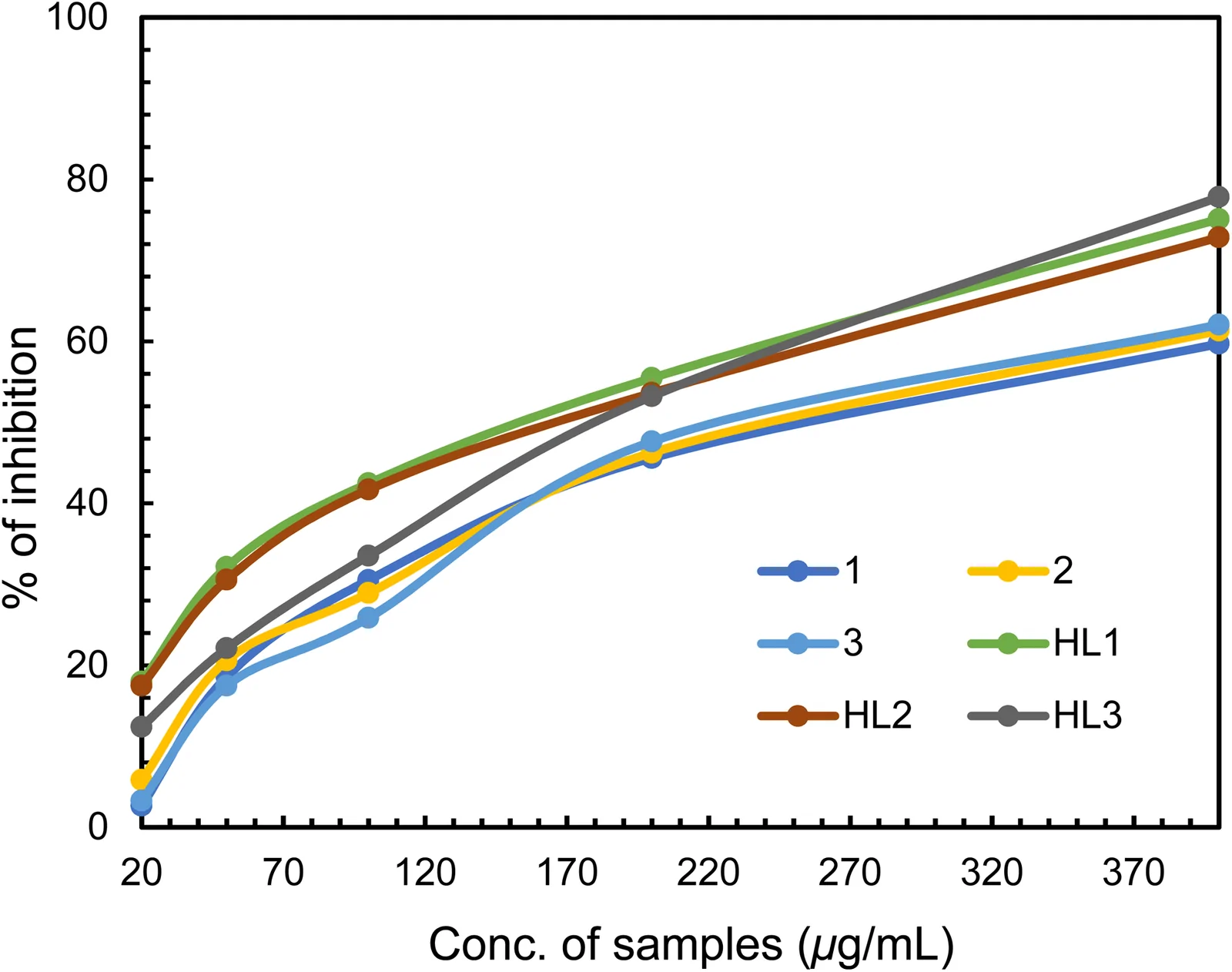
DPPH radical scavenging behaviors (% inhibition) of Schiff bases (HL1–HL3) and complexes (1–3) at various concentrations.

DPPH assay was employed to estimate antioxidant behaviors of Schiff bases and complexes (Fig. 9 and Table S2). The Schiff bases exhibited IC_50_ values of 193–215 µg mL^−1^, indicating moderate radical-quenching ability, whereas the complexes demonstrated relatively higher IC_50_ values of 282–390 µg mL^−1^, aligned with reduced activity upon metal coordination. This decline in activity is attributed to steric and electronic restriction of redox-active donor sites within the coordination sphere, which suppresses hydrogen-atom and electron transfer pathways essential for DPPH reduction.^2,4,34,35^ To ascertain whether the antioxidant process induced any structural or electronic changes in the Cu(ii) complex, its stability under the DPPH assay conditions was further investigated using spectroscopic methods (Fig. S16 and S17). The results indicate that the Cu(ii) complex remains structurally stable throughout the DPPH assay without significant decomposition or alteration of the coordination environment.

## Conclusion

Three new Schiff bases (HL1–HL3) and their copper complexes (1–3) were mainly investigated by X-ray diffraction along with different analytical tools. The free Schiff bases adopt an enol-imine form, stabilized by an intramolecular O–H⋯N(imine) hydrogen-bond. The complexes exhibit a *trans-N*,*N*′ and -*O*,*O*′ arrangement around the metal center with an elongated fifth Cu–O bond from the carbonyl-O to the copper(ii) ion, which gives a square-pyramidal coordination polyhedron. In contrast, similar complexes to 1 or 3 without methoxy or ethoxy substituents on the salicylal-ring provided a square-planar or distorted square-planar geometry. Experimental and simulated PXRD profiles show good agreement, confirming phase purity and integrity of bulk microcrystalline samples, similar to the single crystals. Supramolecular and Hirshfeld surface analyses reveal that molecular packing is primarily organized by H⋯H and C–H⋯π contacts, with an additional Cl⋯H contact in the chlorophenyl complex 3. CV measurements reveal a quasi-reversible electrochemical process involving two successive one electron charge transfer steps in DMF. DPPH studies demonstrate that free Schiff bases exhibit higher antioxidant activity than those of the complexes. Furthermore, the experimental absorption spectra and molecular structures are in good agreement with excited state properties by DFT/TD-DFT and molecular-orbital calculations. Overall, a small change in Schiff bases markedly influences coordination geometry, metal–ligand bonding, molecular structures and supramolecular assembly in the solid state. Future investigations will focus on ligand-design and metal ion selection to clarify their role in modulating structures, polymorphism, mesomorphism and physicochemical behaviors of the complexes.

## Experimental

### Materials and methods

IR spectra were recorded using FT-IR Prestige-21 spectrophotometer (Shimadzu) with KBr discs at ambient temperature. UV-vis. spectra were run with Shimadzu UV-1800 spectrophotometer in chloroform at 25 °C. Differential scanning calorimetry (DSC) data were recorded on DSC-60 (Shimadzu) within a temperature range of 30–300 °C and heating rate of 10 K min^−1^. ^1^H NMR/^13^C NMR spectra were recorded on a Bruker Avance DPX 400 spectrometer in DMSO-*d*_6_ at 20 °C. Elemental analyses were performed using a Vario EL analyzer from Elementar Analysensysteme. Electron ionization mass spectra (EI-MS) were recorded using Thermo-Finnigan TSQ 700 mass spectrometer, where isotopic distribution patterns for ^63^/^65^Cu-containing ions are clearly visible. Solid-state magnetic measurements were performed using the Magnetic Susceptibility Balance MSB Mk1 (Sherwood Scientific Ltd) at 25 °C. Molar conductance was measured in *N*,*N*-dimethylformamide (DMF) at 25 °C using Mettler Toledo FiveGo (Model F3) conductivity meter. Cyclic voltammetry experiments were conducted on Epsilo™ Instruments (BASi) system using tetra-*N*-butylammonium hexafluorophosphate (TBAP) as a supporting electrolyte in DMF at 25 °C. A three-electrode system consisting of a platinum disc working electrode, a platinum wire auxiliary electrode and an Ag/AgCl reference electrode was employed for CV measurements. The solution mixture was deoxygenated with nitrogen flow for *ca.* 10 minutes before measurements. Powder X-ray diffraction (PXRD) patterns were recorded on a GNR Explorer diffractometer equipped with Cu K_α_ radiation (*λ* = 1.5406 Å) operating at 40 kV and 30 mA, using the Bragg–Brentano geometry and a zero-background silicon sample holder. Data were collected over a 2*θ* range of 5–50° with a step size of 0.02° and a counting time of 3.0 s per step at 25 °C.

### Schiff base syntheses (HL1–HL3)

3-Methoxy-2-hydroxy-benzaldehyde or 3-ethoxy-2-hydroxy-benzaldehyde (1.52 g or 1.66 g, 10.0 mmol) was dissolved in methanol (10 mL). A few drops of glacial acetic acid were then added, and the solution was stirred for 20–25 min. A solution of either 2-aminobenzophenone (1.97 g, 10.0 mmol) or 2-amino-5-chlorobenzophenone (2.32 g, 10.0 mmol) in ethanol (5 mL) was added dropwise under continuous stirring. The resulting reaction mixture was refluxed for 6–8 h, during which the color gradually changed to orange. Upon completion of reaction, the solvent volume was reduced to approximately 50–60% in *vacuo* at 40 °C until the onset of precipitation. The mixture was then left standing in air for 24 h to allow for complete precipitation. The resulting precipitate was isolated by filtration, washed three times with methanol (3 × 3 mL) and dried in air for 3–4 hours to give yellow crystalline ligands (HL1–HL3). Single crystals for X-ray diffraction were grown by slow diffusion of *n*-hexane into concentrated dichloromethane solution of HL1–HL3 over 2–3 days at ambient temperature.

Data for HL1: yield: 3.0142 g (90.97%); m.p. 124 °C. IR (KBr, cm^−1^): *ν* = 3292m (br, O–H), 3069, 3032, 2995, 2951, 2936, 2901w (Ar–H), 1655*vs.* (C=O), 1612, 1593*vs.* (C=N) and 1578*vs.* (C=C) (Fig. S1). Anal. calcd for C_21_H_17_NO_3_: C, 76.12; H, 5.17; N, 4.23%. Found C, 76.46; H, 4.96; N, 4.65%. ^1^H NMR (DMSO-*d*_6_): *δ*/ppm = 3.73 (s, 3H, OCH_3_), 6.85 (t, *J*_HH_ = 8.0 Hz, 1H, H_4_), 7.07 (d, *J*_HH_ = 8.0 Hz, 1H, H_3_), 7.13 (dd, *J*_HH_ = 7.6, 0.8 Hz, 1H, H_5_), 7.46–7.55 (m, 4H, H_9,11,17,19_), 7.59 (d, *J*_HH_ = 8.0 Hz, 1H, H_18_), 7.64–7.68 (m, 2H, H_10,12_), 7.74 (d, *J*_HH_ = 7.2 Hz, 2H, H_16,20_), 8.90 (s, 1H, CNH) and 11.95 (s, H, OH) (Fig. S3). ^13^C NMR (DMSO-*d*_6_): *δ*/ppm = 56.19 (OCH_3_), 116.23 (C_3_), 119.14 (C_4_), 119.32 (C_6_), 119.54 (C_5_), 124.42 (C_9_), 127.27 (C_11_), 128.65 (C_13_), 129.34 (C_17,19_), 129.96 (C_16,20_), 132.05 (C_12_), 134.21 (C_18_), 134.96 (C_10_), 137.17 (C_15_), 146.58 (C_2_), 148.16 (C_1_, –OH), 150.46 (C_8_), 164.82 (C_7,_ –C=N) and 197.12 (C_14_, –C=O) (Fig. S4). EI-MS: *m*/*z* (%) = 331 (100) [HL1]^+^, 302 (68) [HL1-OCH_3_ + H_2_]^+^, 181 (30) [C_13_H_9_O]^+^, 105 (46) [C_7_H_6_O–H]^+^ and 77 (74) [C_6_H_5_]^+^ {HL1 = C_21_H_17_NO_3_} (Fig. S2a).

Data for HL2: yield: 2.872 g (83.14%); m.p. 111 °C. IR (KBr, cm^−1^): *ν* = 3451m (br, O–H), 3055, 3034, 2980, 2930, 2889w (Ar–H), 1659*vs.* (C=O), 1614, 1593*vs.* (C=N) and 1578*vs.* (C=C) (Fig. S1). Anal. calcd for C_22_H_19_NO_3_: C, 76.50; H, 5.54; N, 4.06%. Found C, 76.88; H, 5.33; N, 4.39%. ^1^H NMR (DMSO-*d*_6_): *δ*/ppm = 1.29 (t, *J*_HH_ = 6.8 Hz, 3H, CH_3_), 3.98 (q, *J*_HH_ = 7.2 Hz, 2H, OCH_2_), 6.83 (t, *J*_HH_ = 8.0 Hz, 1H, H_4_), 7.05 (d, *J*_HH_ = 8.0 Hz, 1H, H_3_), 7.14 (d, *J*_HH_ = 8.0 Hz, 1H, H_5_), 7.46–7.49 (m, 2H, H_11,19_), 7.53 (t, *J*_HH_ = 7.6 Hz, 2H, H_17,18_), 7.59 (d, *J*_HH_ = 8.0 Hz, 1H, H_9_), 7.64–7.70 (m, 2H, H_10,12_), 7.74 (d, *J*_HH_ = 7.6 Hz, 2H, H_16,20_), 8.90 (s, 1H, CNH) and 12.06 (s, H, OH) (Fig. S3). ^13^C NMR (DMSO-*d*_6_): *δ*/ppm = 15.14 (CH_3_), 64.53 (OCH_2_), 117.72 (C_4_), 119.13 (C_3_), 119.41 (C_6_), 119.69 (C_5_), 124.63 (C_9_), 127.18 (C_11_), 128.71 (C_13_), 129.32 (C_17,19_), 130.01 (C_16,20_), 132.07 (C_12_), 134.18 (C_18_), 134.83 (C_10_), 137.21 (C_15_), 146.65 (C_2_), 147.29 (C_1_, –OH), 150.83 (C_8_), 164.93 (C_7,_ –C=N) and 197.01 (C_14_, –C=O) (Fig. S4). EI-MS: *m*/*z* (%) = 345 (33) [HL2]^+^, 330 (36) [HL2-OH + H_2_]^+^, 181 (31) [C_13_H_9_O]^+^, 152 (58) [C_9_H_12_O_2_]^+^, 105 (93) [C_7_H_6_O–H]^+^ and 77 (100) [C_6_H_5_]^+^ {HL2 = C_22_H_19_NO_3_} (Fig. S2a).

Data for HL3: yield: 3.055 g (80.45%); m.p. 120 °C. IR (KBr, cm^−1^): *ν* = 3449m (br, O–H), 3071, 3051, 2976, 2918, 2849w (Ar–H), 1647*vs.* (C=O), 1609, 1584*vs.* (C=N) and 1560*vs.* (C=C) (Fig. S1). Anal. calcd for C_22_H_18_ClNO_3_: C, 69.57; H, 4.78; N, 3.69%. Found C, 69.83; H, 4.60; N, 4.07%. ^1^H NMR (DMSO-*d*_6_): *δ*/ppm = 1.28 (t, *J*_HH_ = 6.8 Hz, 3H, CH_3_), 3.98 (q, *J*_HH_ = 7.2 Hz, 2H, OCH_2_), 6.83 (t, *J*_HH_ = 8.0 Hz, 1H, H_4_), 7.06 (d, *J*_HH_ = 8.0 Hz, 1H, H_3_), 7.12 (d, *J*_HH_ = 8.0 Hz, 1H, H_9_), 7.52–7.59 (m, 3H, H_5,10,17_), 7.65 (t, *J*_HH_ = 8.0 Hz, 2H, H_18,19_), 7.72–7.76 (m, 3H, H_12,16,20_), 8.91 (s, 1H, CNH) and 11.77 (s, H, OH) (Fig. S3). ^13^C NMR (DMSO-*d*_6_): *δ*/ppm = 15.11 (CH_3_), 64.54 (OCH_2_), 117.84 (C_4_), 119.24 (C_3_), 119.67 (C_6_), 121.28 (C_5_), 124.55 (C_9_), 128.10 (C_12_), 129.41 (C_17,19_), 130.06 (C_16,20_), 131.56 (C_18_), 131.73 (C_11_), 134.48 (C_13_), 136.64 (C_10_), 136.69 (C_15_), 145.45 (C_2_), 147.29 (C_1_, –OH), 150.75 (C_8_), 165.27 (C_7,_ –C=N) and 195.55 (C_14_, –C=O) (Fig. S4). EI-MS: *m*/*z* (%) = 379 (46) [HL3]^+^, 364 (68) [HL3-OH + H_2_]^+^, 181 (10) [C_13_H_19_O]^+^, 152 (40) [C_9_H_12_O_2_]^+^, 105 (92) [C_7_H_6_O–H]^+^ and 77 (100) [C_6_H_5_]^+^ {HL3 = C_22_H_18_ClNO_3_} (Fig. S2a).

### Complex syntheses (1–3)

The Schiff base HL1 (662.7 mg, 2.0 mmol) or HL2 (690.8 mg, 2.0 mmol) or HL3 (759.5 mg, 2.0 mmol) was dissolved in a solvent mixture of methanol (12 mL) and dichloromethane (3 mL). This reaction mixture was continuously stirred for *ca.* 15 min at ambient temperature to obtain a clear and homogeneous solution prior to further reaction. Subsequently, a methanolic solution (8 mL) of copper(ii) acetate (200.0 mg, 1.0 mmol) was added slowly to the ligand solution under constant magnetic stirring. The reaction mixture was then allowed to stir continuously for 24 h under a nitrogen atmosphere. During the reaction, a gradual color transformation to deep brown was observed, indicating the formation of the copper(ii) complex. Upon completion of the reaction, the solvent was evaporated under reduced pressure at 40 °C. The resulting solid product was collected and washed 3 times with methanol (3 mL in each), followed by *n*-hexane, to yield deep-brown crystalline complexes (1–3). The isolated complexes were air-dried for 3–4 days and preserved under nitrogen. Single crystals for X-ray diffraction were successfully grown by slow evaporation of concentrated methanolic or ethanolic solutions of the complexes (1–3) under ambient conditions over a period of 2–3 days.

Data for complex 1: yield: 0.496 g (74.86% based on HL1); m.p. 206 °C. Anal. calcd for C_42_H_32_CuN_2_O_6_: C, 69.65; H, 4.45; N, 3.87%. Found: C, 70.03; H, 4.56; N, 4.24%. IR (KBr, cm^−1^): *ν* = 3051, 2982, 2951, 2930, 2828w (*ν*C–H), 1659, 1641*vs.* (*ν*C=O), 1605, 1593*vs.* (*ν*C=N) and 1541*vs.* (*ν*C=C) (Fig. S1). EI-MS: *m*/*z* (%)=723 (7) [Cu(L1)_2_]^+^, 393 (100) [Cu(L1)_2_-L1]^+^, 331 (73) [HL1]^+^, 302 (40) [HL1-OCH_3_ + H_2_]^+^, 181 (21) [C_13_H_19_O]^+^, 105 (49) [C_7_H_6_O–H]^+^ and 77 (61) [C_6_H_5_]^+^ {[Cu(L1)_2_] = C_42_H_32_CuN_2_O_6_, HL1 = C_21_H_17_NO_3_} (Fig. S2b). *Λ*_m_ = 1.32 S cm^2^ mol^−1^ in DMF and *µ*_eff._ = 1.67 *µ*_B_ at RT.

Data for complex 2: yield: 0.561 g (81.23% based on HL2); m.p. 213 °C. Anal. calcd for C_44_H_36_CuN_2_O_6_: C, 70.25; H, 4.82; N, 3.72%. Found: C, 70.27; H, 4.93; N, 3.97%. IR (KBr, cm^−1^): *ν* = 3061, 2984, 2918, 2897, 2866w (*ν*C–H), 1661, 1641*vs.* (*ν*C=O), 1605, 1593*vs.* (*ν*C=N) and 1543*vs.* (*ν*C=C) (Fig. S1). ESI-MS (*m*/*z*): 752 {[Cu(L2)_2_ + H]^+^ = [C_44_H_36_CuN_2_O_6_+H]^+^} (Fig. S2b). *Λ*_m_ = 1.47 S cm^2^ mol^−1^ in DMF and *µ*_eff._ = 1.70 *µ*_B_ at RT.

Data for complex 3: yield: 0.594 g (78.21% based on HL3); m.p. 151 °C. Anal. calcd for C_44_H_34_Cl_2_CuN_2_O_6_: C, 64.35; H, 4.17; N, 3.41%. Found: C, 64.72; H, 4.33; N, 3.69%. IR (KBr, cm^−1^): *ν* = 3059, 2976, 2870w (*ν*C–H), 1653*vs.* (*ν*C=O), 1605*vs.* (*ν*C=N) and 1541*vs.* (*ν*C=C) (Fig. S1). ESI-MS (*m*/*z*): 822 {[Cu(L3)_2_ + H] = [C_44_H_34_Cl_2_CuN_2_O_6_ + H]^+^} (Fig. S2b). *Λ*_m_ = 1.61 S cm^2^ mol^−1^ in DMF and *µ*_eff._ = 1.54 *µ*_B_ at RT.

### X-ray measurements

Single crystals of HL1, HL2, HL3, and complexes 2 and 3, appropriate for X-ray diffraction studies were selected manually under an optical microscope, coated with inert oil, and mounted on cryo-loops. Diffraction measurements were obtained using a Rigaku XtaLAB Synergy R four-circle diffractometer equipped with a HyPix hybrid pixel array detector and a PhotonJet X-ray source operating with Cu-Kα radiation (*λ* = 1.54184 Å) together with a multilayer mirror monochromator. Crystals of complex 1 were mounted on MiTeGen loops, and intensity data were collected under a nitrogen cold stream at 150 K using a Bruker D8 QUEST ECO diffractometer with Mo-Kα radiation. All instrument operations and data acquisition procedures were performed with the Bruker APEX3 software package.^74^ The structures were solved and refined using the XS, XT, and XL routines integrated within the OLEX2 software environment.^75^ The possibility of higher crystallographic symmetry was examined for each structure with the ADDSYM routine implemented in PLATON.^76^ Diffraction data for HL1, HL2, HL3, and complexes 2 and 3 were recorded at approximately 170 K, whereas data for complex 1 were collected at around 150 K using ω-scan methods. Data processing, including reduction and absorption corrections, was carried out with CrysAlisPro 1.171.42.96a (Rigaku OD, 2023).^77^ Crystal structures were determined by direct methods using SHELXT-2018/2,^78^ and full-matrix least-squares refinement against *F*^2^ was performed using SHELXL-2017/1 within OLEX2.1.3.^75,78,79^ Hydrogen atoms bonded to carbon were positioned in calculated geometries and refined using riding models. Standard bond distances of 0.95 Å for aromatic and methine C–H groups, 0.99 Å for CH_2_ groups, and 0.98 Å for CH_3_ groups were applied. Corresponding isotropic displacement parameters were fixed at 1.2*U*_eq_ for CH and CH_2_ groups and 1.5*U*_eq_ for CH_3_ groups. Crystallographic parameters and refinement details are summarized in Tables 5 and 6. Molecular graphics were generated using the Mercury visualization program.^71^ The supplementary crystallographic information associated with this work has been deposited with the Cambridge Crystallographic Data Centre under deposition numbers 2489907–2489910, 2489913 and 2539031. This data is available free of charge from the Cambridge Crystallographic Data Centre (CCDC) *via*https://www.ccdc.cam.ac.uk/data_request/cif.

**Table 5:** Crystal data and structure refinement for the Schiff bases HL1–HL3

Complexes	HL1	HL2	HL3
Data code	data_tx18009_auto	data_fx2244_auto	data_tx18010_auto
Empirical formula	C_21_H_17_NO_3_	C_22_H_19_NO_3_	C_22_H_18_ClNO_3_
*M* (g mol^−1^)	331.36	345.38	379.82
Crystal size (mm)	0.40 × 0.35 × 0.30	0.28 × 0.24 × 0.20	0.42 × 0.35 × 0.32
Temperature (K)	170.1(4)	170.0(1)	170.0(3)
*θ* range (°)	4.00–77.63	3.89–76.19	2.97–77.24
*h*; *k*; *l* range	+9, −8; +12, −13; +14, −12	±18; +7, −9; +20, −19	±14; +18, −16; +14, −15
Crystal system	Triclinic	Monoclinic	Monoclinic
Space group	*P*1̄	P2_1_/*n*	P2_1_/*n*
*a* (Å)	7.2956(2)	14.9044(3)	11.5601(2)
*b* (Å)	10.3018(3)	7.3831(1)	14.8531(2)
*c* (Å)	11.1424(3)	16.1475(3)	11.9495(2)
*α* (°)	96.280(2)	90	90
*β* (°)	97.113(2)	94.502(2)	112.835(2)
*γ* (°)	91.176(2)	90	90
*V* (Å^3^)	825.52(4)	1771.40(6)	1890.96(6)
*Z*	2	4	4
*D*_calc_ (g cm^−3^)	1.333	1.295	1.334
*F* (000)	348	728	792
*µ* (mm^−1^)	0.723	0.694	1.970
Max/min transmission	1.000/0.8637	1.0000/0.5168	1.000/0.5251
Refl. measured	9364	21 368	12 937
Refl. unique (*R*_int_)	3324 (0.0172)	3604 (0.0512)	3880 (0.0290)
Data/restraints/parameters	3324/0/228	3604/0/237	3880/0/246
Completeness	0.995	0.970	0.999
Largest diff. peak & hole (Δ*ρ*/e Å^−3^)	0.266/−0.158	0.289/−0.229	0.289/−0.260
*R*_1_/w*R*_2_ [*I* > 2*σ*(*I*)] a	0.0368/0.1012	0.0480/0.1267	0.0380/0.1030
*R*_1_/w*R*_2_ (all reflect.) a	0.0389/0.1027	0.0526/0.1319	0.0402/0.1045
Goodness-of-fit on *F*^2^^b^	1.046	1.074	1.034
CCDC number	2489907	2539031	2489908

a *R*_1_ = [Σ(‖*F*_o_|−|*F*_c_‖)/Σ|*F*_o_|]; w*R*_2_ = [Σ[w(*F*_o_^2^ − *F*_c_^2^)^2^]/Σ[w(*F*_o_^2^)^2^]]^1/2^.

b Goodness-of-fit, *S* = [Σ[w(*F*_o_^2^ − *F*_c_^2^)^2^]/(*n* − *p*)]^1/2^.

**Table 6:** Crystal data and structure refinement for the complexes 1, 2 and 3

Complexes	1	2	3
Data code	data_ena022try2	data_tx18011_auto	data_tx18012_auto
Empirical formula	C_42_H_32_CuN_2_O_6_	C_44_H_36_CuN_2_O_6_	C_44_H_34_Cl_2_CuN_2_O_6_
*M* (g mol^−1^)	724.23	752.29	821.17
Crystal size (mm)	0.18 × 0.09 × 0.05	0.28 × 0.24 × 0.20	0.20 × 0.20 × 0.18
Temperature (K)	150.00	170(2)	169.9(4)
*θ* range (°)	2.63–27.62	2.81–77.41	2.82–76.79
*h*; *k*; *l* range	+14, −15; ±20; ±12	±14; +13, −19; +11, −12	±15; +18, −19; +12, −9
Crystal system	Monoclinic	Monoclinic	Monoclinic
Space group	*Pc*	*Pc*	*Pc*
*a* (Å)	11.584(3)	11.79686(18)	12.5014(2)
*b* (Å)	15.514(4)	15.7176(2)	15.4856(2)
*c* (Å)	9.859(2)	9.93433(14)	10.24163(17)
*α* (°)	90	90	90
*β* (°)	107.471(9)	105.1120(15)	110.0953(19)
*γ* (°)	90	90	90
*V* (Å^3^)	1690.1(7)	1778.30(4)	1861.99(5)
*Z*	2	2	2
*D*_calc_ (g cm^−3^)	1.423	1.405	1.465
*F* (000)	750	782	846
*µ* (mm^−1^)	0.700	1.307	2.588
Max/min transmission	0.7456/0.5961	1.000/0.8295	1.000/0.2497
Refl. measured	43 546	11 819	12 575
Refl. unique (*R*_int)_	7523 (0.0814)	5439 (0.0226)	4981 (0.0219)
Data/restraints/parameters	75 232/2/463	5439/43/509	4981/2/498
Completeness	0.983	0.764	0.666
Largest diff. peak & hole (Δ*ρ*/e Å^−3^)	0.414/−0.580	0.287/−0.450	0.375/−0.506
*R*_1_/w*R*_2_ [*I* > 2*σ*(*I*)] a	0.0436/0.0882	0.0283/0.0767	0.0340/0.0917
*R*_1_/w*R*_2_ (all reflect.) a	0.0643/0.0994	0.0286/0.0769	0.0346/0.0922
Goodness-of-fit on *F*^2^^b^	1.066	1.041	1.051
CCDC number	2489909	2489910	2489913

a *R*_1_ = [Σ(‖*F*_o_|−|*F*_c_‖)/Σ|*F*_o_|]; w*R*_2_ = [Σ[w(*F*_o_^2^ − *F*_c_^2^)^2^]/Σ[w(*F*_o_^2^)^2^]]^1/2^.

b Goodness-of-fit, *S* = [Σ[w(*F*_o_^2^ − *F*_c_^2^)^2^]/(*n* − *p*)]^1/2^.

### Computational method

Computations were performed using Gaussian 09.^80^ The initial gas phase geometry for optimization was designed from the CIF files of the ligands and complexes, followed by optimization at B3LYP/6-31G(d) (Fig. S6). The optimized structures closely match the corresponding X-ray structures with minor deviations (see overlay structures in Fig. S18 and Tables 2, 3). This optimized structure was then subjected to time-dependent density functional theory (TD-DFT) at M06/6-31G(d) to calculate the UV-vis. spectra (Fig. 1). To check the reliability and validity of applied computational protocols, different combinations of the functionals (*e.g.*, B3LYP, CAM-B3LYP and M06) and basis sets (*e.g.*, 6-31G(d), SDD and SVP) were used for calculations of UV-vis. spectra for complex 1 (Fig. S7). Calculated spectra thus obtained are highly consistent and closely match the experimental spectra with a minor shift in the band's position (Fig. 1 and S7). However, the best fit of calculated spectra to the experimental one was observed at M06/6-31G(d), which was then followed for complexes 2 and 3. Solvent effects were minimized through the polarizable continuum model (PCM) using chloroform as solvent, and a total of 72 excited states (roots) were included in the calculations. The simulated spectra were produced using SpecDis,^81^ applying Gaussian band shapes with a half-width of *σ* = 0.33 eV. The molecular orbital (MO) calculation was made for the determination of assignments to some selected excited states (bands) and electronic transitions using the same theoretical method employed in the analysis of orbitals and populations (Fig. 2 and Table 1). The Cartesian coordinates of the optimized structures of the Schiff bases (HL1–HL3) and complexes (1–3) are provided in the SI (see Tables S3–S9).

### DPPH radical scavenging studies

The free radical scavenging activity of the ligands and their corresponding Cu(ii) complexes was investigated using the DPPH (2,2-diphenyl-1-picrylhydrazyl) assay, a widely used method for evaluating antioxidant activity.^2,4,34,35^ A freshly prepared methanolic DPPH solution (*ca.* 0.1 mmol L^−1^) was kept in the dark prior to analysis. The working solutions were prepared in methanol at different concentrations ranging from 20 to 400 µg mL^−1^. For each test, 1 mL of working solution was mixed with 2 mL of DPPH solution and incubated at ambient temperature for 30 min under dark conditions. The absorbance was recorded at 517 nm using UV-vis. spectroscopy.

## Author contributions

IH: characterization, structural analysis, data curation, writing – review and editing, software and writing – original draft preparation. GA: synthesis, characterization, data analysis and writing – original draft preparation. KAT: characterization and writing – review and editing. YW: crystal structure determination, refinement and validation. YM: crystal structure determination, refinement and validation. WHS: crystal structure determination, refinement and validation. CJ: conceptualization, data interpretation, software, crystal structure determination, refinement and validation, funding acquisition and writing – review and editing. ME: conceptualization, supervision, characterization, structural analysis, project administration, software, writing – original draft preparation, funding acquisition and writing – review and editing.

## Conflicts of interest

The authors declare no competing interests.

## Supplementary Material

RA-016-D6RA04681A-s001

RA-016-D6RA04681A-s002

## Data Availability

CCDC 2489907–2489910, 2489913 and 2539031 contain the supplementary crystallographic data for this paper.^82*a*–*f*^ The data supporting this article have been included as part of the supplementary information (SI). Supplementary information: IR, MS, NMR, UV-vis spectra, optimized structures, intermolecular interactions, Hirshfeld surfaces, DSC, CV and IC_50_ data. See DOI: https://doi.org/10.1039/d6ra04681a.
