# Supplementary material for: Square-pyramidal copper(ii)-Schiff base complexes with methoxy/ethoxy-phenolate and chloro-benzophenone substituents
Source: RSC Adv. 2026 Jul 23. Online ahead of print. doi: 10.1039/d6ra04681a (PMC13394547; doi:10.1039/d6ra04681a)
Supplement: RA-OLF-D6RA04681A-s001 [file RA-OLF-D6RA04681A-s001.pdf]

Supplementary Information (SI) for RSC Advances.
This journal is © The Royal Society of Chemistry 2026

Square-pyramidal copper(II)-Schiff base complexes with methoxy/ethoxy-phenolate and chloro-benzophenone substituents

Imdadul Haque,[1] Galib Abdullah,[1] Kashfia Azad Tuba,[1] Yizhou Wang,[2] Yanping Ma,[2] Wen-Hua Sun,[2] Christoph Janiak,[3,*] Mohammed Enamullah,[1,4 *]

[1] Department of Chemistry, Jahangirnagar University, Dhaka-1342, Bangladesh.

[2] Institute of Chemistry, Chinese Academy of Sciences, Haidian, Beijing 100190, P. R. China.

[3] Institut für Anorganische Chemie und Strukturchemie, Heinrich-Heine-Universität, D 40204 Düsseldorf, Germany.

[4] Vice Chancellor, Hajee Mohammad Danesh Science & Technology University (HSTU), Dinajpur, Bangladesh.

[*] Correspondence: enamullah@juniv.edu

Emails of all authors: IH: hiraahamed44@gmail.com; GA: galib.stu2017@juniv.edu; KAT: kashfia12.azad@gmail.com; YW: wangyizhou13@iccas.ac.cn; YM: myanping@iccas.ac.cn; WHS: whsun@iccas.ac.cn; CJ: janiak@hhu.de; ME: enamullah@juniv.edu

Content:

Section S1 **Infrared spectra**
Section S2 **Mass spectra**
Section S3 **NMR spectra**
Section S4 **UV-vis spectra and optimized structures**
Section S5 **Intermolecular interactions**
Section S6 **Hirshfeld surfaces**
Section S7 **Differential scanning calorimetry (DSC)**
Section S8 **Stability assessment of the Cu complex (2) after the DPPH assay**
Section S9 **Cyclovoltammetry data**
Section S10 **IC_{50} values**
Section S11 **Cartesian coordinates of the optimized geometries**
Section S12 **Overlay plots of the X-ray and optimized structures**

Section 1: Infrared spectra

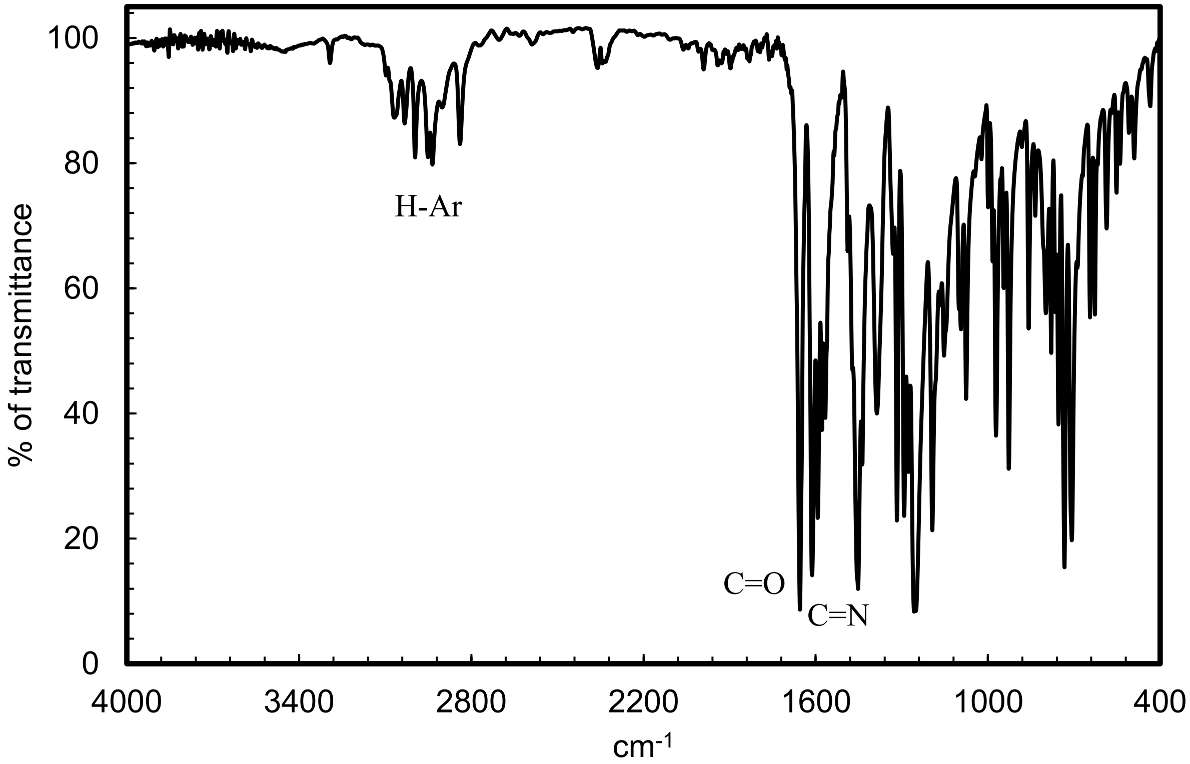

HL1

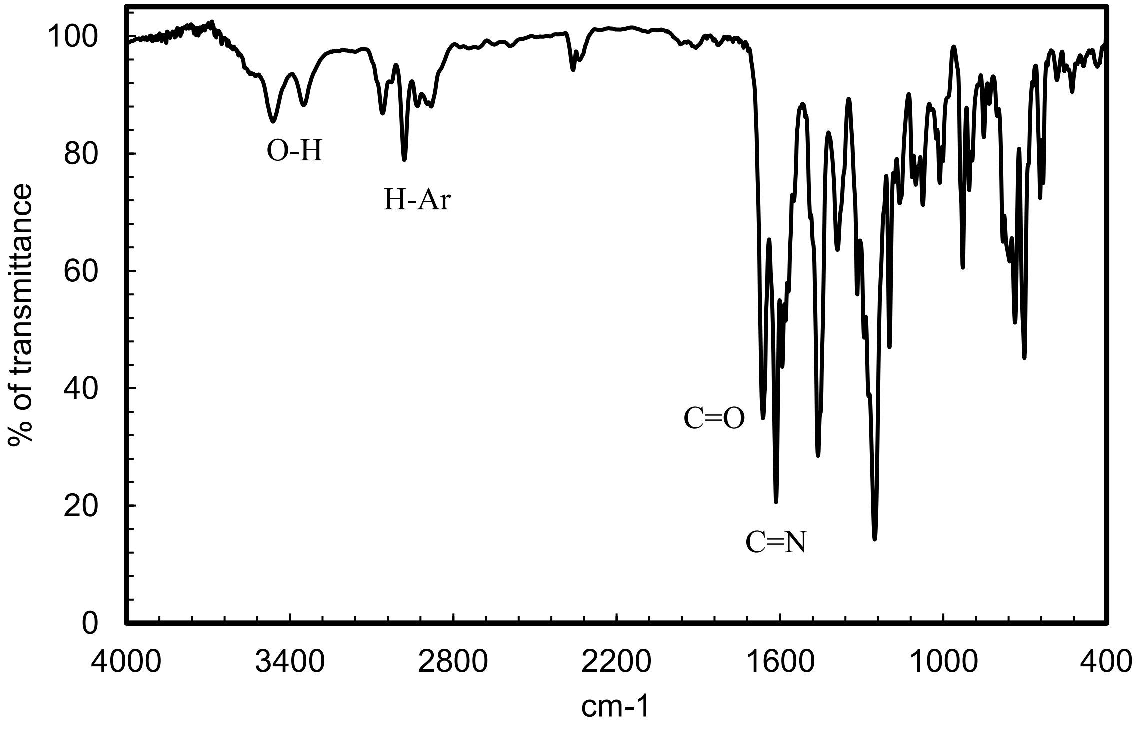

HL2

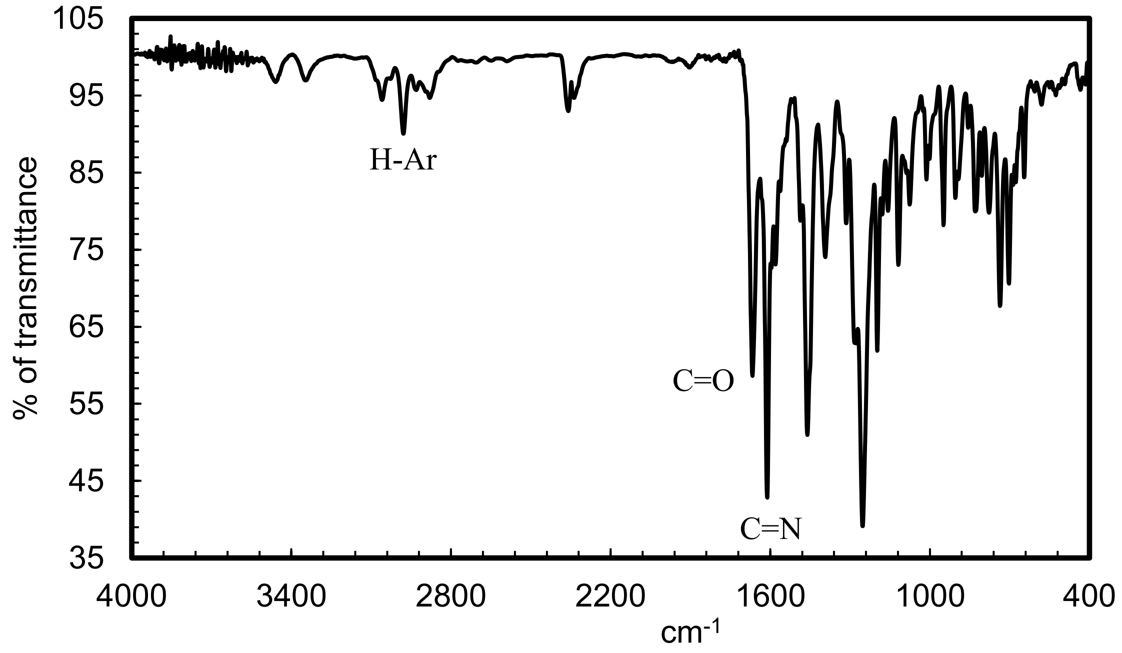

HL3

Fig. S1a. IR (KBr, cm^{-1}) spectra for the Schiff base ligands (HL1- HL3) at ambient temperature.

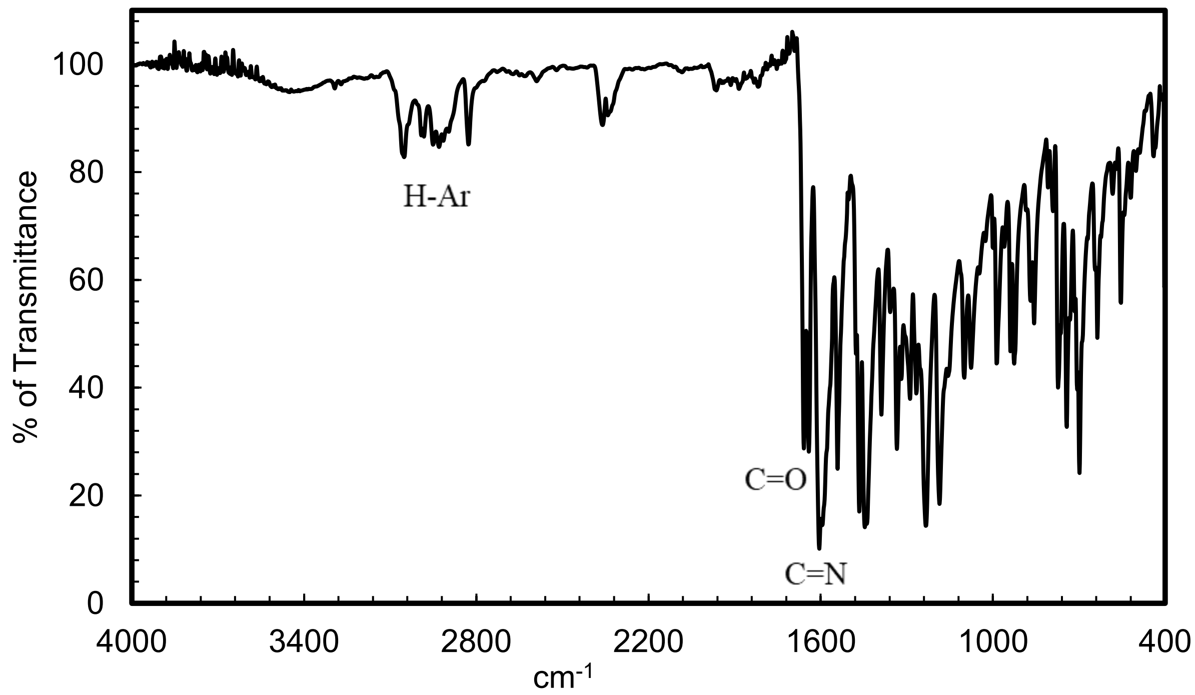

(**1**)

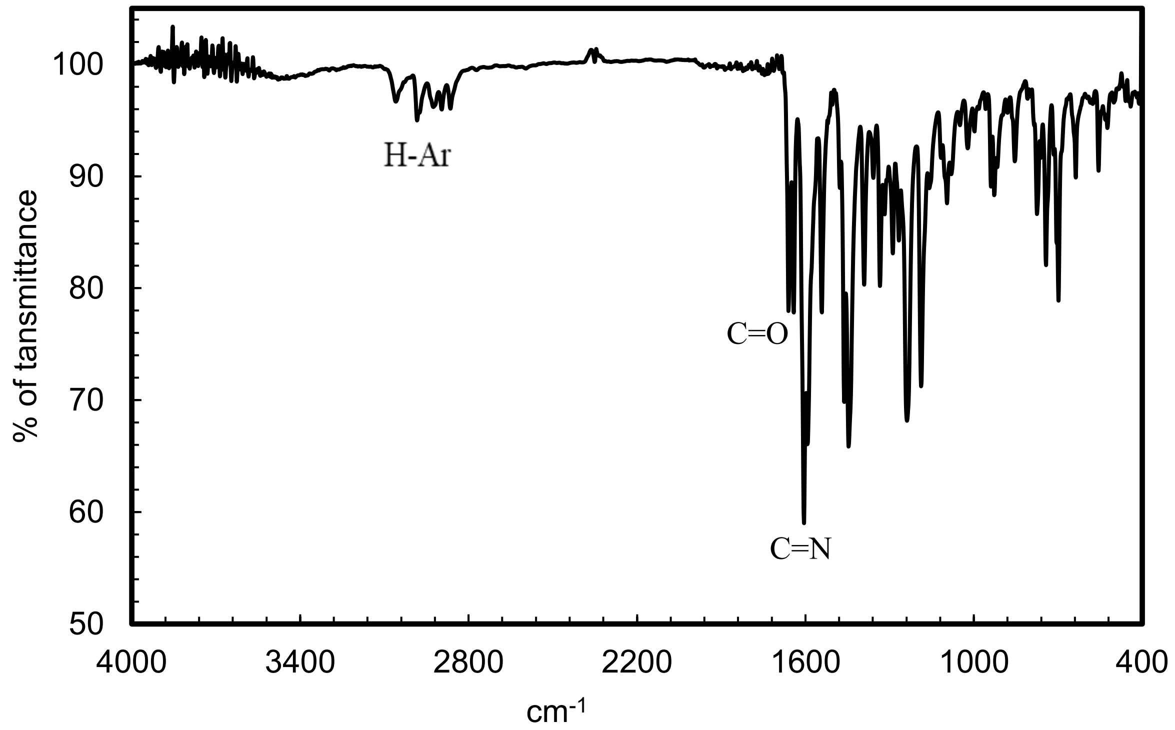

(**2**)

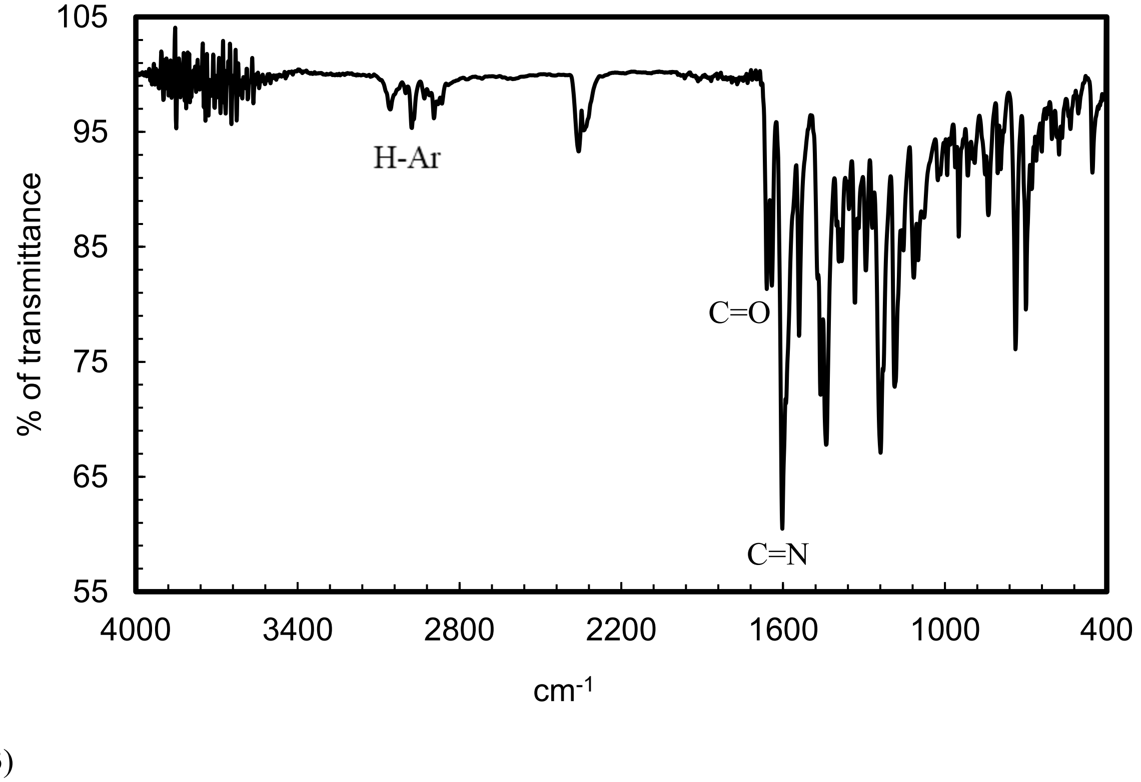

(**3**)

Fig. S1b. IR (KBr, cm^{-1}) spectra for the complexes (**1**-**3**) at ambient temperature.

Section 2: Mass spectra

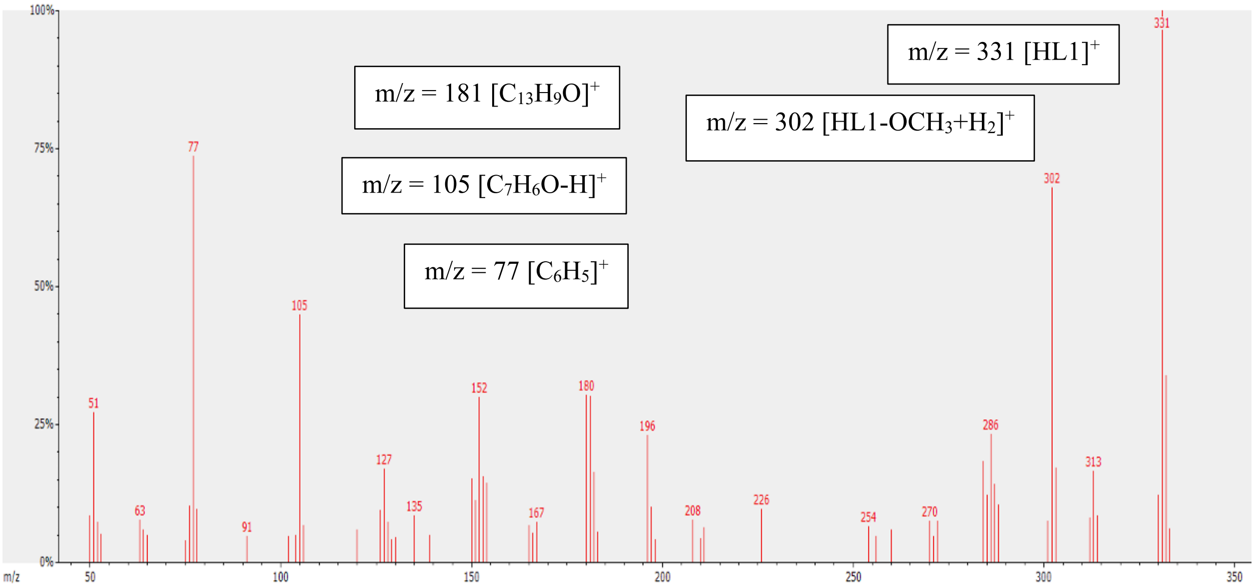

HL1

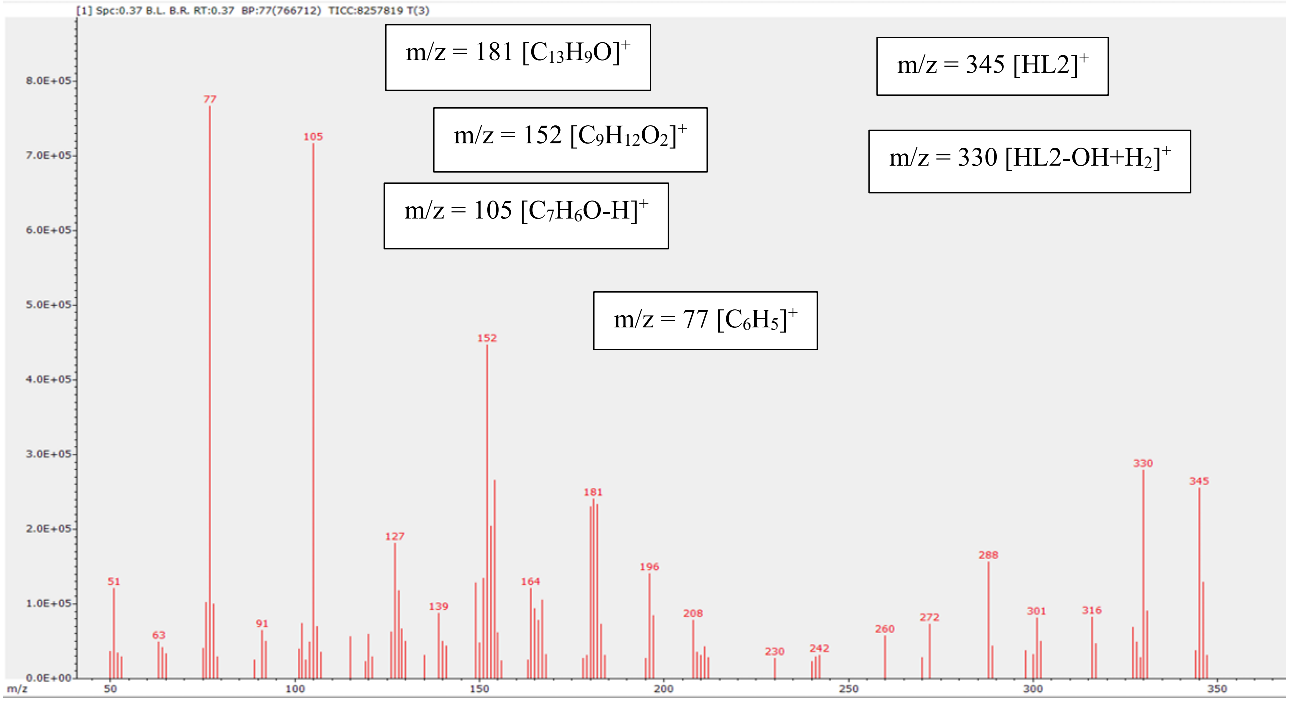

HL2

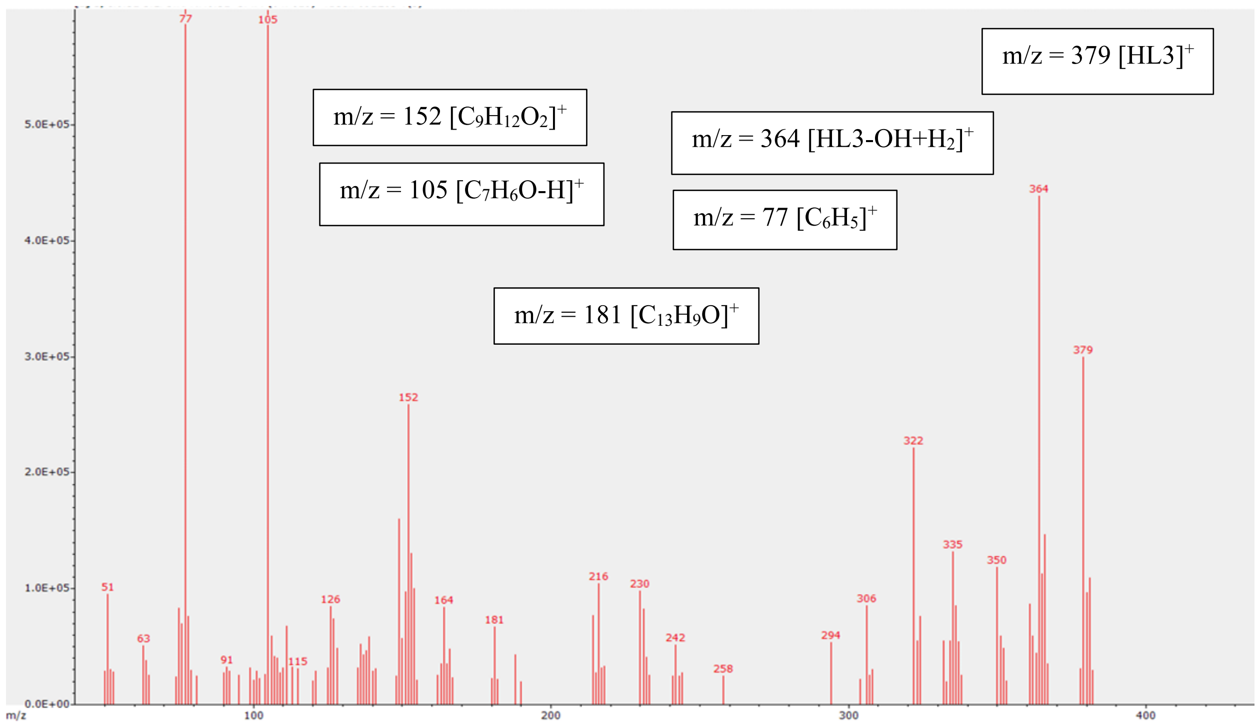

HL3

Fig. S2a. EI-mass spectra for the Schiff base ligands (HL1 – HL3).

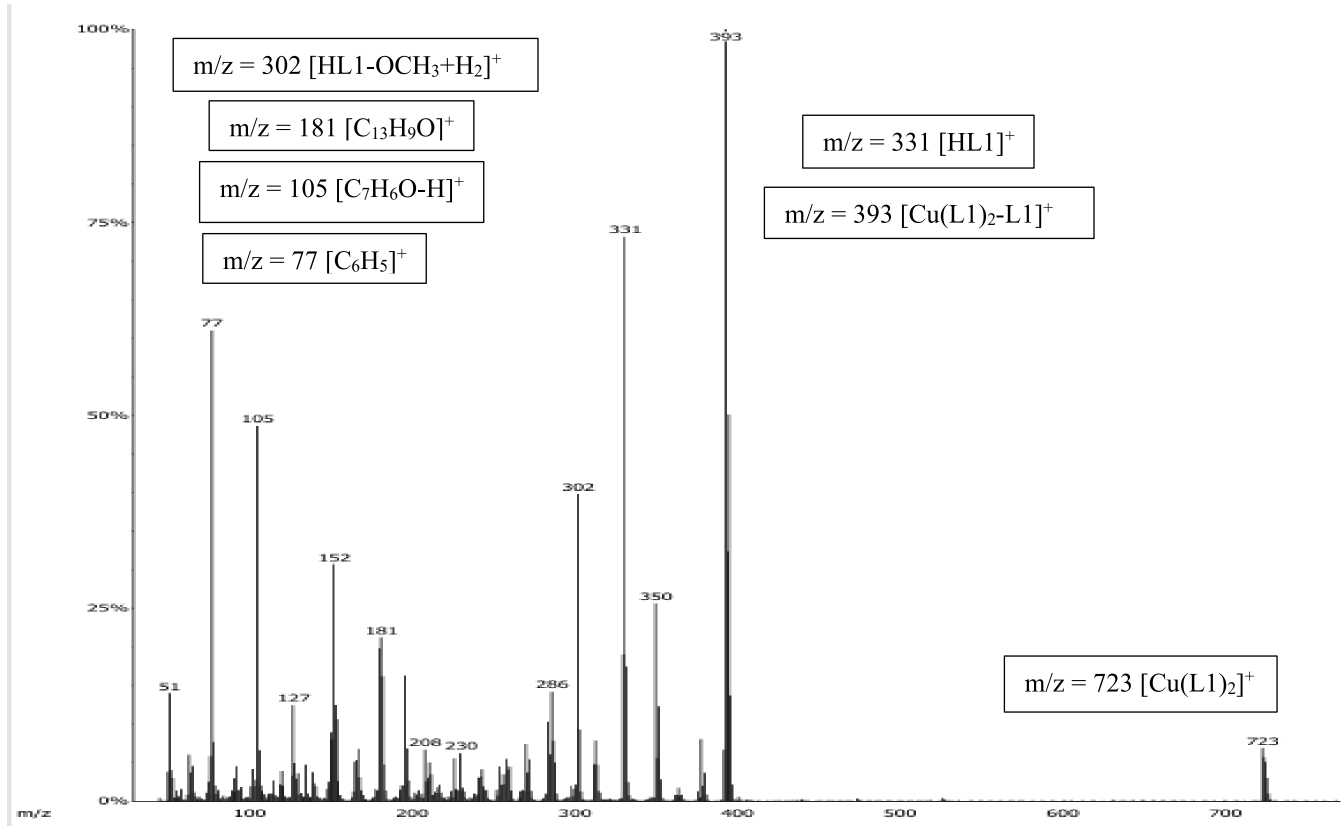

(1)

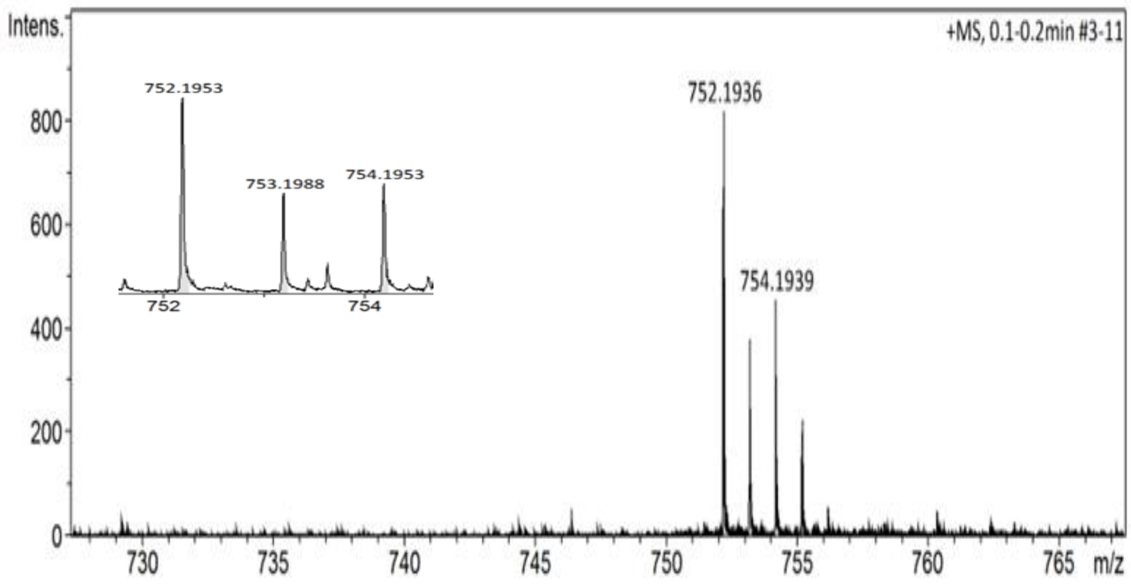

(**3**)

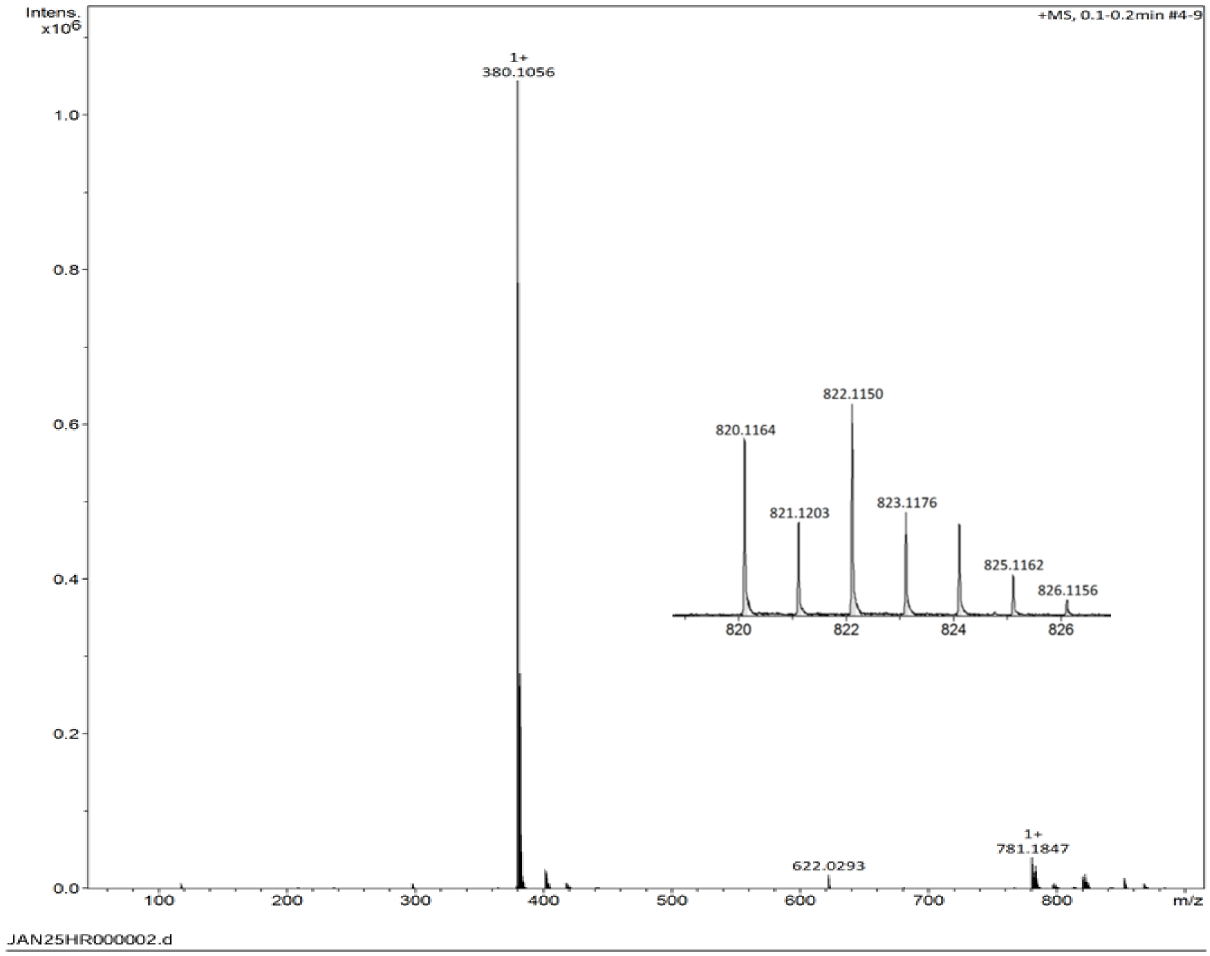

(**3**)

Fig. S2b. EI-mass spectra for **1** and ESI-mass spectra for **2** - **3**.

Section 3: NMR spectra

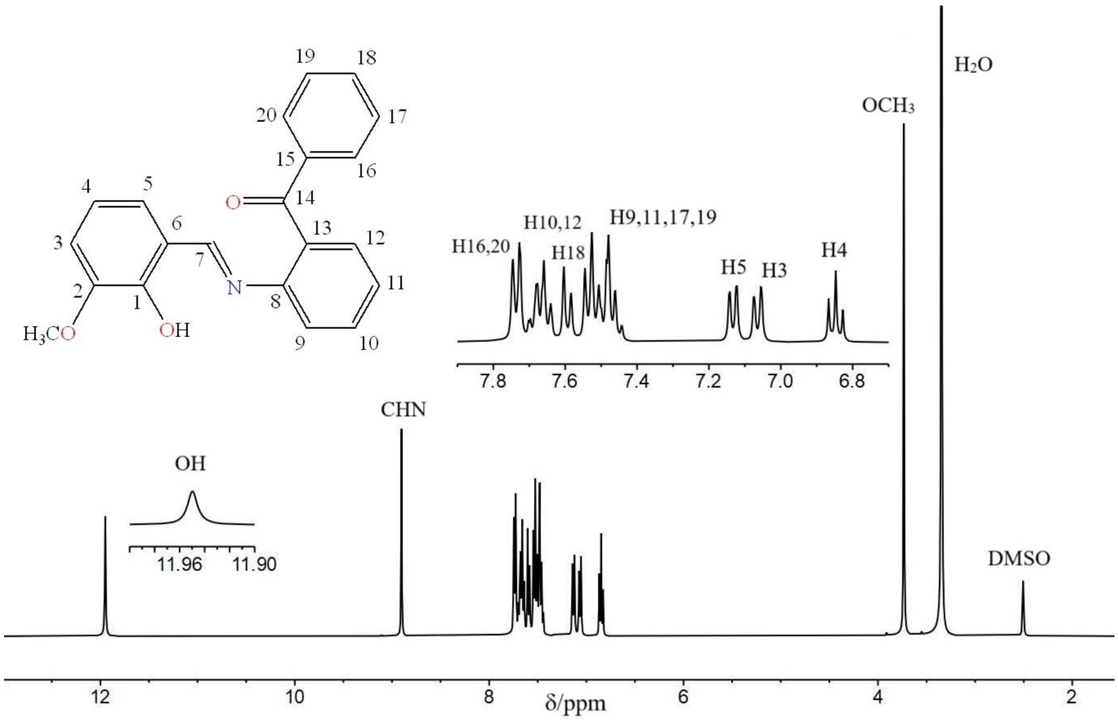

(HL1)

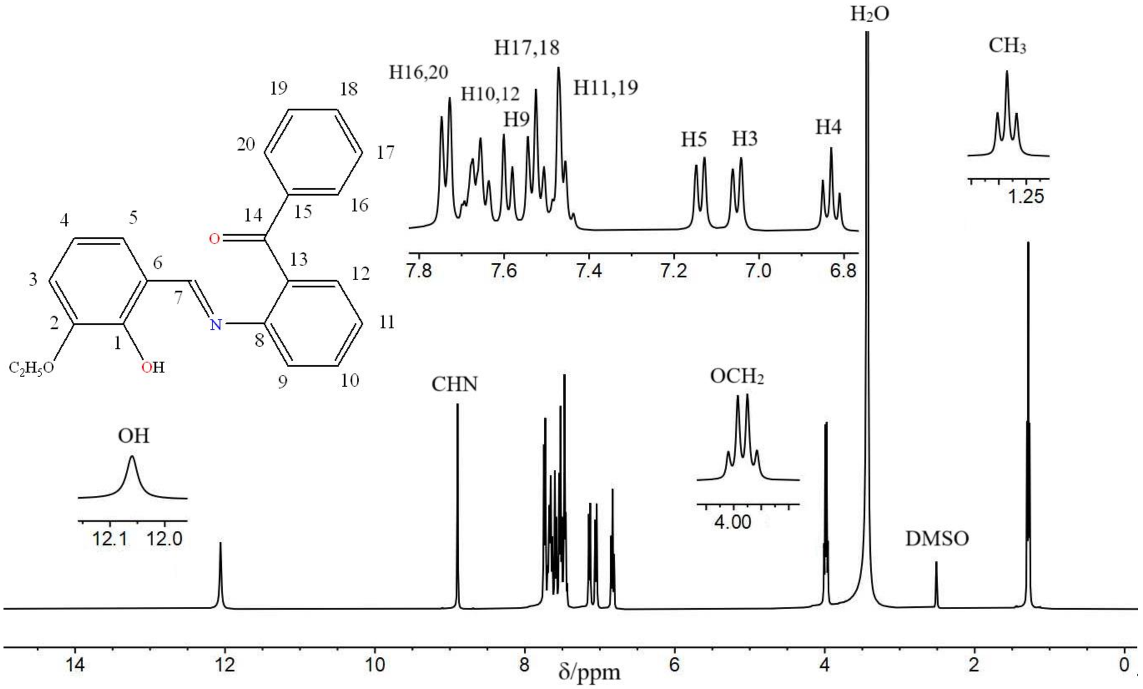

(HL2)

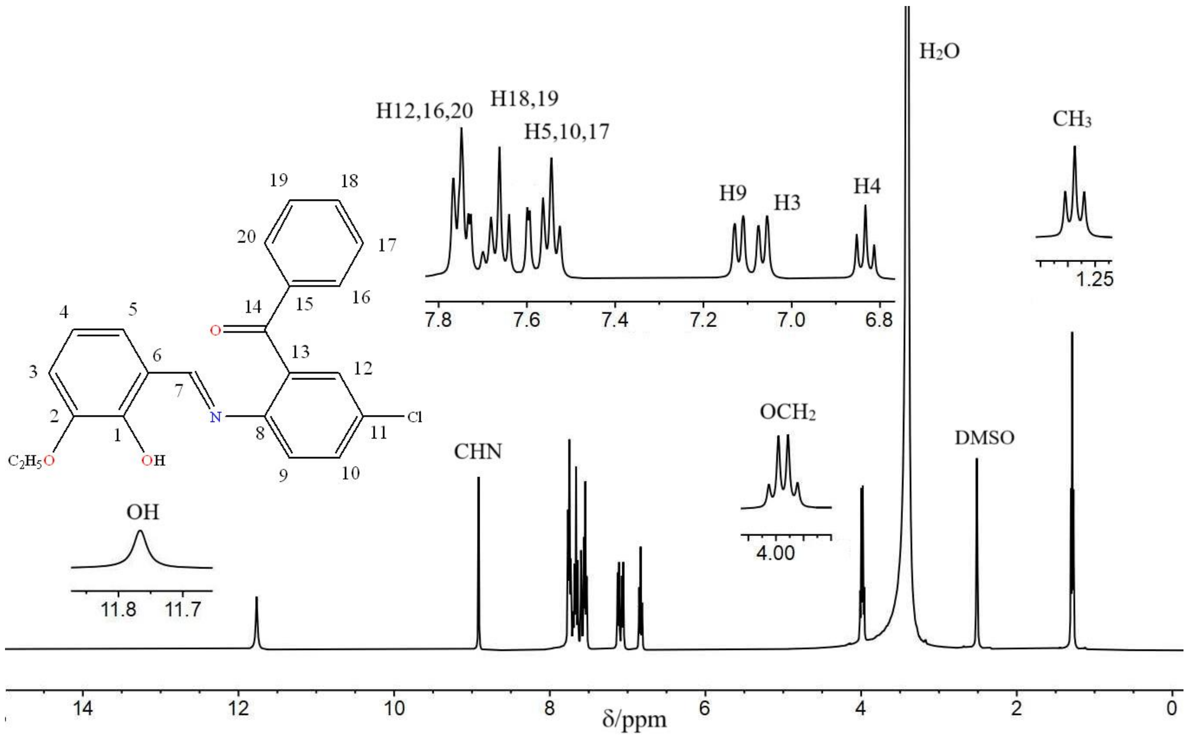

(HL3)

Fig. S3a. ^{1}H NMR (400 MHz) spectra for the Schiff bases HL1, HL2 and HL3 in DMSO-d_6 at 25 °C.

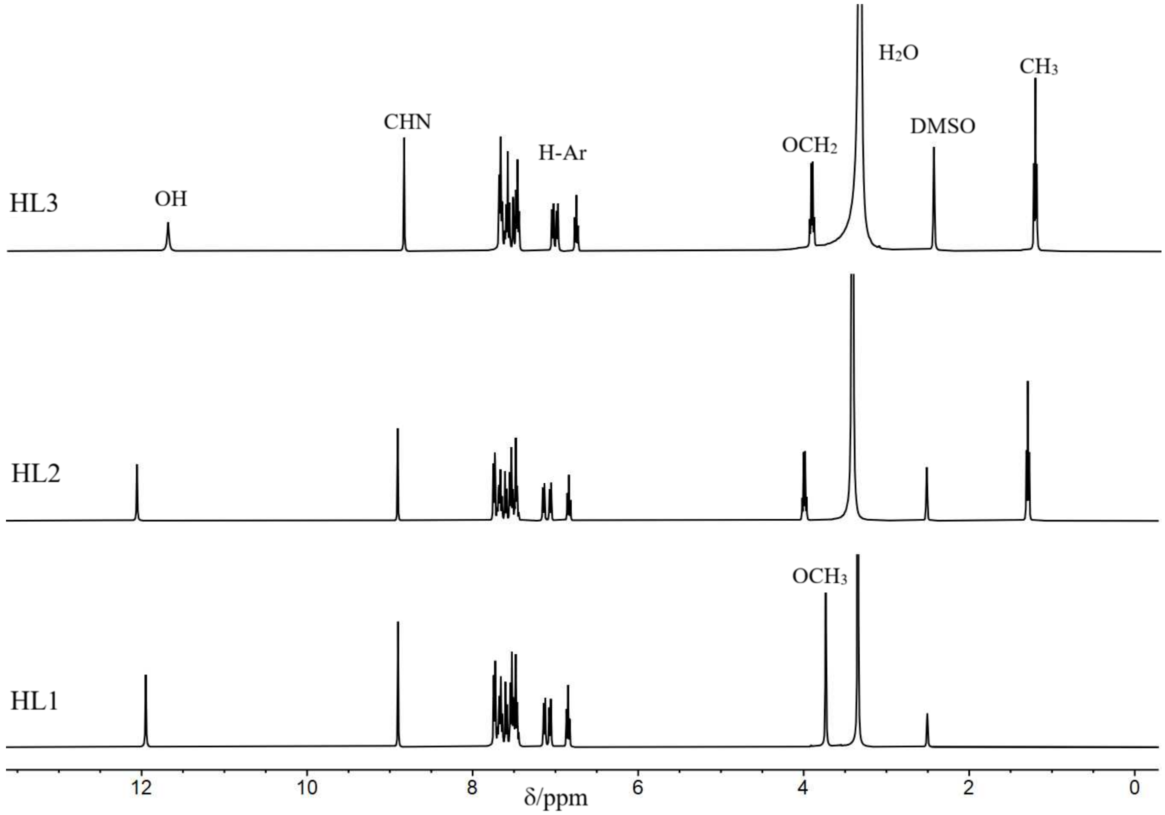

Fig. S3b. ^{1}H NMR spectra of HL1, HL2 and HL3 recorded in DMSO-d_6 at 25 °C, for comparative analysis.

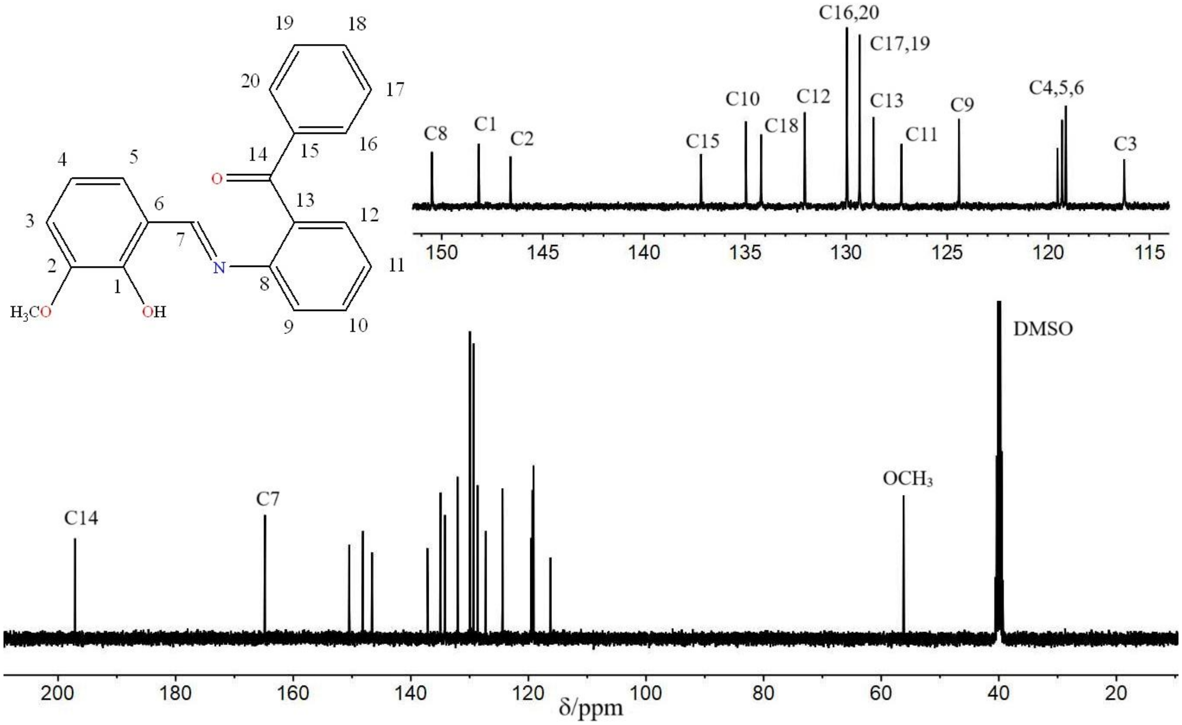
C16,20
C17,19
C10
C12
C13
C9
C4,5,6
C8
C1
C2
C15
C18
C11
C3
DMSO
OCH3
C14
C7
δ/ppm

(HL1)

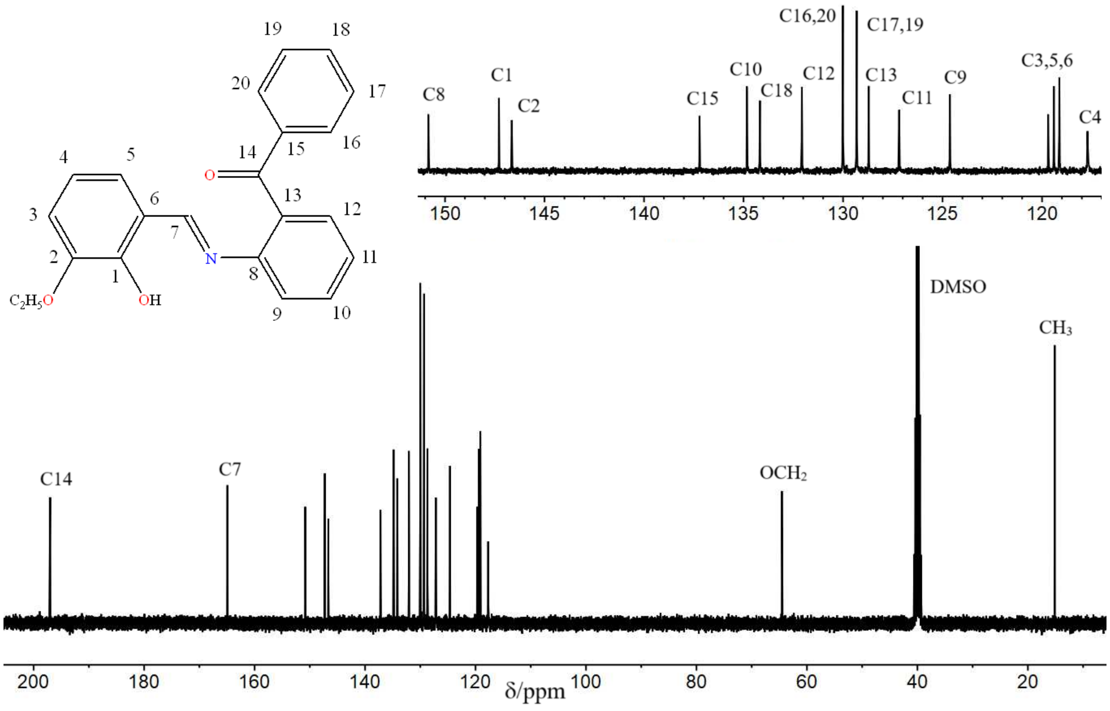
C16,20
C17,19
C10
C12
C13
C9
C3,5,6
C8
C1
C2
C15
C18
C11
C4
DMSO
CH3
C14
C7
OCH2
δ/ppm

(HL2)

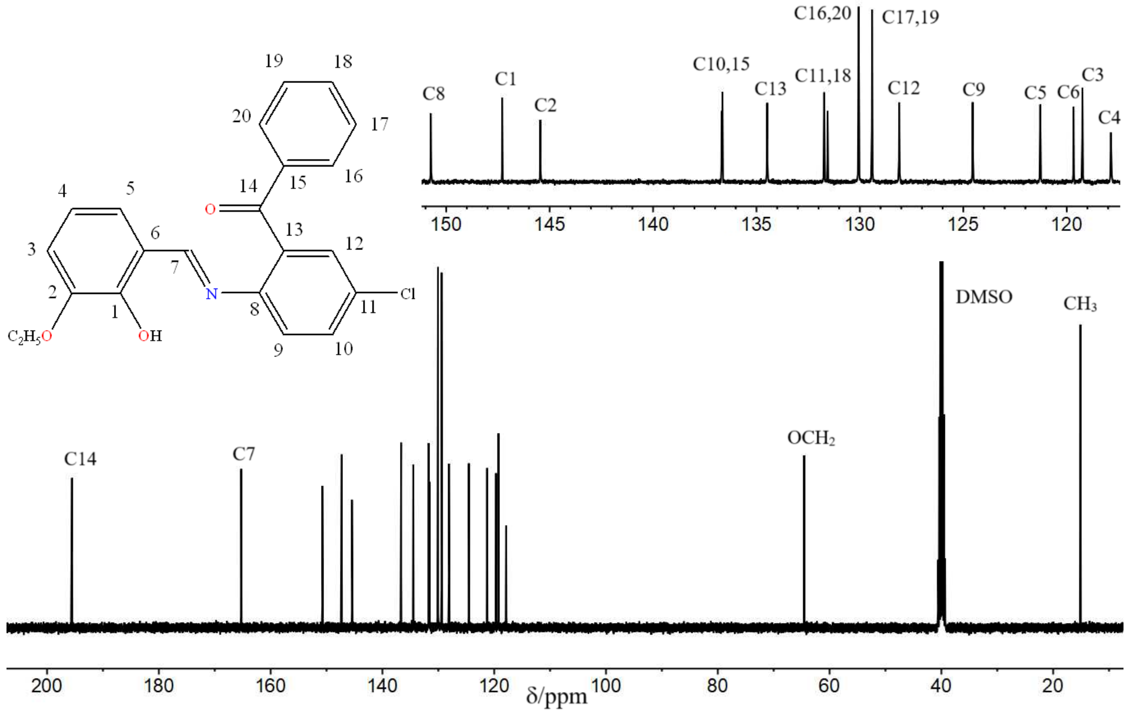

(HL3)

Fig. S4a. ^{13}C NMR (100 MHz) spectra for the Schiff bases HL1, HL2 and HL3 in DMSO-d$_6$ at 25°C.

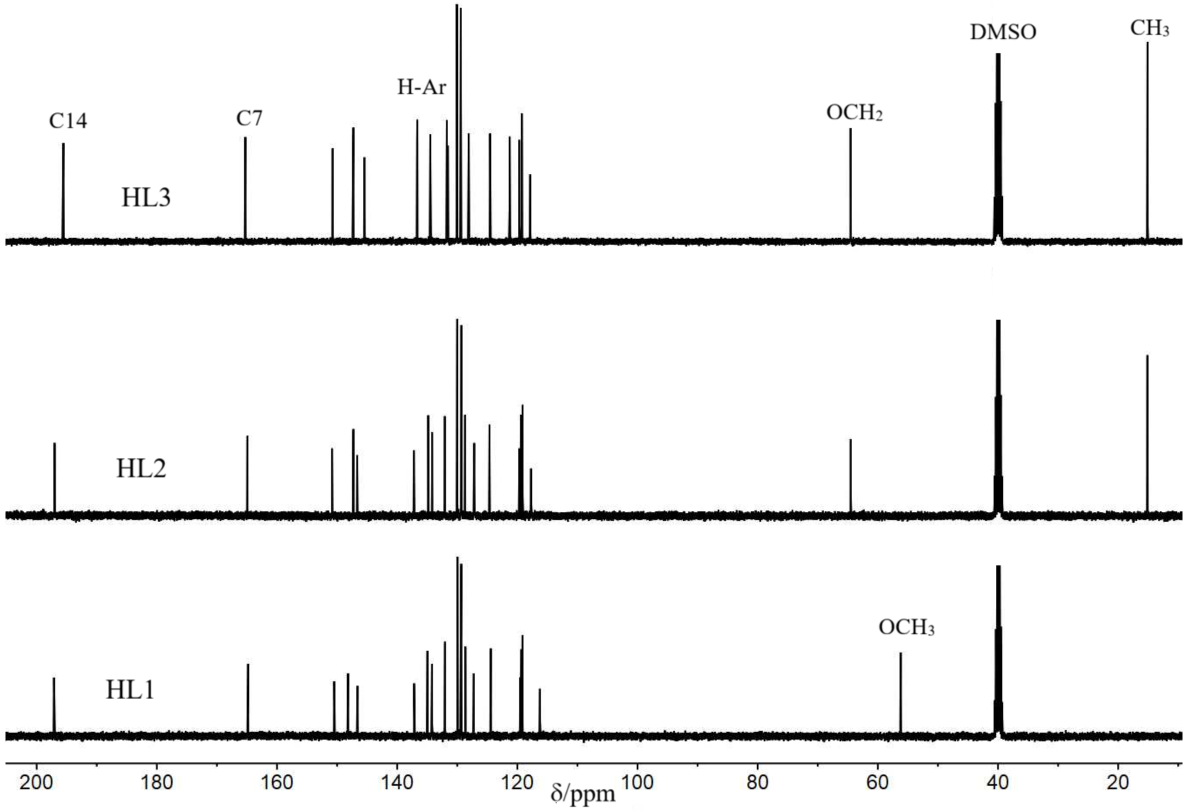

Fig. S4b. ^{13}C NMR spectra of HL1, HL2 and HL3 recorded in DMSO-d$_6$ at 25 °C, for comparative analysis.

Section 4: UV-vis spectra and optimized structures

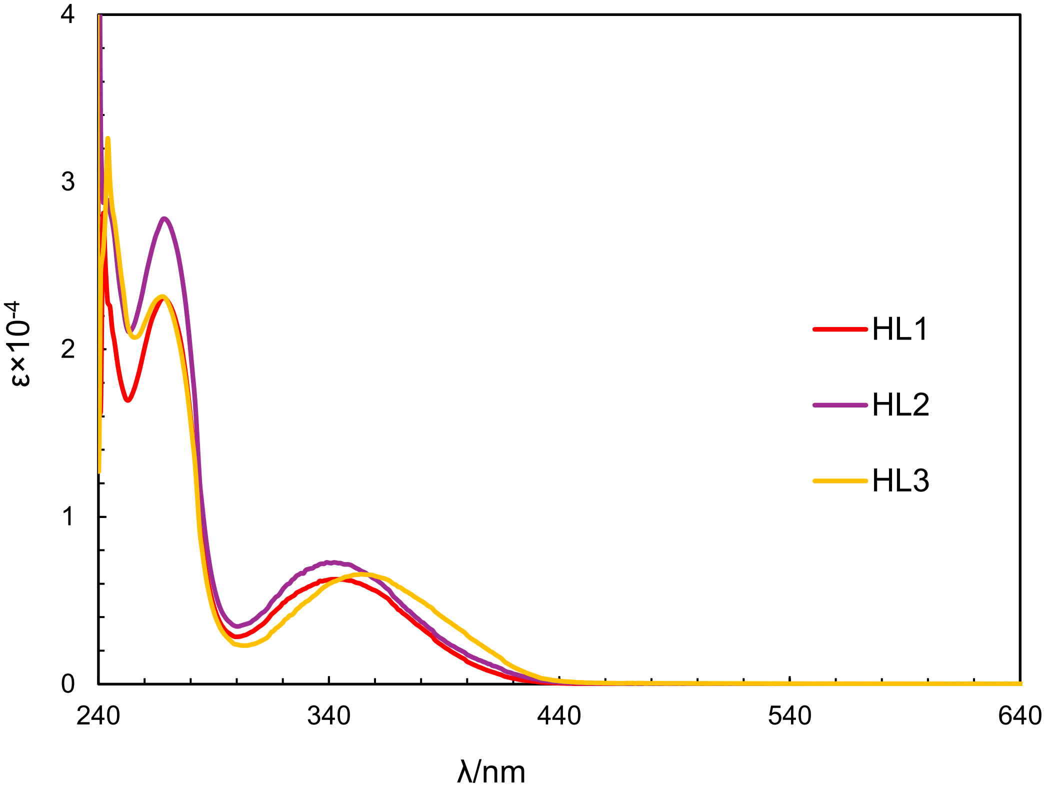

Fig. S5a. UV-vis. spectra for the Schiff bases HL1 (0.043 mM), HL2 (0.021 mM) and HL3 (0.016 mM) in $CHCl_3$ at 25 °C.

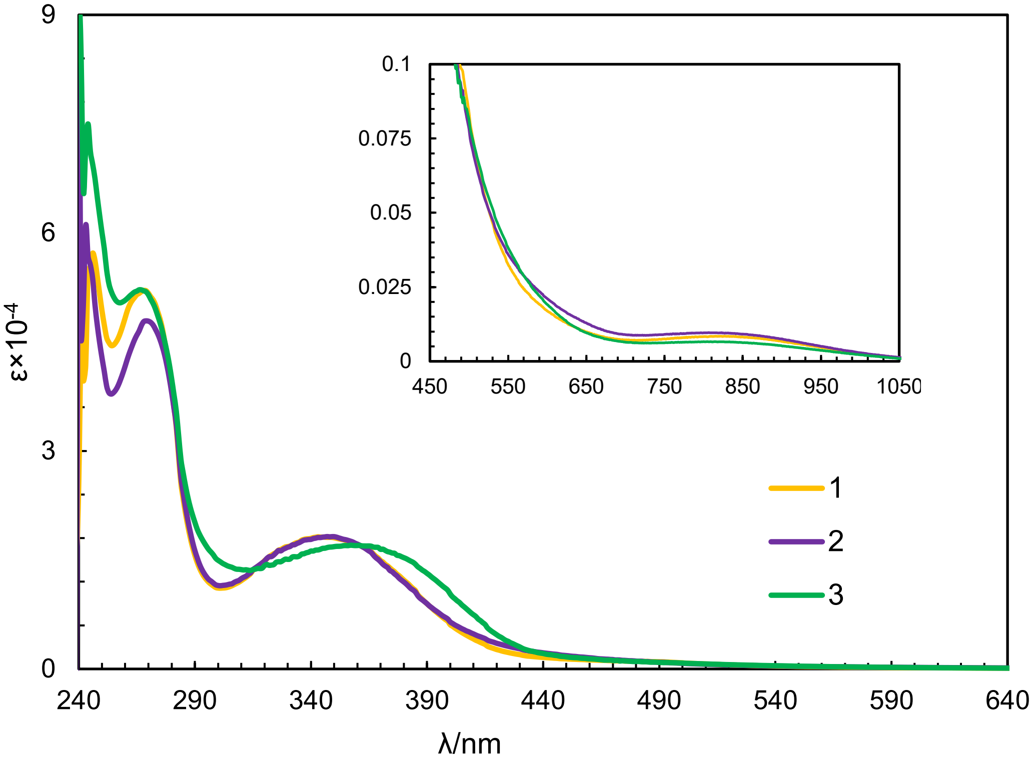

Fig. S5b. UV-vis. spectra for complexes **1** (ca. 0.0292mM), **2** (ca. 0.0175 mM) and **3** (ca. 0.0104 mM) in $CHCl_3$ at 25 °C (spectra in visible range are shown in the inset).

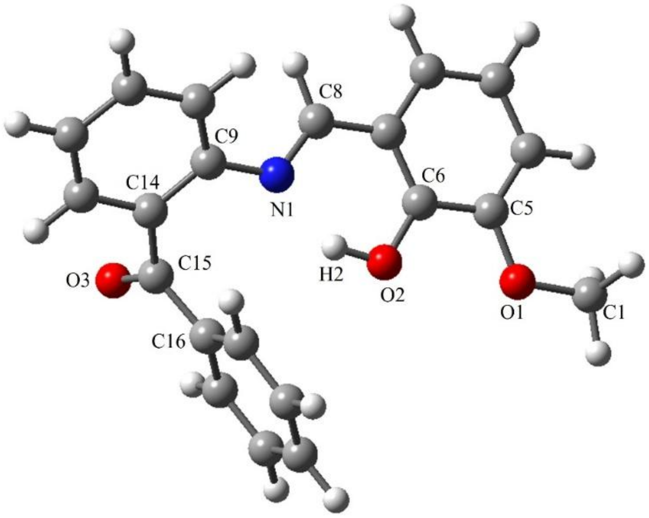

(a) HL1 at B3LYP/6-31G(d)

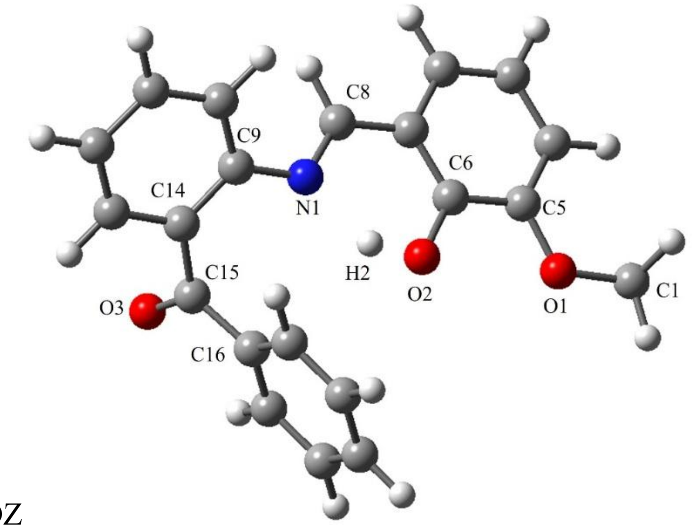

(b) HL1 at B3LYP/ LANL2DZ

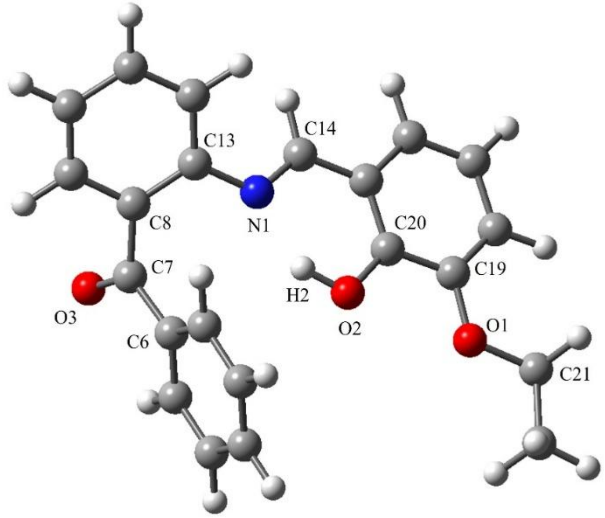

(c) HL2 at B3LYP/6-31G(d)

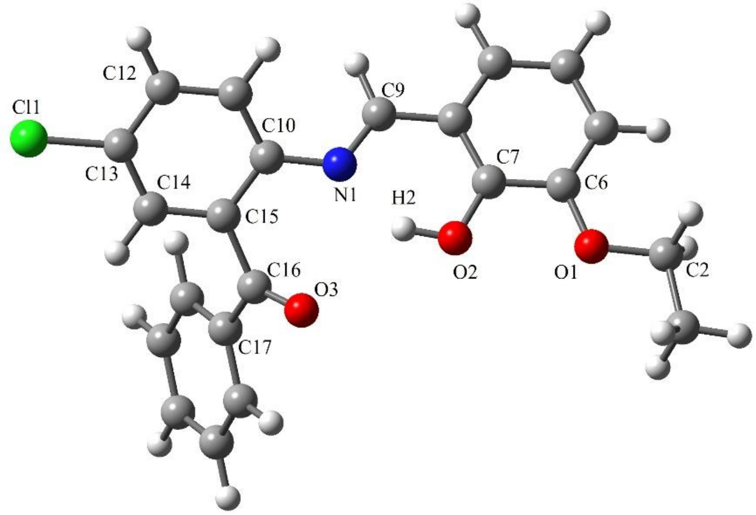

(d) HL3 at B3LYP/6-31G(d)

Fig. S6a. Optimized structures for (a) HL1 at B3LYP/6-31G(d), (b) HL1 at B3LYP/ LANL2DZ, (c) HL2 at B3LYP/6-31G(d) and (d) HL3 at B3LYP/6-31G(d).

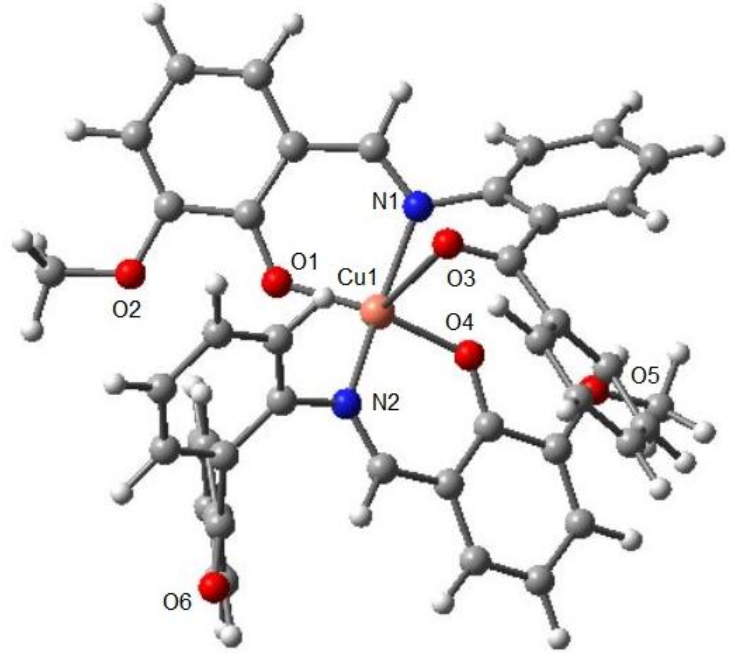

(**1**)

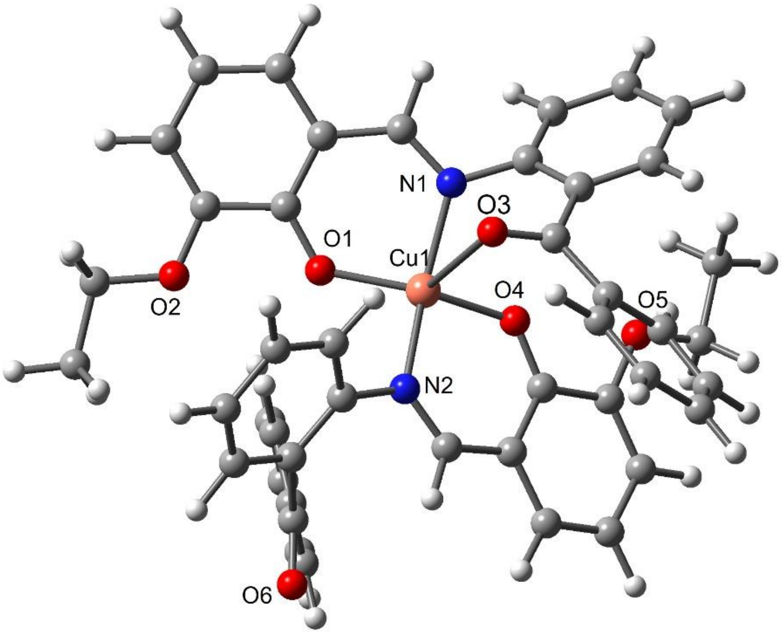

(**2**)

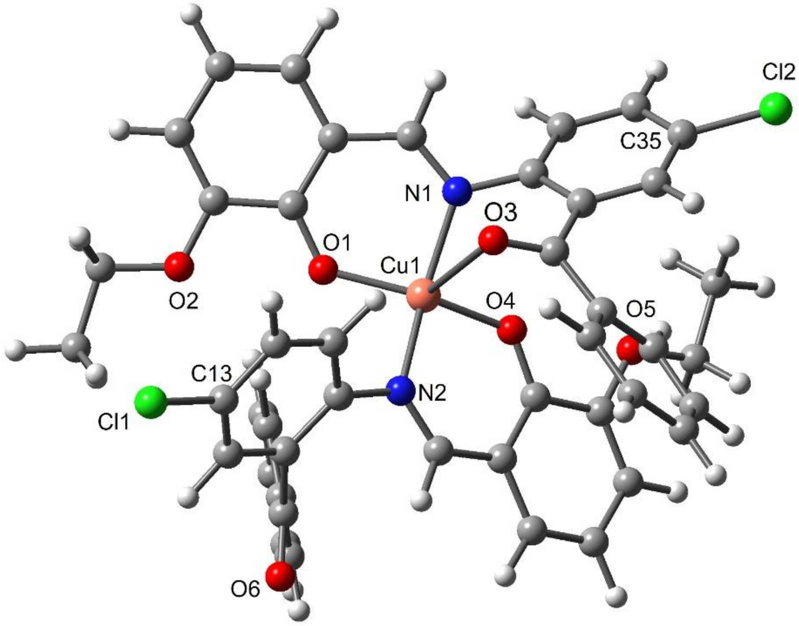

(**3**)

Fig. S6b. Optimized structures for **1**, **2** and **3** calculated with B3LYP/6-31G(d).

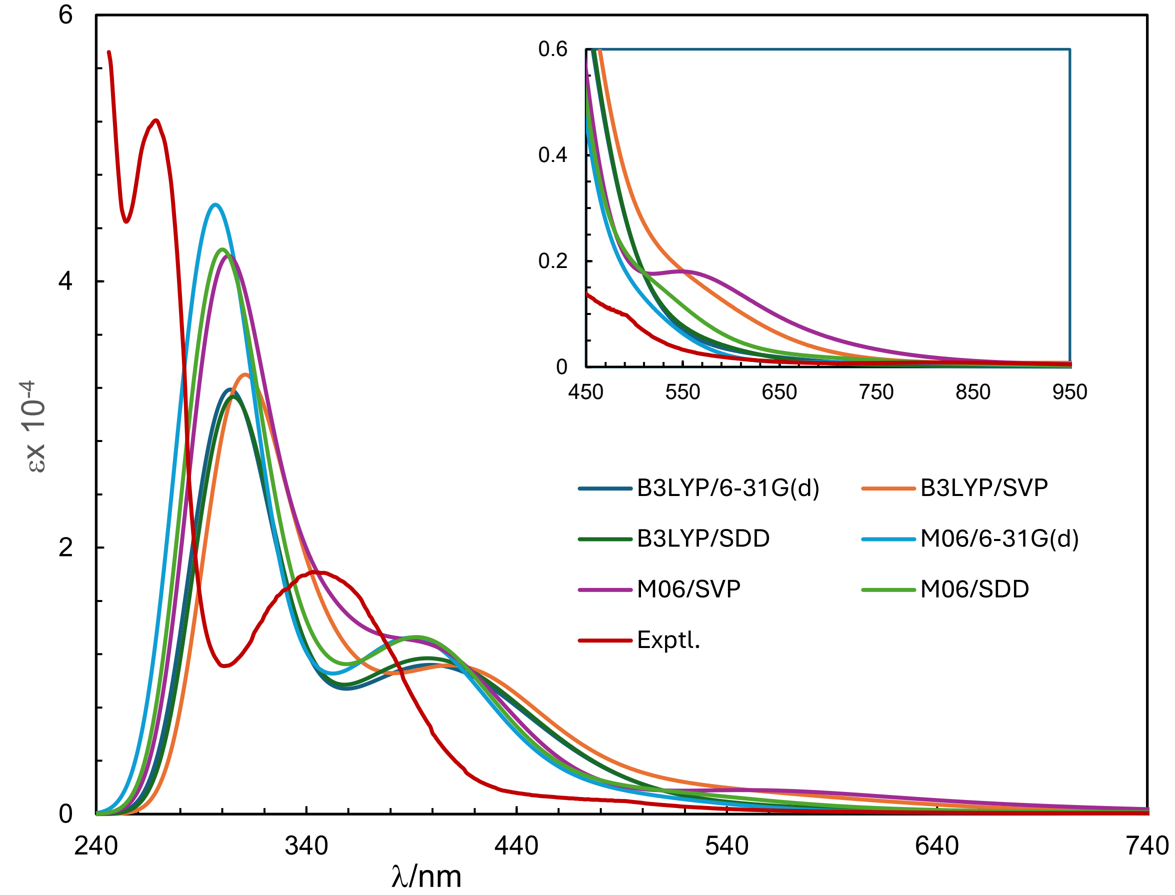

Fig. S7. Experimental and simulated UV-vis. spectra for complex **1** (ca. 0.0292mM) in $CHCl_3$ (d-d bands in visible range are shown in the inset). Spectra were calculated with different combinations of the functions and the basis sets with PCM in $CHCl_3$. Gaussian band shape with exponential half-width, σ = 0.33 eV.

Section 5: Intermolecular interactions

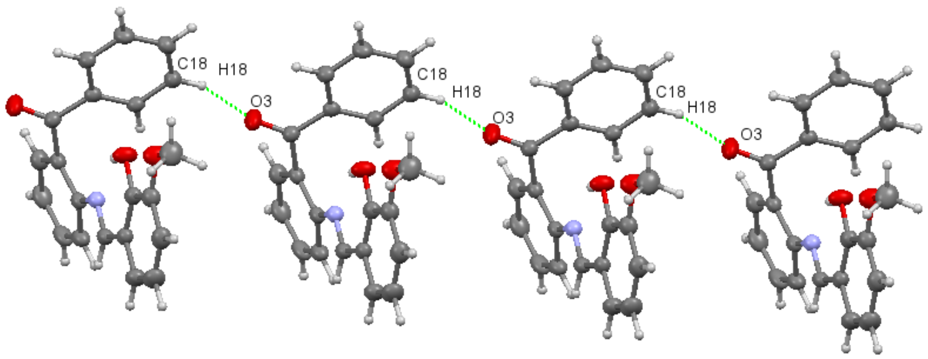

(HL1)

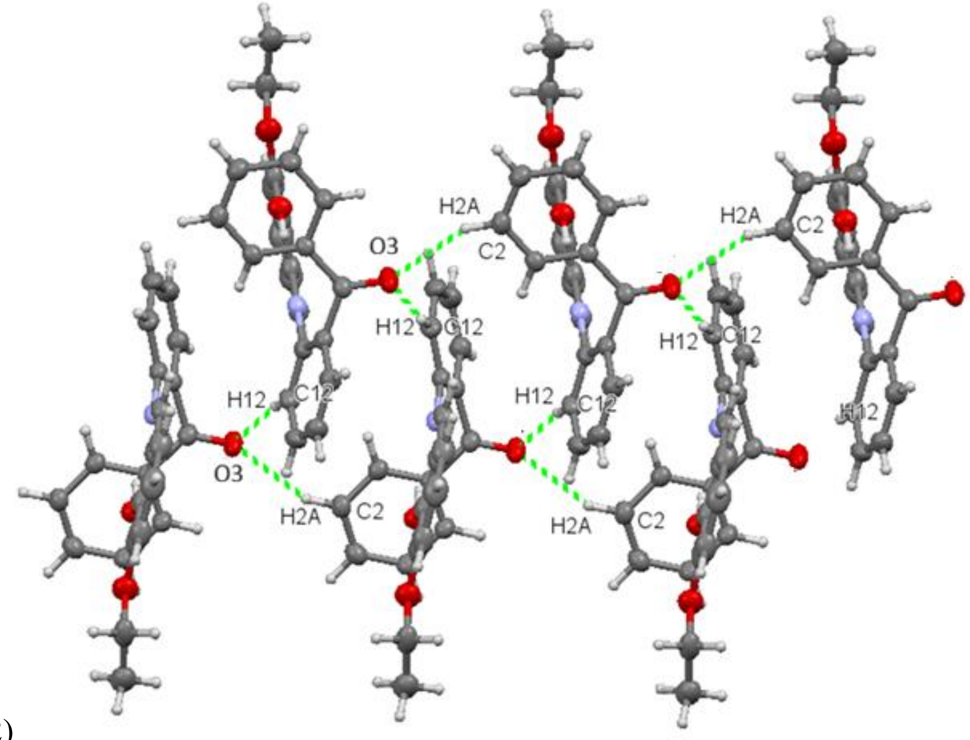

(HL2)

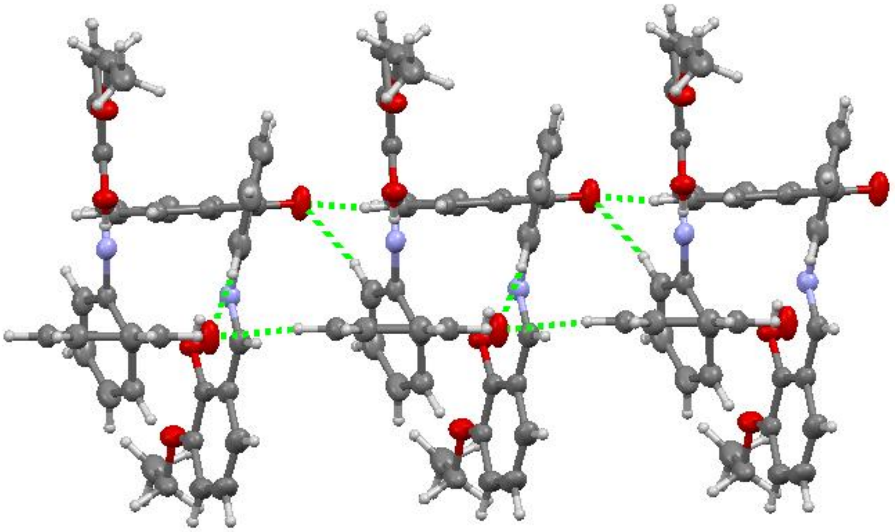

(HL2)

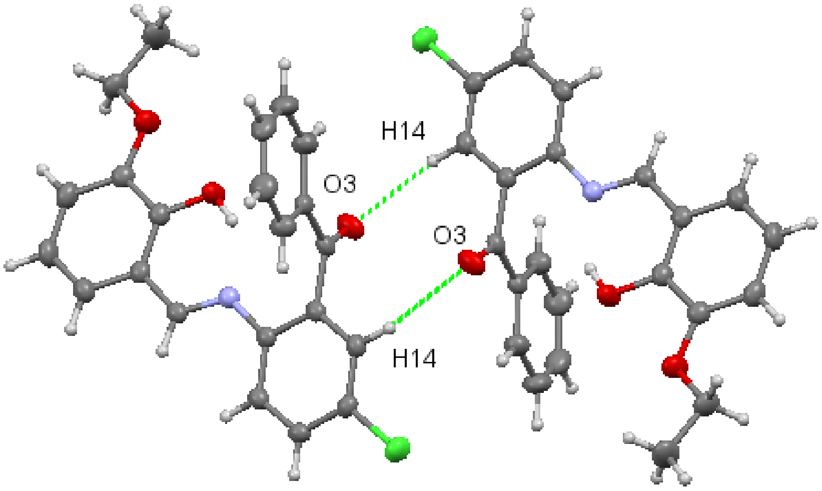

(HL3)

Fig. S8. Illustration of intermolecular C–H····O contacts (dashed green line) among the symmetry related molecules in HL1, HL2 and HL3 (50% thermal ellipsoids, H atoms with arbitrary radii). Symmetry transformations, i = -1+x, y, z (HL1); x, -1+y, z and 3/2-x, -1/2+y, 1/2-z (HL2); 1-x, 1-y, 1-z (HL3).

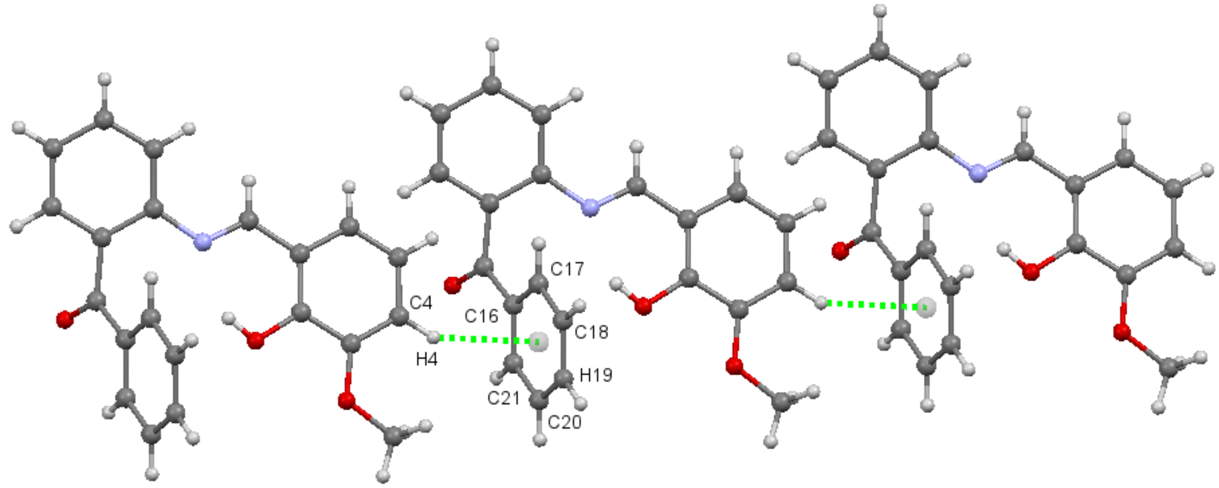

(HL1)

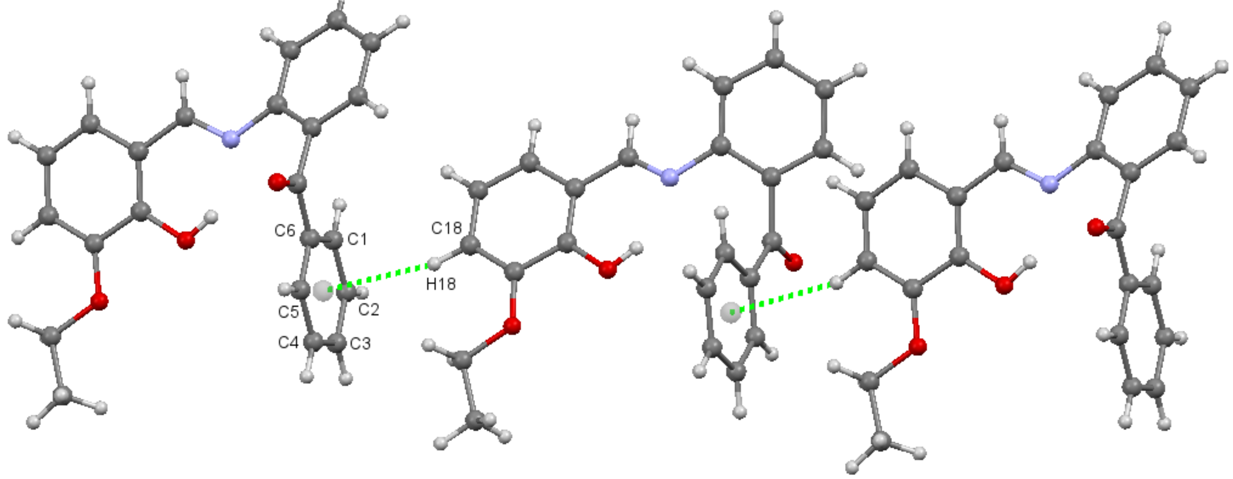

(HL2)

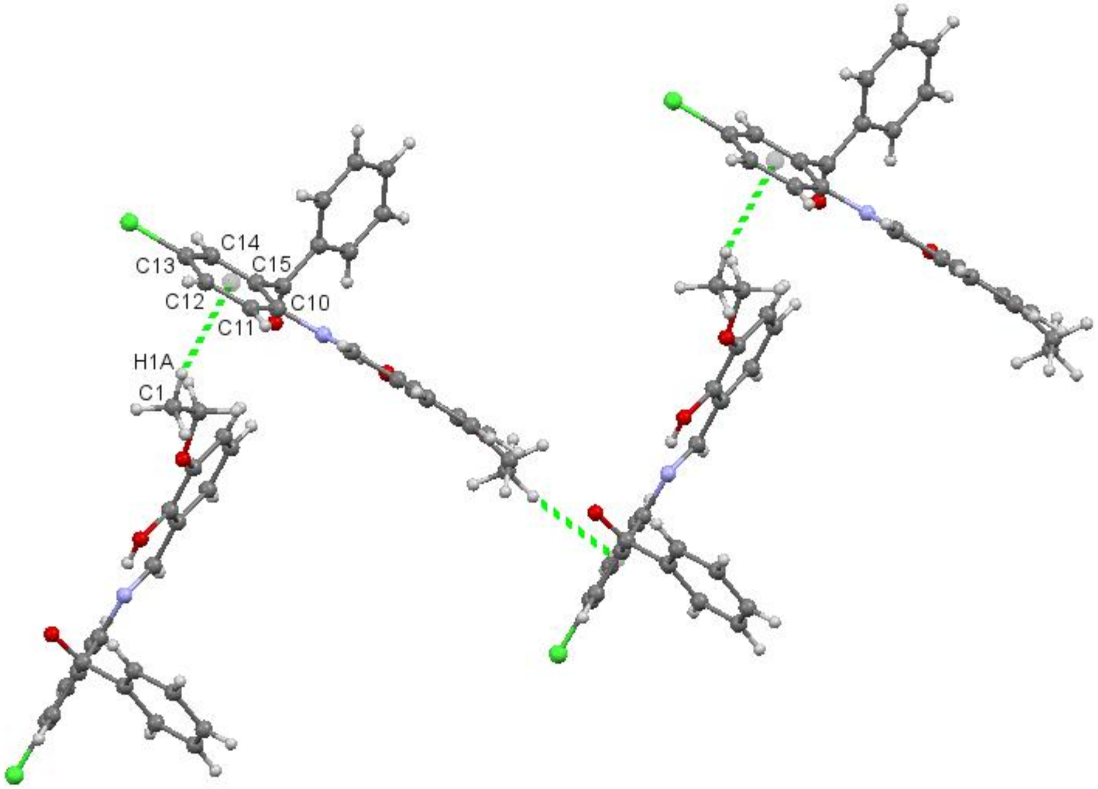

(HL3)

Fig. S9. Illustration of intermolecular C–H····π contacts (dashed green line) with C4–H4···Cg = 2.84 Å (146º) (Cg represents the centroid of the C16-C21 ring) in HL1, C18–H18···Cg = 2.80 Å (162º) (Cg represents the centroid of the C1-C6 ring) in HL2 and C1–H(1A)···Cg = 2.75 Å (158º) (Cg represents the centroid of the C10-C15 ring) in HL3.

Symmetry transformations, i = x, 1+y, z (HL1); -1/2+x, 1/2-y, -1/2+z (HL2); 3/2-x, 1/2+y, 1/2-z (HL3).

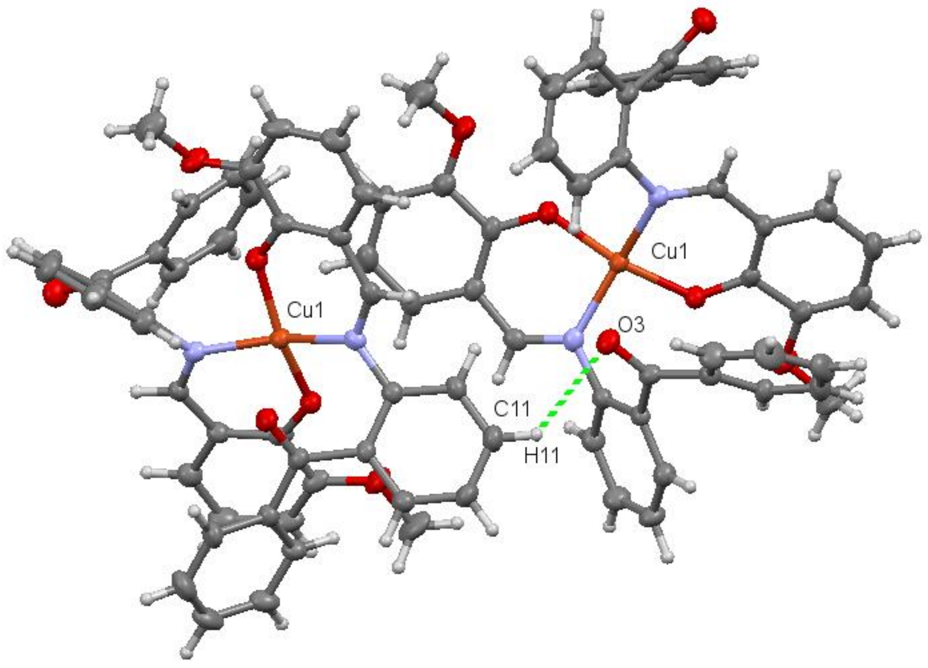

(1)

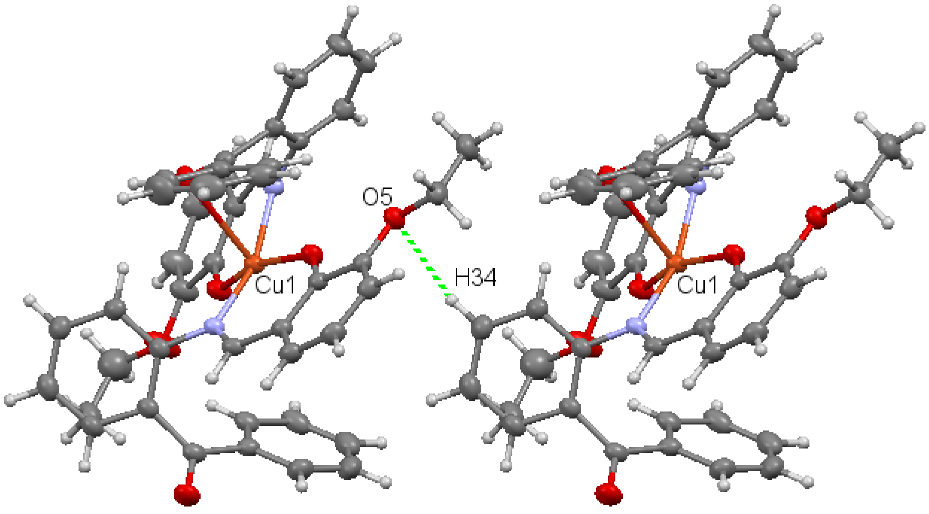

(2)

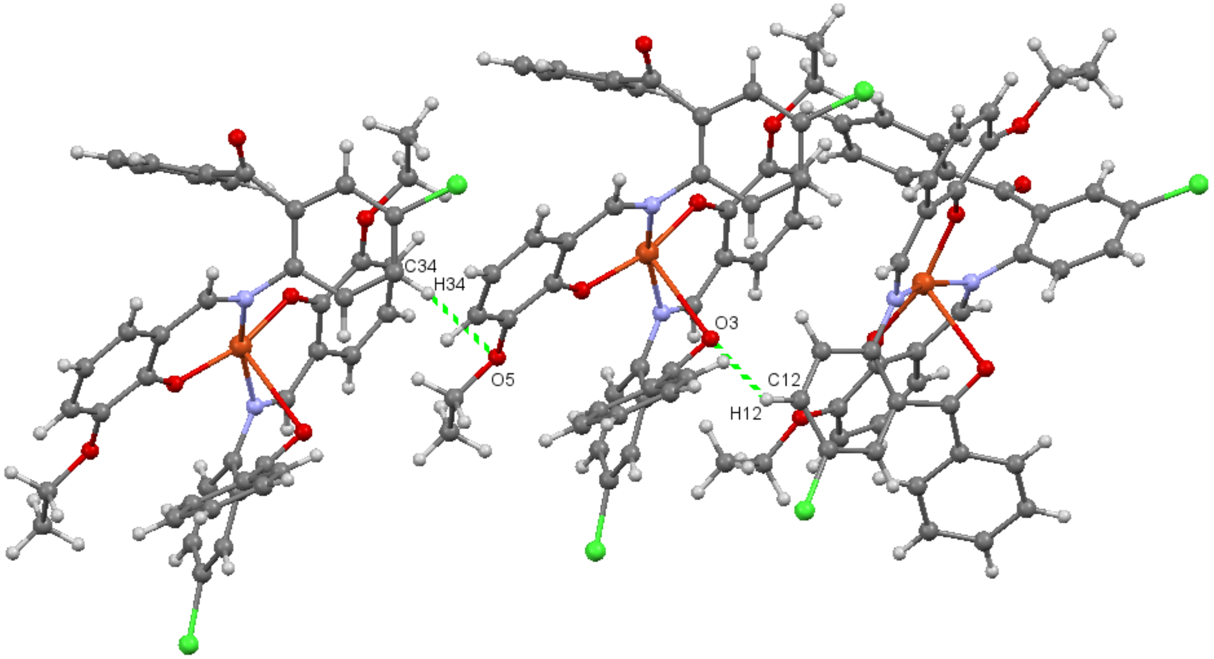

(3)

Fig. S10. Illustration of intermolecular C–H····O contacts (dashed green line) among symmetry related molecules in **1**, **2** and **3** (50% thermal ellipsoids, H atoms with arbitrary radii).

Symmetry transformations, i = x, 1-y, -1/2+z (**1**); x, y, 1+z (**2**); x, y, -1+z and x, -y, 1/2+z (**3**)

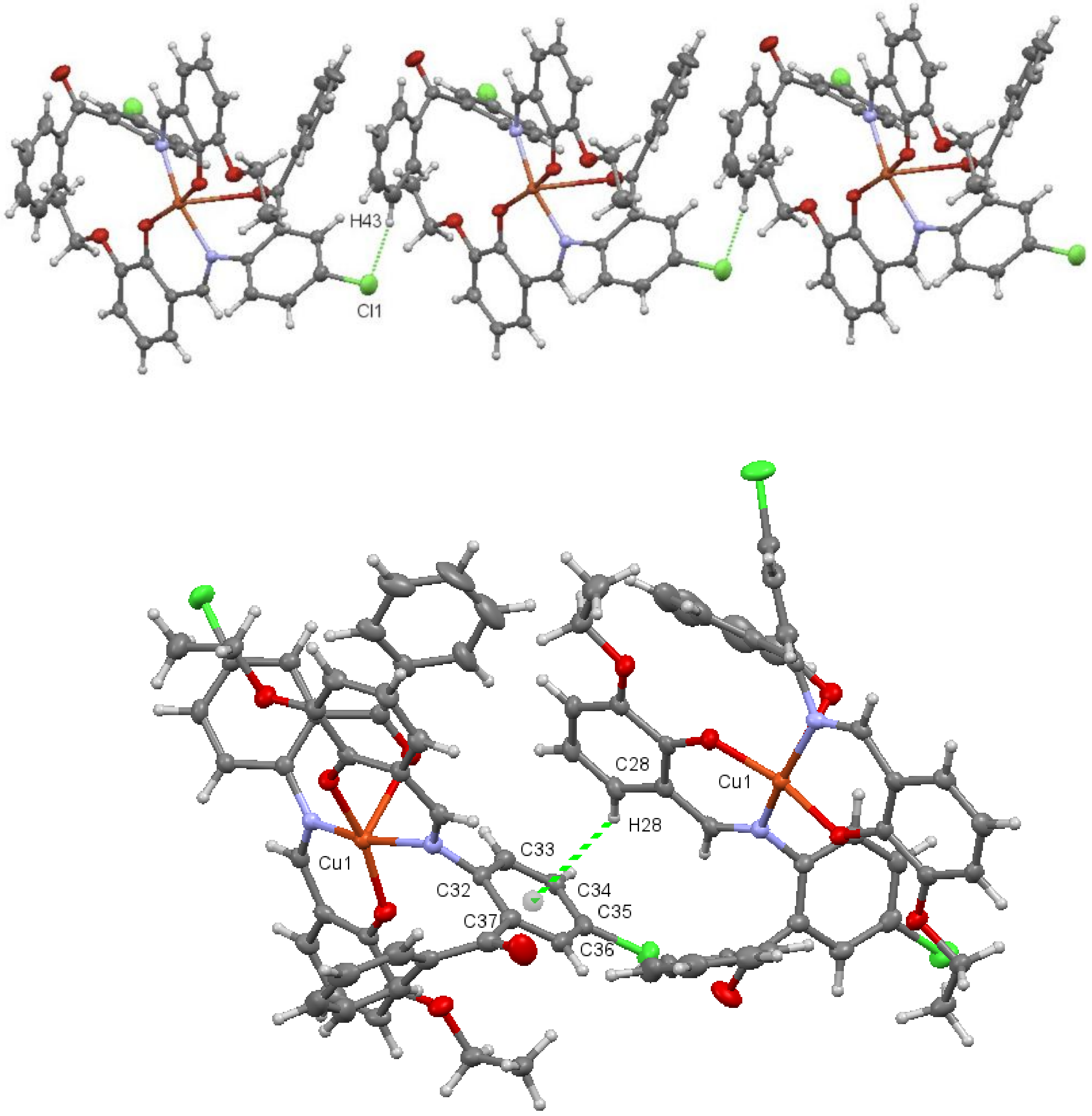

Fig. S11. Illustration of intermolecular C–H···Cl and C–H····π contacts (dashed green line) with C43–H43···Cl1 = 2.86 Å (C–H···Cl 149º, H···Cl–C 112°) and C28–H28···Cg = 2.82 Å (136º) (Cg represents the centroid of C32-C37) in **3**, respectively (50% thermal ellipsoids, H atoms with arbitrary radii). Symmetry transformation, i = x, -1-y, 1/2+z.

Sections 6: Hirshfeld surfaces

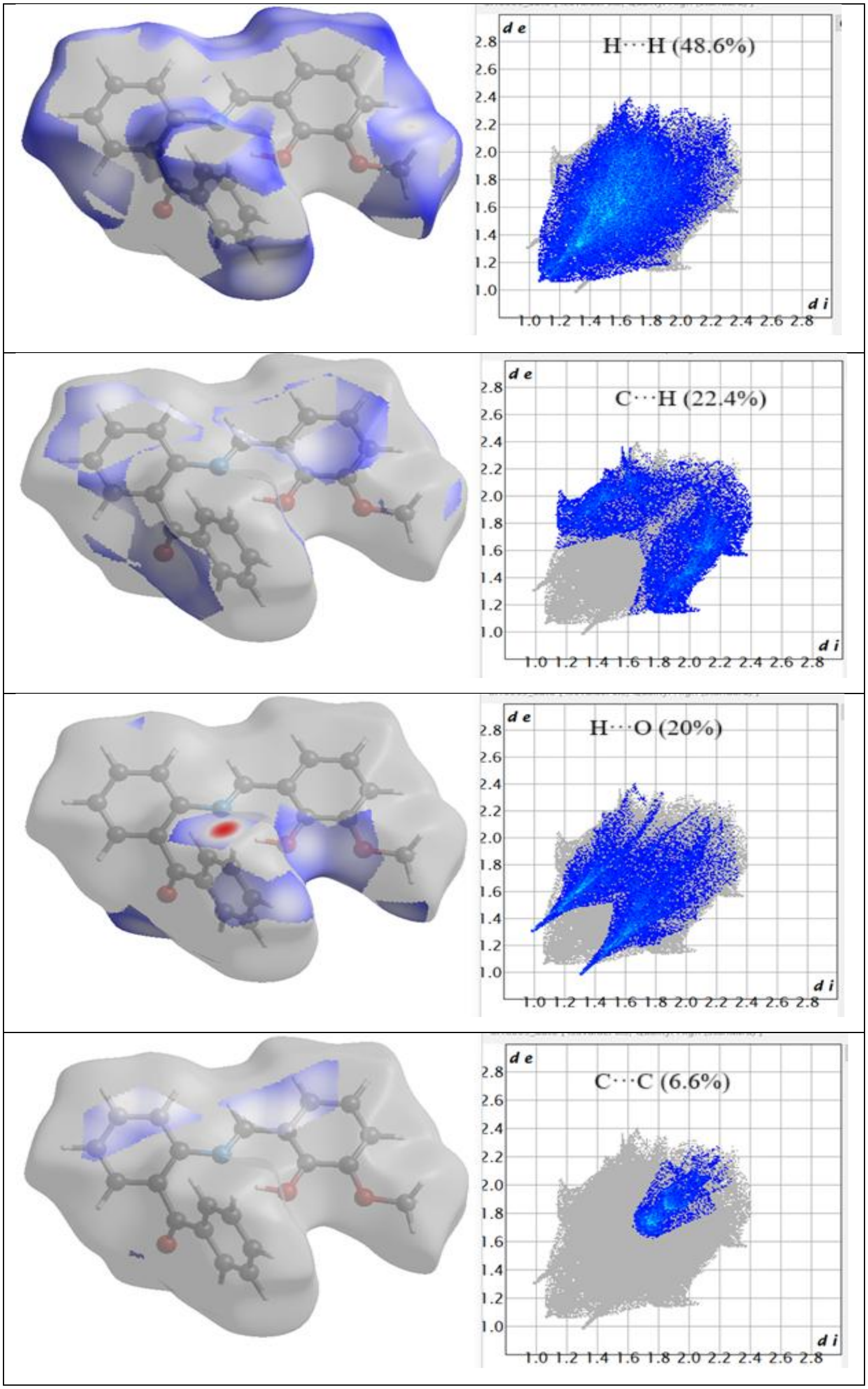

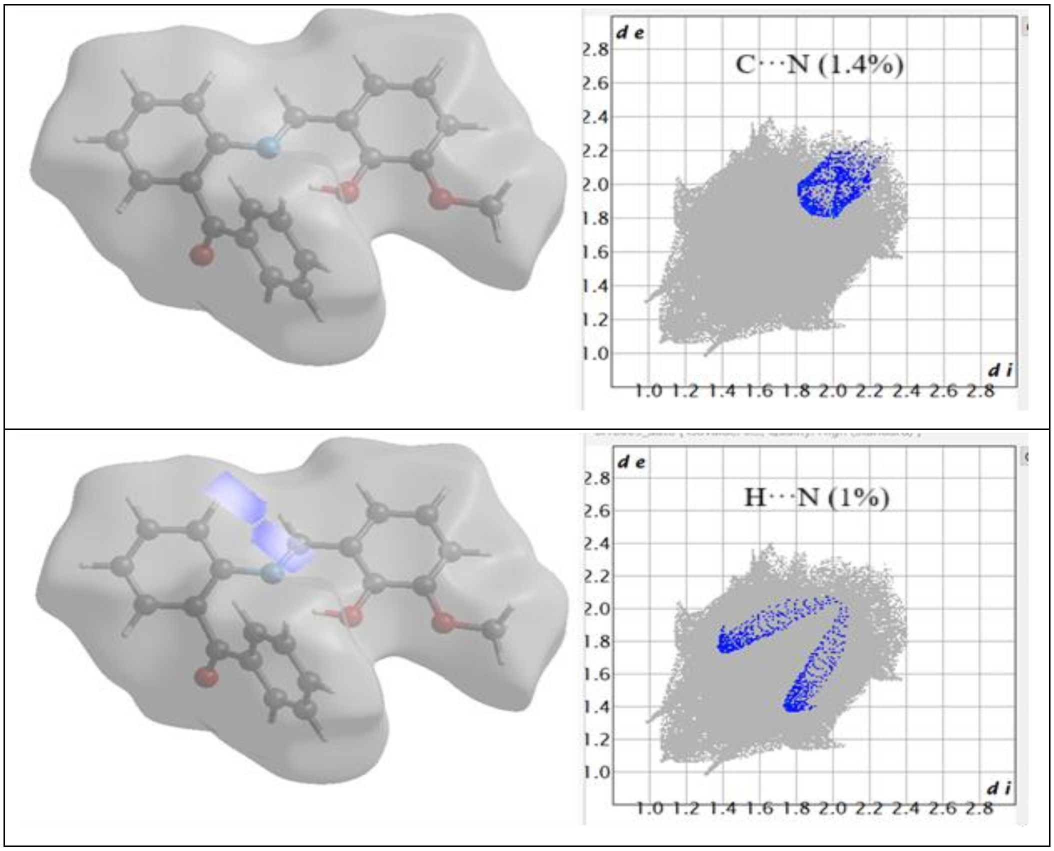

Fig. S12a. Breakdown of all possible contacts to Hirshfeld surfaces for HL1.

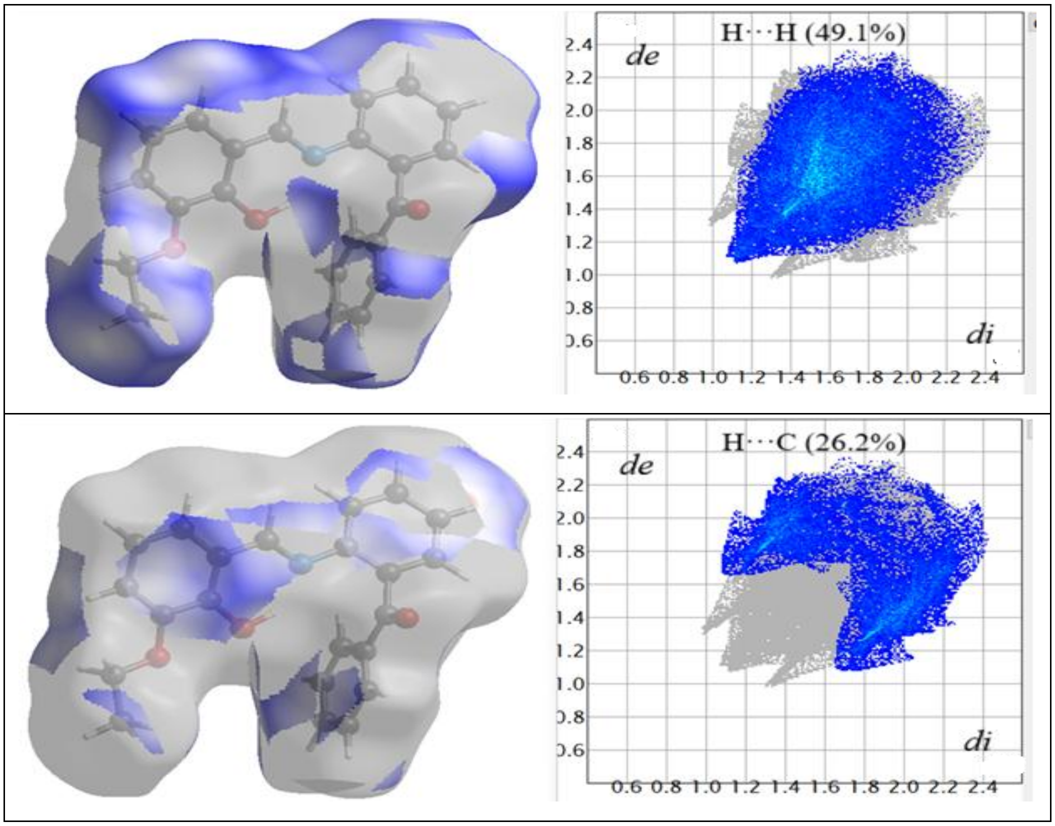

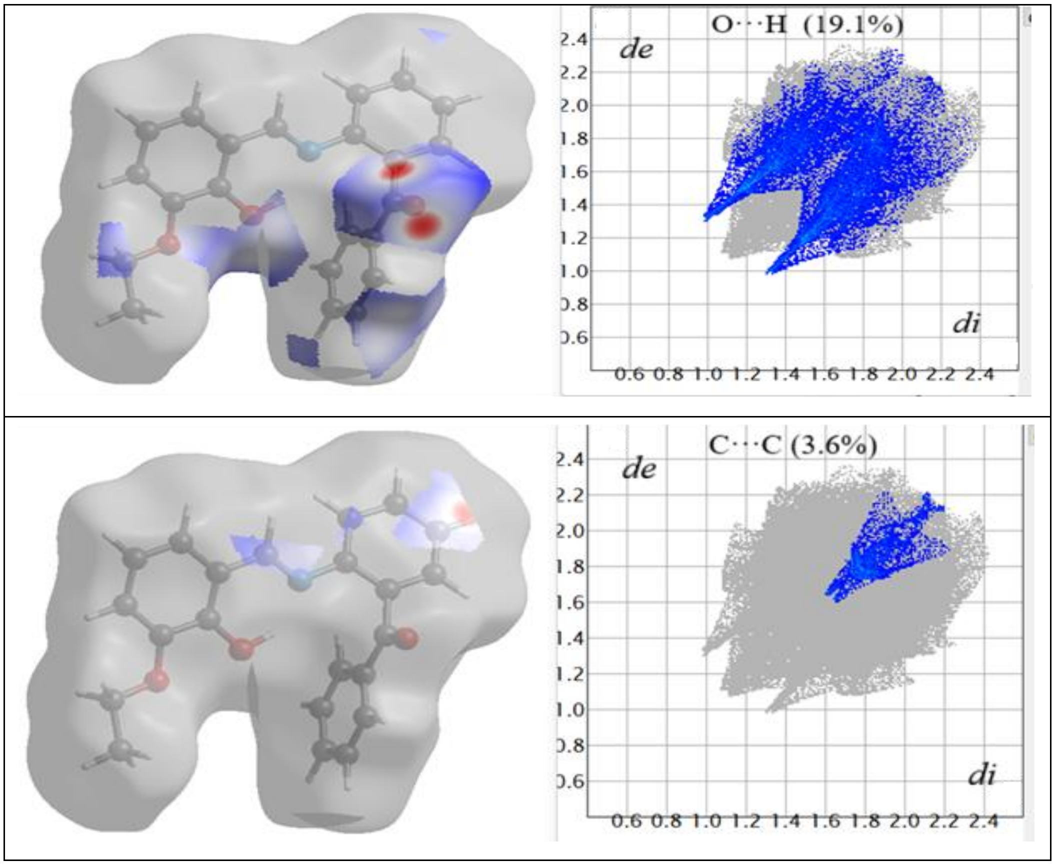

Fig. S12b. Breakdown of all possible contacts to Hirshfeld surfaces for HL2.

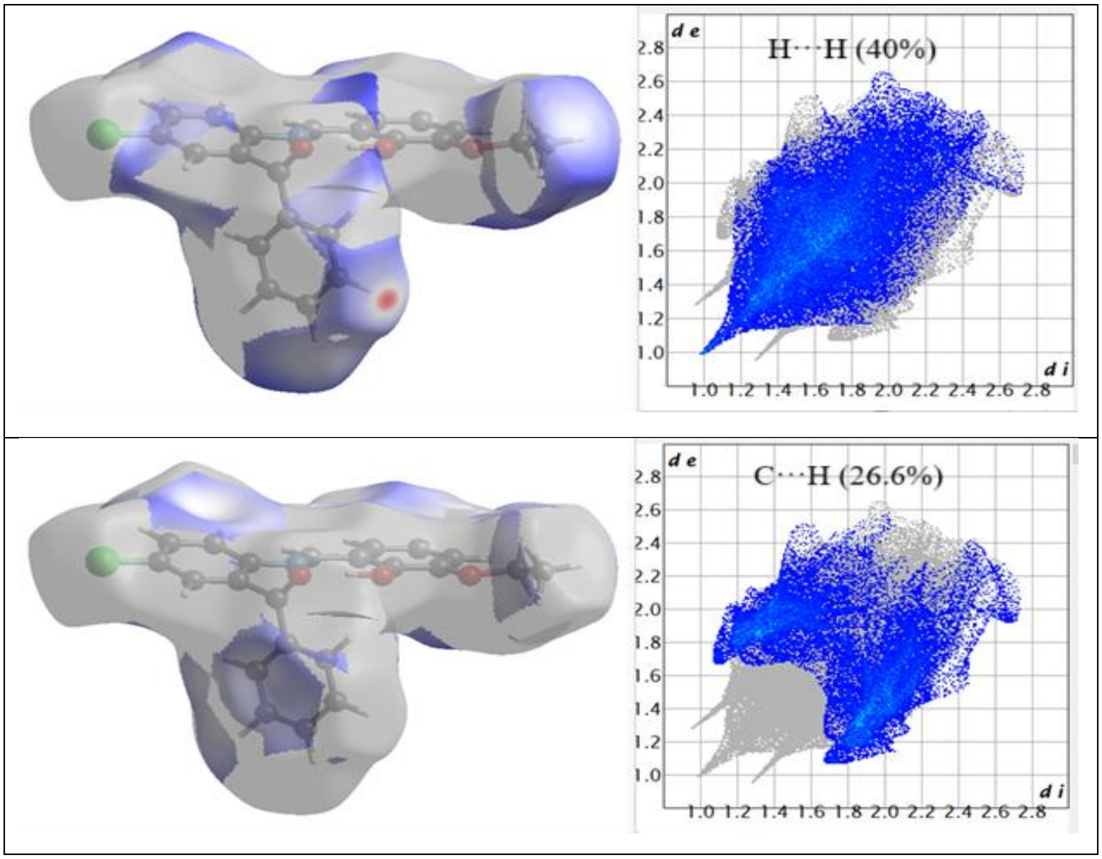

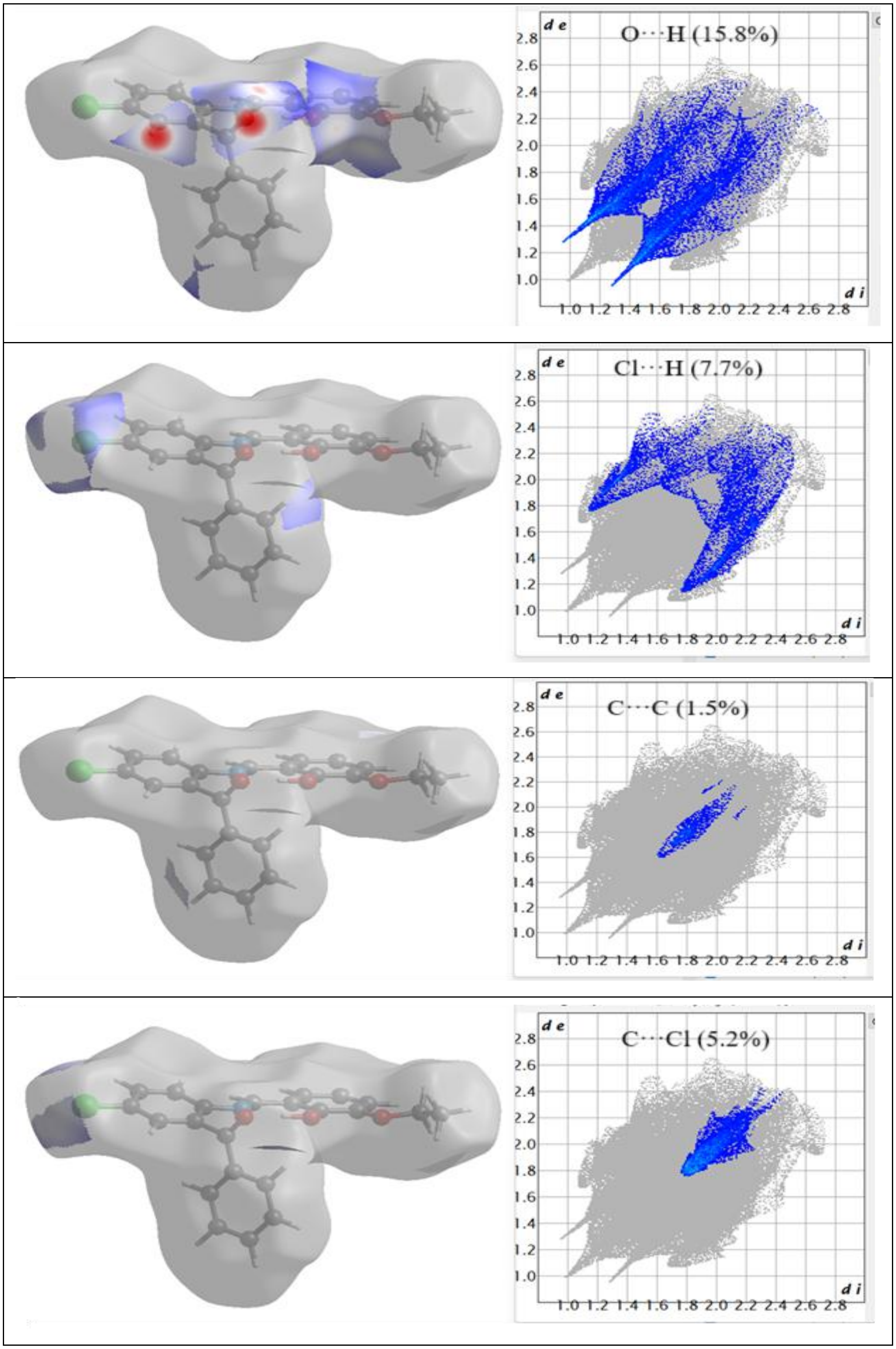
O···H (15.8%)
Cl···H (7.7%)
C···C (1.5%)
C···Cl (5.2%)
d e
d i

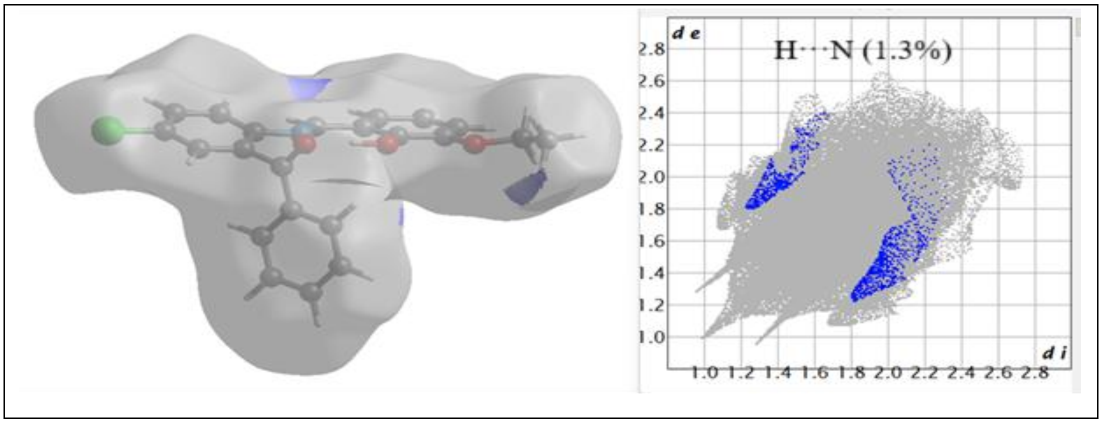

Fig. S12c. Breakdown of all possible contacts to Hirshfeld surfaces for HL3.

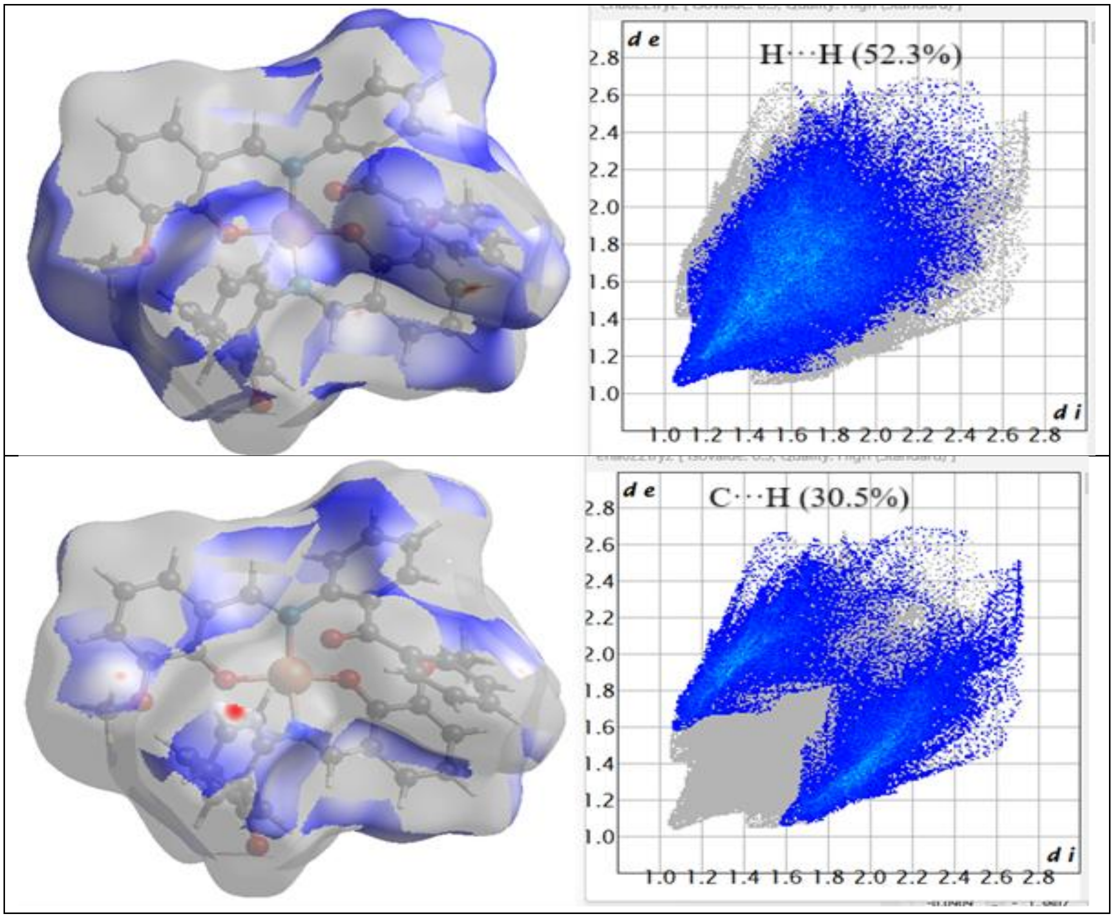

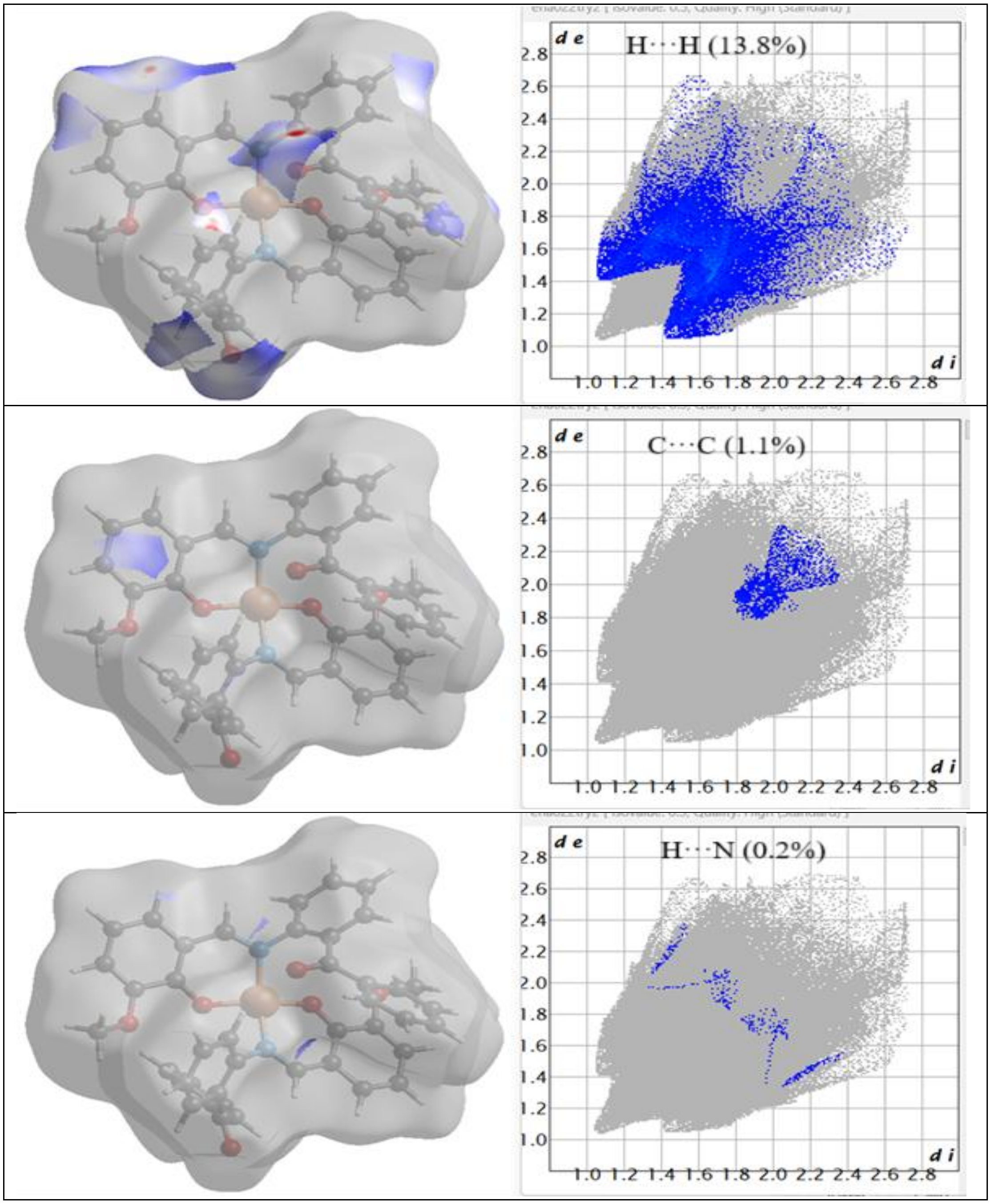

Fig. S13a. Breakdown of all possible contacts to Hirshfeld surfaces for complex **1**.

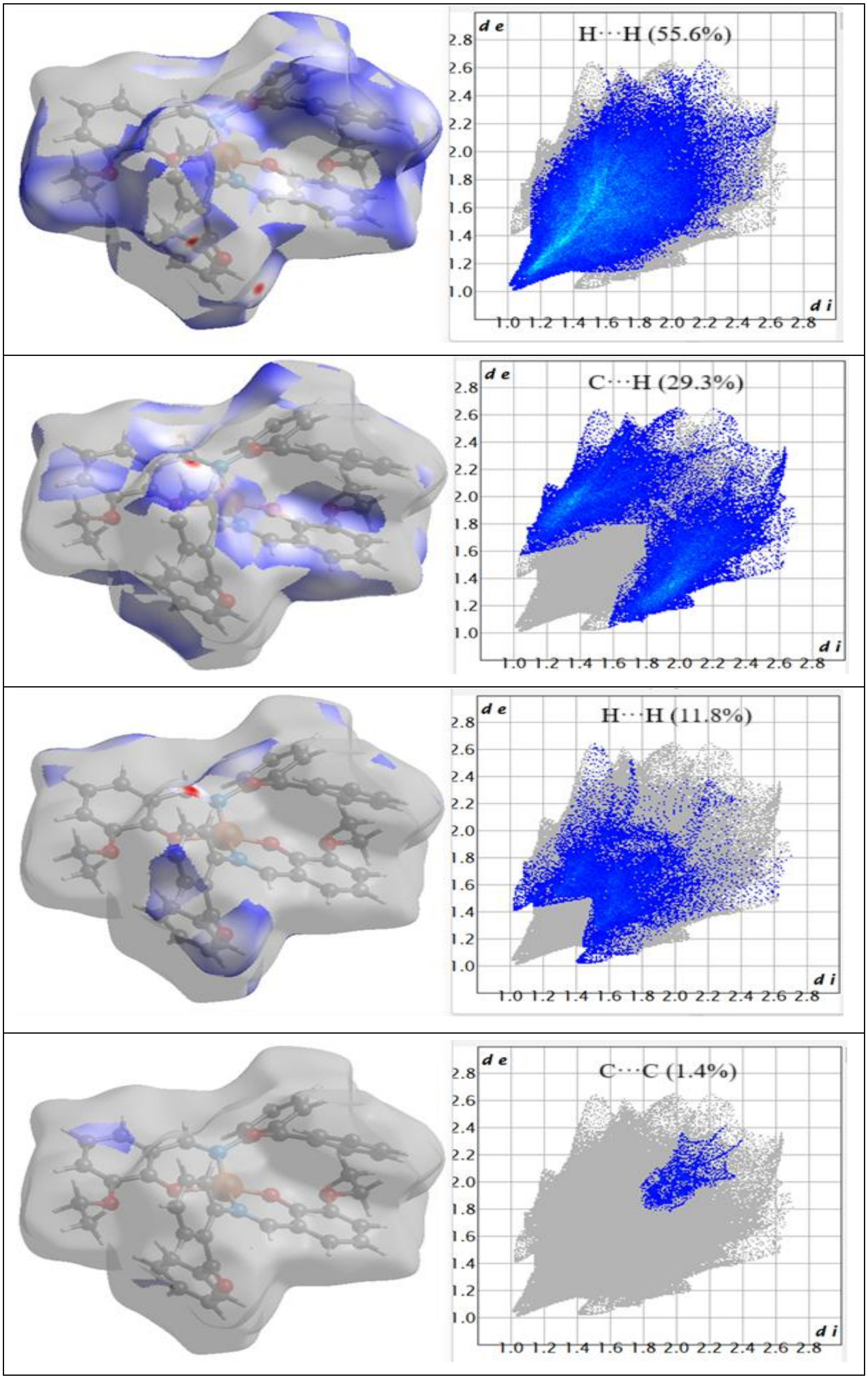

Fig. S13b. Breakdown of all possible contacts to Hirshfeld surfaces for complex **2**.

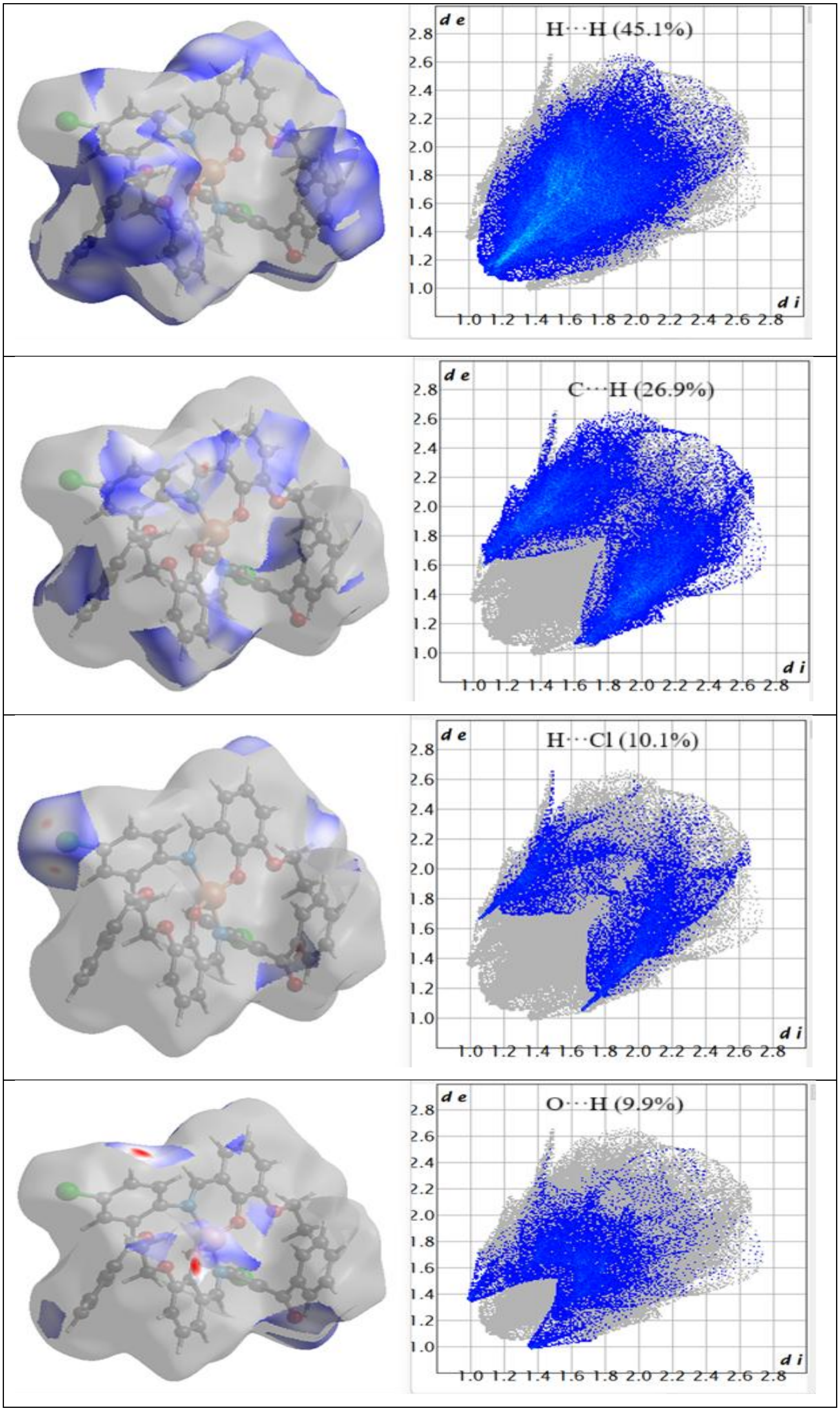

H···H (45.1%)
C···H (26.9%)
H···Cl (10.1%)
O···H (9.9%)
d e
d i
1.0 1.2 1.4 1.6 1.8 2.0 2.2 2.4 2.6 2.8

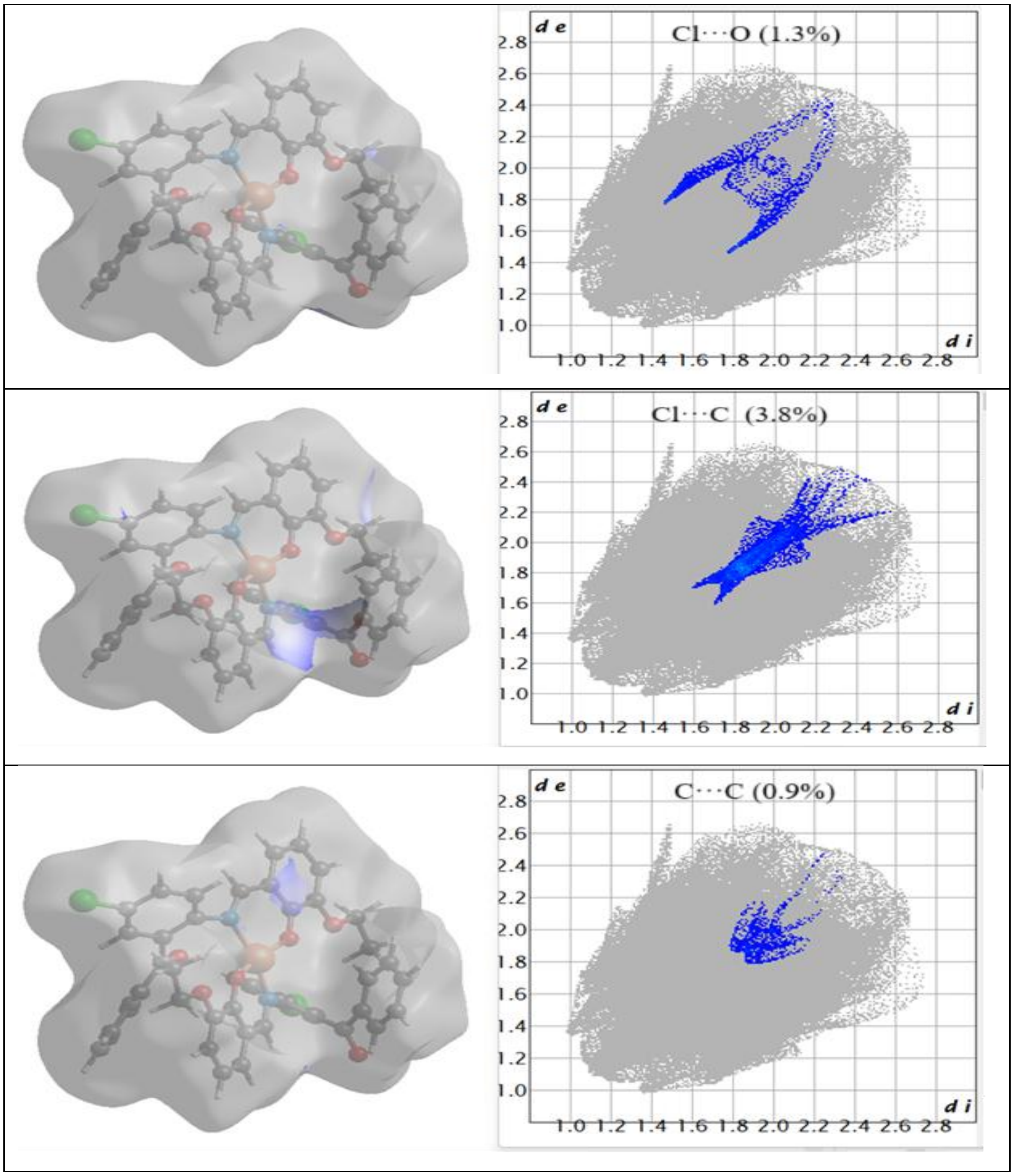

Fig. S13c. Breakdown of all possible contacts to Hirshfeld surfaces for complex **3**.

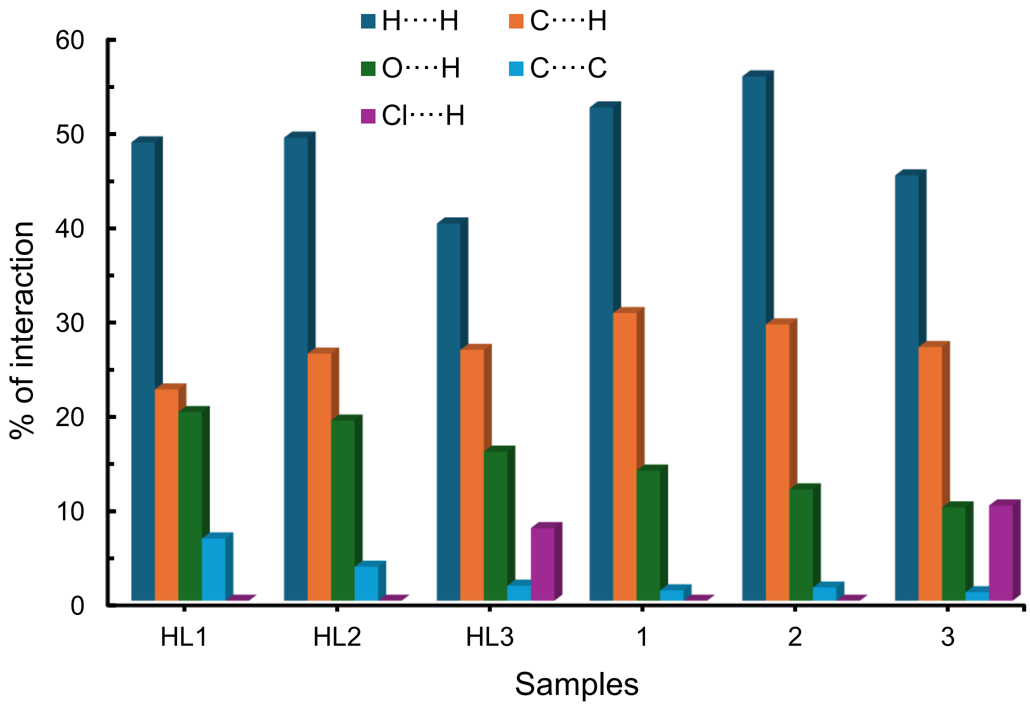

Fig. S14. Plot of relative contributions from all possible contacts to Hirshfeld surfaces for the ligands (HL1, HL2 and HL3) and complexes (**1**, **2** and **3**).

Section 7: Differential scanning calorimetry (DSC)

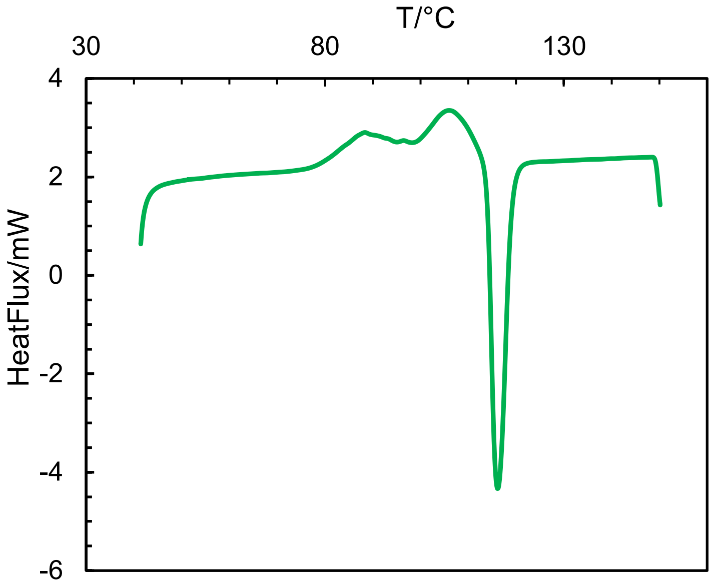

(HL3)

`

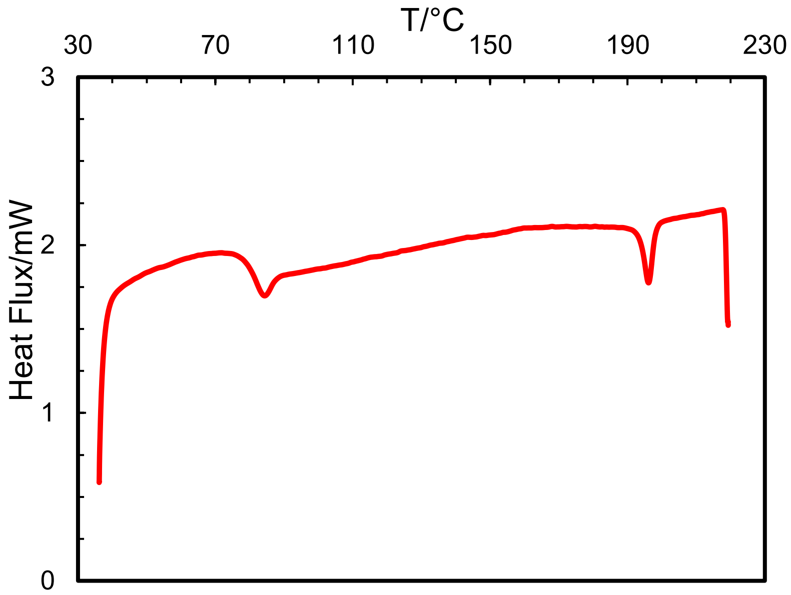

(1)

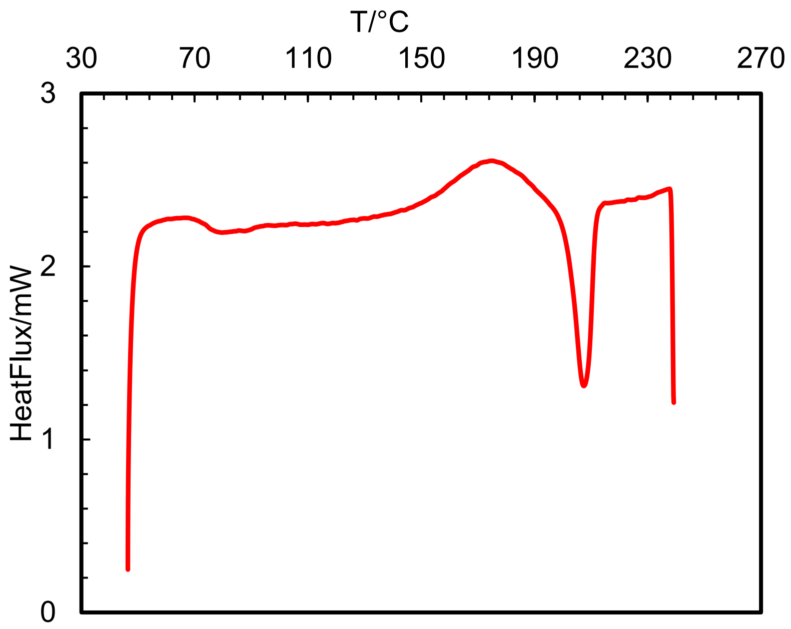

(2)

Fig. S15. Repeated heating curves in the second run of differential scanning calorimetry for the same probe (disc) for the Schiff base (HL3) and complexes (**1** and **2**).

Section 8: Stability assessment of Cu complex 2 after the DPPH assay

To evaluate the stability of the Cu complex during the DPPH assay, the sample was recovered, purified and dried, and characterized by UV-vis. and FT-IR spectroscopy. After incubation, the solvent was removed under reduced pressure at ca. 40 °C. The solid sample was then washed successively with n-hexane and diethyl ether to remove residual DPPH and associated byproducts. The recovered Cu complex showed the identical UV-vis. (Fig. S16) and FT-IR spectra (Fig. S17), indicating that the coordination environment and structural integrity of the complex were fully preserved during the treatment.

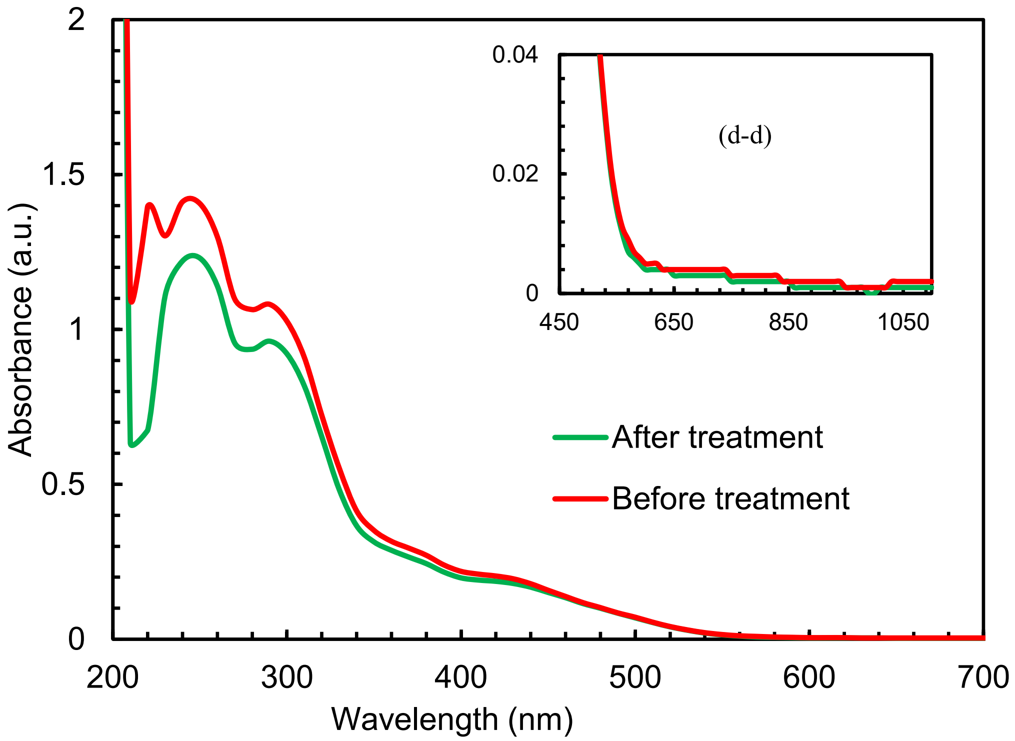

Fig. S16. UV-vis. spectra of Cu complex (**2**) before and after DPPH assay treatment in methanol at room temperature.

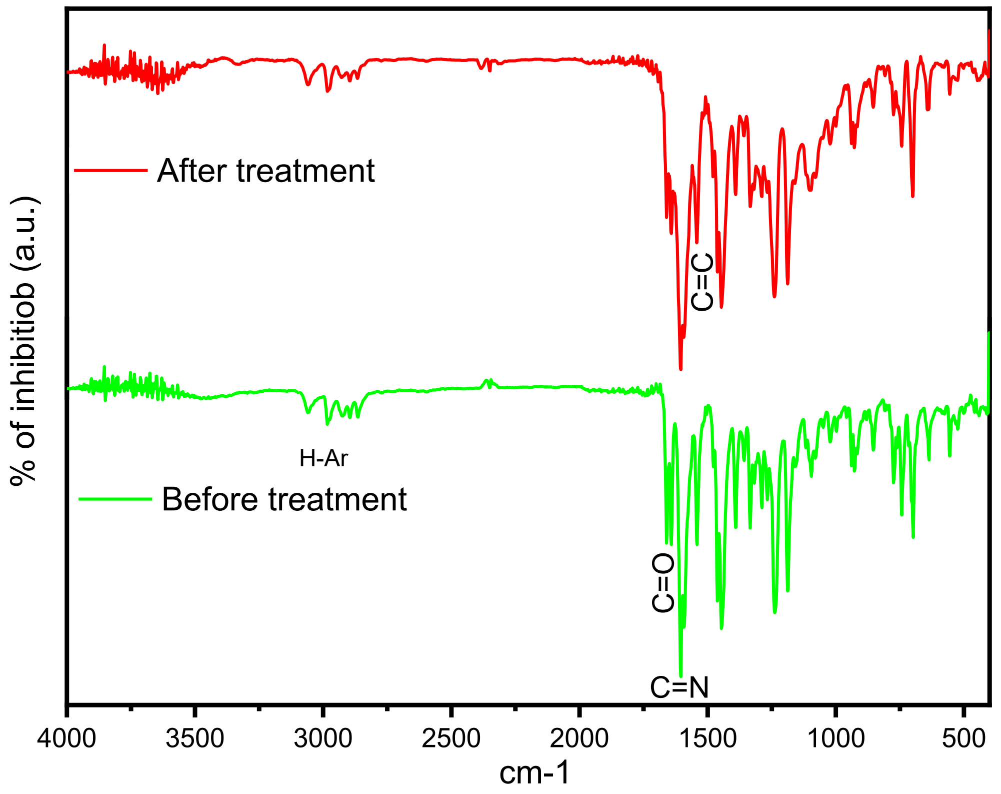

Fig. S17. FT-IR spectra of Cu complex (**2**) before and after DPPH assay treatment in methanol at room temperature.

Section 9: Cyclovoltammetry data

Table S1. Cyclic voltammetry data for complexes (ca. 0.5 mM) in DMF at 25 °C; Electrolyte: TBAP (ca. 0.1 M); Scan rates from 0.05 to 0.30 V/s.

Samples	Scan rate [V/s]	Ea1/[V]	Ia1/[μA]	Ec1/[V]	Ic1/[μA]	Ea2/[V]	Ia2/[μA]	Ia1/Ic1	ΔE1 (Ea1-Ec1)/[V]
**1**	0.05	+0.202	-1.75	-1.002	3.87	-0.738	0.02	-0.45	1.204
	0.10	+0.202	-3.56	-1.002	6.99	-0.738	-0.71	-0.51	1.204
	0.15	+0.202	-4.56	-1.002	9.33	-0.738	-0.83	-0.49	1.204
	0.20	+0.202	-5.80	-1.002	11.99	-0.738	-1.86	-0.48	1.204
	0.30	+0.202	-7.22	-1.002	14.6	-0.738	-2.23	-0.49	1.204
**2**	0.05	+0.209	-17.23	-0.7425	3.19	-0.472	-1.65	-5.40	0.9515
	0.10	+0.209	-23.24	-0.7425	9.92	-0.472	-4.77	-2.34	0.9515
	0.15	+0.209	-25.47	-0.7425	15.60	-0.472	-7.29	-1.63	0.9515
	0.20	+0.209	-27.48	-0.7425	19.21	-0.472	-9.21	-1.43	0.9515
	0.30	+0.209	-30.82	-0.7425	24.78	-0.472	-12.27	-1.24	0.9515
**3**	0.05	+0.262	-6.48	-0.750	2.46	-0.316	-0.91	-2.63	1.012
	0.10	+0.262	-7.89	-0.750	4.90	-0.316	-2.31	-1.61	1.012
	0.15	+0.262	-9.02	-0.750	6.54	-0.316	-3.79	-1.38	1.012
	0.20	+0.262	-10.04	-0.750	7.68	-0.316	-5.02	-1.31	1.012
	0.30	+0.262	-10.63	-0.750	9.45	-0.316	-6.51	-1.12	1.012

Section 10: IC_{50} values

Table S2. In vitro IC_{50} values of Schiff base ligands (HL1 - HL3) and their copper(II) complexes (**1 - 3**).

Compounds	IC_{50} values (μg/mL)
HL1	193.05
HL2	204.59
HL3	214.77
**1**	290.47
**2**	283.08
**3**	282.09

Section 11: Cartesian coordinates of the optimized geometries

Table S3. Cartesian coordinates (Å) of the optimized geometry of HL1 calculated at B3LYP/6-31G(d) level of theory.

Center	Atom	Atomic No.	X (Å)	Y (Å)	Z (Å)
1	O	8	-1.443790	0.572127	0.340663
2	H	1	-0.483408	0.255986	0.316087
3	N	7	0.545098	-1.107851	0.112057
4	O	8	2.915580	0.876974	-2.214139
5	C	6	2.355843	-2.585024	0.894225
6	H	1	1.620787	-3.238892	1.351546
7	C	6	-3.661431	-0.250935	0.016054
8	O	8	-4.064124	1.049252	0.273272
9	C	6	2.883541	-0.525331	-0.298173
10	C	6	0.999760	2.942233	1.669857
11	H	1	0.892913	2.964788	2.749335
12	C	6	-1.757936	-1.777003	-0.185627
13	C	6	1.923056	-1.412834	0.242744
14	C	6	1.790954	1.829286	-0.342147
15	C	6	-0.336867	-2.038868	-0.131448
16	H	1	-0.021527	-3.071379	-0.319062
17	C	6	-2.656314	-2.833388	-0.477858
18	H	1	-2.256857	-3.825373	-0.666854
19	C	6	2.521983	0.735771	-1.034208
20	C	6	4.242082	-0.874439	-0.239777
21	H	1	4.965778	-0.213333	-0.704164
22	C	6	1.649627	1.867827	1.057544
23	H	1	2.053623	1.063641	1.662565
24	C	6	-2.261729	-0.474115	0.061098
25	C	6	3.714442	-2.892028	0.981273
26	H	1	4.030364	-3.792883	1.497476
27	C	6	-4.522442	-1.306788	-0.272124
28	H	1	-5.591975	-1.140075	-0.308988
29	C	6	0.624123	3.955923	-0.502536
30	H	1	0.220169	4.762264	-1.105911
31	C	6	4.663382	-2.040665	0.403241
32	H	1	5.719936	-2.280265	0.456359
33	C	6	-5.481183	1.353644	0.231914
34	H	1	-5.903507	1.156011	-0.760949
35	H	1	-5.547640	2.417704	0.454717
36	H	1	-6.035226	0.782027	0.986676
37	C	6	0.481796	3.983927	0.891535
38	H	1	-0.033041	4.812069	1.368098
39	C	6	1.279768	2.889206	-1.115519
40	H	1	1.408222	2.851409	-2.191293
41	C	6	-4.020038	-2.601164	-0.520749

42	H	1	-4.710128	-3.406979	-0.744304

Table S4. Cartesian coordinates (Å) of the optimized geometry of HL1 calculated at the B3LYP/LANL2DZ level of theory.

Center	Atom	Atomic No.	X (Å)	Y (Å)	Z (Å)
1	O	8	-1.425014	0.549391	0.378796
2	H	1	-0.462524	0.211792	0.339222
3	N	7	0.534029	-1.118019	0.114681
4	O	8	2.922778	0.851062	-2.238376
5	C	6	2.348702	-2.595526	0.914052
6	H	1	1.610203	-3.247629	1.373614
7	C	6	-3.672039	-0.241104	0.033331
8	O	8	-4.066744	1.060248	0.322008
9	C	6	2.886659	-0.536856	-0.296646
10	C	6	1.025404	2.968992	1.657845
11	H	1	0.923600	2.999669	2.739550
12	C	6	-1.773154	-1.789269	-0.203819
13	C	6	1.918132	-1.422321	0.245780
14	C	6	1.799723	1.832138	-0.361597
15	C	6	-0.345724	-2.055818	-0.152744
16	H	1	-0.026703	-3.083399	-0.365995
17	C	6	-2.680514	-2.837249	-0.524377
18	H	1	-2.289760	-3.830122	-0.737345
19	C	6	2.529091	0.724130	-1.049790
20	C	6	4.252206	-0.881329	-0.221493
21	H	1	4.980266	-0.222488	-0.686480
22	C	6	1.667161	1.880071	1.045118
23	H	1	2.071217	1.077654	1.656320
24	C	6	-2.266016	-0.479745	0.074564
25	C	6	3.714442	-2.901856	1.017321
26	H	1	4.027870	-3.799457	1.544762
27	C	6	-4.544981	-1.292052	-0.284758
28	H	1	-5.615702	-1.117372	-0.320298
29	C	6	0.633033	3.971886	-0.532406
30	H	1	0.225001	4.776122	-1.139444
31	C	6	4.673383	-2.048301	0.438354
32	H	1	5.731661	-2.285814	0.503486
33	C	6	-5.486464	1.377017	0.281289
34	H	1	-5.905619	1.206670	-0.720740
35	H	1	-5.548018	2.438024	0.530032
36	H	1	-6.049116	0.790119	1.021515
37	C	6	0.503266	4.013232	0.871158
38	H	1	-0.005383	4.848968	1.345940
39	C	6	1.282595	2.891103	-1.145123
40	H	1	1.400994	2.844020	-2.223773

41	C	6	-4.049496	-2.590467	-0.564805
42	H	1	-4.746801	-3.386285	-0.810084

Table S5. Cartesian coordinates (Å) of the optimized geometry of HL2 calculated at the B3LYP/6-31G(d) level of theory.

Center	Atom	Atomic No.	X (Å)	Y (Å)	Z (Å)
1	O	8	1.253510	0.275842	-0.298578
2	H	1	0.259482	0.091189	-0.291281
3	O	8	3.913545	0.394938	-0.182926
4	O	8	-3.067940	1.122144	2.209751
5	N	7	-0.947118	-1.123294	-0.120801
6	C	6	-1.802485	1.949136	0.368479
7	C	6	-2.351152	-1.239436	-0.275270
8	C	6	-3.193257	-0.241193	0.269879
9	C	6	1.918841	-0.874243	-0.019537
10	C	6	-0.202118	-2.167276	0.122606
11	H	1	-0.656576	-3.150051	0.291270
12	C	6	1.240026	-2.099864	0.202021
13	C	6	-1.629687	1.985946	-1.027675
14	H	1	-2.119571	1.246292	-1.651259
15	C	6	-2.679281	0.949044	1.032383
16	C	6	-1.178205	2.927431	1.165887
17	H	1	-1.332358	2.893496	2.238387
18	C	6	-0.838314	2.977437	-1.612903
19	H	1	-0.708012	2.998910	-2.689820
20	C	6	-4.585153	-0.405492	0.188415
21	H	1	-5.221196	0.338095	0.656377
22	C	6	3.335179	-0.840643	0.051129
23	C	6	-2.926333	-2.332138	-0.954130
24	H	1	-2.278163	-3.070516	-1.414251
25	C	6	-0.210356	3.937313	-0.810746
26	H	1	0.412756	4.700414	-1.266224
27	C	6	-0.382829	3.911152	0.580015
28	H	1	0.105806	4.654126	1.201867
29	C	6	1.982709	-3.270620	0.496101
30	H	1	1.450213	-4.201624	0.666387
31	C	6	4.040494	-2.006497	0.341468
32	H	1	5.121712	-1.986384	0.399341
33	C	6	-5.148297	-1.494042	-0.481597
34	H	1	-6.226408	-1.590100	-0.551997
35	C	6	-4.312284	-2.453880	-1.064008
36	H	1	-4.737240	-3.295853	-1.600960
37	C	6	3.364133	-3.223870	0.565279
38	H	1	3.935607	-4.117273	0.790793
39	C	6	5.366532	0.520482	-0.117928

40	H	1	5.826724	-0.148106	-0.858708
41	H	1	5.716141	0.223671	0.880578
42	C	6	5.688824	1.976232	-0.408983
43	H	1	6.771935	2.139153	-0.376447
44	H	1	5.213176	2.627074	0.330294
45	H	1	5.320954	2.257270	-1.400026

Table S6. Cartesian coordinates (Å) of the optimized geometry of HL3 calculated at the B3LYP/6-31G(d) level of theory.

Center	Atom	Atomic No.	X (Å)	Y (Å)	Z (Å)
1	Cl	17	-5.535858	-2.448113	-0.720340
2	O	8	2.353292	0.260368	-0.870939
3	H	1	1.393116	-0.002995	-0.837161
4	O	8	-0.685184	1.268132	-1.392509
5	O	8	4.909393	0.740589	-0.681385
6	N	7	0.243052	-1.134447	-0.091022
7	C	6	-2.914596	-3.101823	-0.319625
8	H	1	-3.249471	-4.133373	-0.292349
9	C	6	-1.614021	1.005405	-0.641139
10	C	6	-2.352322	2.102302	0.064675
11	C	6	-3.408111	-0.745749	-0.580341
12	H	1	-4.134081	0.041490	-0.751344
13	C	6	2.437526	-1.489467	0.810546
14	C	6	1.022699	-1.772915	0.718087
15	H	1	0.635661	-2.553560	1.388339
16	C	6	-3.830399	-2.071695	-0.521675
17	C	6	-1.566595	-2.790145	-0.168327
18	H	1	-0.847019	-3.593625	-0.045048
19	C	6	3.031616	-0.484071	0.005844
20	C	6	-1.109624	-1.461819	-0.221933
21	C	6	-2.051623	-0.426927	-0.447749
22	C	6	4.427910	-0.239401	0.129591
23	C	6	3.233317	-2.225303	1.721225
24	H	1	2.758440	-2.989733	2.331399
25	C	6	5.182341	-0.982644	1.030695
26	H	1	6.246212	-0.797384	1.125946
27	C	6	4.585185	-1.977421	1.829020
28	H	1	5.198015	-2.541769	2.525139
29	C	6	-3.144897	1.882268	1.201932
30	H	1	-3.262328	0.877808	1.595572
31	C	6	-2.185023	3.413557	-0.411186
32	H	1	-1.553281	3.569511	-1.279519
33	C	6	-3.765913	2.953625	1.844346
34	H	1	-4.371048	2.775309	2.728918
35	C	6	-3.607990	4.250300	1.352759

36	H	1	-4.097573	5.083159	1.850614
37	C	6	6.297715	1.056220	-0.619203
38	H	1	6.895380	0.165881	-0.864926
39	H	1	6.564768	1.369044	0.401134
40	C	6	-2.815449	4.478678	0.222988
41	H	1	-2.688702	5.488442	-0.157711
42	C	6	6.554863	2.174219	-1.614579
43	H	1	7.614195	2.453080	-1.602439
44	H	1	5.957791	3.056196	-1.363708
45	H	1	6.288483	1.855702	-2.626960

Table S7. Cartesian coordinates (Å) of the optimized geometry of the Cu complex (**1**) calculated at the B3LYP/6-31G(d) level of theory.

Center	Atom	Atomic No.	X (Å)	Y (Å)	Z (Å)
1	Cu	29	-0.078540	0.636368	-0.252254
2	O	8	-1.871841	1.222007	-0.341491
3	O	8	1.410696	-0.126928	-1.120361
4	O	8	-4.482878	1.454365	-0.405623
5	O	8	1.524239	1.644984	2.144938
6	O	8	-2.887170	-4.239991	1.318438
7	O	8	3.389505	-0.704470	-2.729691
8	N	7	-0.538921	-1.031110	0.713242
9	N	7	0.659139	2.479570	-0.391294
10	C	6	4.356854	2.417492	0.166472
11	H	1	5.075572	2.008720	0.869331
12	C	6	1.717175	-1.384219	-1.217841
13	C	6	1.497101	-3.781857	-0.648958
14	H	1	0.990247	-4.554738	-0.076437
15	C	6	2.995225	2.141050	0.353841
16	C	6	-0.101918	3.541499	-0.385746
17	H	1	0.400263	4.513413	-0.403158
18	C	6	2.508107	-4.107304	-1.519556
19	H	1	2.816702	-5.139467	-1.653318
20	C	6	-2.339708	2.429372	-0.349672
21	C	6	2.552699	1.344157	1.541476
22	C	6	4.799855	3.225167	-0.879199
23	H	1	5.859374	3.434604	-0.993818
24	C	6	2.054793	2.707631	-0.537622
25	C	6	-1.528616	3.604088	-0.350392
26	C	6	-2.824472	-3.105548	0.853914
27	C	6	1.080402	-2.431412	-0.485572
28	C	6	-5.896346	1.540022	-0.396864
29	H	1	-6.274651	2.082124	-1.274594
30	H	1	-6.264885	2.031683	0.513768
31	H	1	-6.258018	0.510610	-0.424904

32	C	6	0.040321	-2.174165	0.460666
33	H	1	-0.280063	-3.042202	1.042437
34	C	6	-2.129176	4.894648	-0.336218
35	H	1	-1.482997	5.769386	-0.328263
36	C	6	3.385605	0.192095	2.010149
37	C	6	2.513410	3.483228	-1.609572
38	H	1	1.790688	3.859496	-2.327285
39	C	6	2.793832	-1.759802	-2.097300
40	C	6	-2.594242	-1.950290	1.803812
41	C	6	3.871595	3.748888	-1.777003
42	H	1	4.199491	4.360347	-2.613354
43	C	6	-3.768276	2.617609	-0.369057
44	C	6	-3.059679	-2.861147	-0.599679
45	C	6	-3.559075	-1.412881	-2.474714
46	H	1	-3.742051	-0.413613	-2.858894
47	C	6	-3.398855	-0.853685	3.827323
48	H	1	-4.112411	-0.805960	4.645167
49	C	6	4.888395	-1.669650	1.616288
50	H	1	5.485497	-2.264698	0.931162
51	C	6	-3.494637	5.036329	-0.335124
52	H	1	-3.952448	6.020529	-0.322359
53	C	6	-3.296594	-1.578413	-1.114069
54	H	1	-3.280525	-0.704936	-0.472528
55	C	6	-1.554080	-0.999186	1.714690
56	C	6	-3.486075	-1.873238	2.885799
57	H	1	-4.260605	-2.630086	2.965487
58	C	6	-2.383943	0.099742	3.714420
59	H	1	-2.294522	0.896558	4.447730
60	C	6	-4.318119	3.885816	-0.356520
61	H	1	-5.395472	4.011976	-0.363353
62	C	6	4.415514	-0.988197	-3.661671
63	H	1	4.052026	-1.619511	-4.484317
64	H	1	5.273910	-1.484013	-3.186241
65	H	1	4.733914	-0.022984	-4.060305
66	C	6	4.184012	-0.564825	1.137732
67	H	1	4.222731	-0.308846	0.084206
68	C	6	4.814410	-2.017483	2.965998
69	H	1	5.369812	-2.874824	3.337263
70	C	6	-3.098162	-3.972579	-1.459578
71	H	1	-2.926937	-4.958483	-1.039457
72	C	6	3.161776	-3.084982	-2.246254
73	H	1	3.965206	-3.353672	-2.923927
74	C	6	-1.468081	0.023416	2.671082
75	H	1	-0.644034	0.727597	2.609623
76	C	6	-3.583921	-2.518598	-3.326009
77	H	1	-3.783348	-2.385101	-4.386466
78	C	6	3.297346	-0.179967	3.361541

79	H	1	2.658147	0.400412	4.019116
80	C	6	-3.353316	-3.801362	-2.815653
81	H	1	-3.373786	-4.663031	-3.477734
82	C	6	4.017796	-1.269977	3.839373
83	H	1	3.955304	-1.543806	4.889061

Table S8. Cartesian coordinates (Å) of the optimized geometry of the Cu complex (**2**) calculated at the B3LYP/6-31G(d) level of theory.

Center	Atom	Atomic No.	X (Å)	Y (Å)	Z (Å)
1	Cu	29	-0.047522	0.628641	-0.156878
2	O	8	1.344114	1.599841	2.294199
3	O	8	1.496315	-0.150280	-0.911190
4	O	8	3.607054	-0.740605	-2.337755
5	O	8	-1.830108	1.222088	-0.352153
6	O	8	-3.052030	-4.231504	1.083253
7	C	6	4.118860	-0.566371	1.547012
8	H	1	4.242018	-0.319860	0.497745
9	C	6	-0.051070	3.531456	-0.364359
10	H	1	0.455936	4.500941	-0.374462
11	N	7	-0.597399	-1.036634	0.765429
12	C	6	2.654862	3.461509	-1.351886
13	H	1	1.995738	3.835427	-2.129529
14	C	6	1.137351	-2.443136	-0.251830
15	C	6	2.431537	1.320968	1.791562
16	C	6	2.969953	2.124640	0.648998
17	N	7	0.703778	2.467540	-0.284312
18	C	6	4.341943	2.404918	0.577831
19	H	1	4.998819	1.999521	1.340404
20	C	6	-2.768081	0.083580	3.544530
21	H	1	-2.760055	0.873898	4.290167
22	C	6	-2.888643	-3.102174	0.630668
23	C	6	-1.738009	0.008569	2.614458
24	H	1	-0.908869	0.708430	2.650422
25	C	6	3.239257	0.186587	2.341142
26	C	6	-3.037778	-1.584252	-1.399118
27	H	1	-3.097876	-0.704501	-0.768354
28	C	6	5.193485	0.302954	-3.761315
29	H	1	5.465503	0.998647	-2.961411
30	H	1	4.400999	0.763451	-4.359225
31	H	1	6.067665	0.142593	-4.401994
32	C	6	0.008794	-2.179387	0.583966
33	H	1	-0.366671	-3.042978	1.139133
34	C	6	4.796389	-1.655936	2.094283
35	H	1	5.455827	-2.248957	1.467046
36	C	6	1.582289	-3.791523	-0.344020

37	H	1	1.036282	-4.557393	0.201315
38	C	6	-3.796030	-0.863302	3.529757
39	H	1	-4.598786	-0.817402	4.260400
40	C	6	-2.069448	4.893268	-0.482412
41	H	1	-1.419427	5.765207	-0.484711
42	C	6	-3.778859	-1.875555	2.576676
43	H	1	-4.558228	-2.631382	2.561906
44	O	8	-4.440038	1.462475	-0.449821
45	C	6	2.669591	-4.123487	-1.114101
46	H	1	3.001744	-5.154015	-1.193103
47	C	6	4.021722	3.729972	-1.403793
48	H	1	4.418117	4.340694	-2.210486
49	C	6	-2.766760	-1.950916	1.604819
50	C	6	-3.099913	-1.431550	-2.785244
51	H	1	-3.208152	-0.435238	-3.204566
52	C	6	-6.403341	0.131940	-0.287145
53	H	1	-6.092094	-0.476717	-1.141550
54	H	1	-7.498182	0.150500	-0.250811
55	H	1	-6.029527	-0.342102	0.625621
56	C	6	-3.433048	5.039008	-0.540612
57	H	1	-3.885924	6.024365	-0.590756
58	C	6	-4.262130	3.892182	-0.533153
59	H	1	-5.337739	4.023055	-0.579528
60	C	6	4.871361	3.211949	-0.427630
61	H	1	5.935764	3.426907	-0.449446
62	C	6	3.043491	-0.173991	3.684282
63	H	1	2.343043	0.402249	4.280239
64	C	6	-3.720802	2.621211	-0.465704
65	C	6	4.615658	-1.991299	3.437065
66	H	1	5.150758	-2.836100	3.862855
67	C	6	-3.022767	-2.546835	-3.621219
68	H	1	-3.066021	-2.422310	-4.700406
69	C	6	3.370741	-3.110622	-1.809375
70	H	1	4.233321	-3.384992	-2.406993
71	C	6	-2.889992	-3.826480	-3.070922
72	H	1	-2.830416	-4.695714	-3.720591
73	C	6	-2.835567	-3.985253	-1.690523
74	H	1	-2.745258	-4.968831	-1.240984
75	C	6	3.737954	-1.247683	4.232548
76	H	1	3.592196	-1.511932	5.276417
77	C	6	-5.861770	1.545941	-0.408460
78	H	1	-6.234852	2.034297	-1.321271
79	H	1	-6.172007	2.159441	0.450151
80	C	6	1.823603	-1.405420	-0.951955
81	C	6	-1.718572	-1.006223	1.646017
82	C	6	2.974601	-1.787370	-1.729462
83	C	6	2.106875	2.689396	-0.319626

84	C	6	-2.901667	-2.865196	-0.844126
85	C	6	4.720472	-1.018127	-3.179107
86	H	1	4.423512	-1.715311	-3.976638
87	H	1	5.521917	-1.499481	-2.597789
88	C	6	-1.476261	3.600512	-0.419116
89	C	6	-2.293292	2.430286	-0.407707

Table S9. Cartesian coordinates (Å) of the optimized geometry of the Cu complex (**3**) calculated at the B3LYP/6-31G(d) level of theory.

Center	Atom	Atomic No.	X (Å)	Y (Å)	Z (Å)
1	Cu	29	0.022204	0.541346	-0.315512
2	Cl	17	-5.707211	-0.188723	3.862740
3	Cl	17	7.003735	2.348091	0.353286
4	O	8	3.673025	-1.305280	-2.117180
5	O	8	1.508383	-0.442596	-0.926525
6	O	8	1.218990	1.226097	2.362401
7	O	8	-1.613353	1.417489	-0.665384
8	O	8	-4.124035	2.081050	-1.053422
9	N	7	-0.872846	-1.045916	0.459503
10	C	6	-3.247236	-2.419909	-1.487615
11	N	7	1.057717	2.237957	-0.248209
12	C	6	4.492110	1.557278	1.065749
13	H	1	4.980542	1.034072	1.879333
14	C	6	0.493602	3.411891	-0.366664
15	H	1	1.141778	4.287649	-0.266907
16	C	6	2.294441	0.772637	1.976630
17	C	6	-4.343707	-1.420985	1.848638
18	H	1	-5.229887	-2.031504	1.715845
19	C	6	-0.433562	-2.264633	0.283442
20	H	1	-1.000583	-3.076818	0.745690
21	C	6	0.975577	-4.070815	-0.545647
22	H	1	0.257919	-4.760804	-0.108822
23	C	6	3.666208	-1.361343	1.822916
24	H	1	3.966551	-1.097928	0.814387
25	C	6	-6.282175	1.092194	-1.223118
26	H	1	-6.125898	0.534132	-0.294970
27	H	1	-5.957362	0.466461	-2.059944
28	H	1	-7.353240	1.294371	-1.332513
29	C	6	-3.132445	-1.111807	-1.981261
30	H	1	-3.132489	-0.258149	-1.312603
31	C	6	3.265736	2.925705	-1.014835
32	H	1	2.787330	3.422413	-1.853142
33	C	6	-2.048265	0.104900	2.221905
34	H	1	-1.132496	0.662711	2.391731
35	C	6	-2.560748	5.447902	-0.866522

36	H	1	-2.844059	6.494274	-0.922999
37	C	6	2.086951	-4.540406	-1.200598
38	H	1	2.267112	-5.606623	-1.296293
39	C	6	-3.242015	-1.623938	1.002784
40	C	6	-1.254289	5.082826	-0.660088
41	H	1	-0.479809	5.837996	-0.548252
42	C	6	3.014387	-3.625655	-1.752454
43	H	1	3.892973	-4.010448	-2.258847
44	C	6	5.573133	-0.482046	-3.282577
45	H	1	5.868022	0.120729	-2.418110
46	H	1	4.940446	0.132016	-3.930914
47	H	1	6.475432	-0.761431	-3.837463
48	O	8	-3.787628	-3.820788	0.345419
49	C	6	-3.154324	0.323912	3.032672
50	H	1	-3.124804	1.074370	3.815241
51	C	6	5.256346	2.292238	0.164350
52	C	6	-3.019107	-0.897127	-3.355760
53	H	1	-2.933373	0.119644	-3.728313
54	C	6	-3.554626	4.450145	-1.005040
55	H	1	-4.582716	4.754662	-1.166445
56	C	6	-4.300954	-0.447112	2.837128
57	C	6	-3.015642	-1.977646	-4.239627
58	H	1	-2.922138	-1.805585	-5.308973
59	C	6	-3.255038	-3.503375	-2.383211
60	H	1	-3.359399	-4.506352	-1.982223
61	C	6	2.824262	-2.078655	4.392205
62	H	1	2.500143	-2.357349	5.391079
63	C	6	4.077692	-2.571727	2.380270
64	H	1	4.710291	-3.241023	1.804515
65	C	6	-3.424264	-2.713142	-0.035977
66	C	6	4.652186	2.971288	-0.889738
67	H	1	5.253414	3.522072	-1.605266
68	C	6	2.394338	-0.880352	3.831652
69	H	1	1.726319	-0.217736	4.372438
70	C	6	-3.134232	-3.283489	-3.750760
71	H	1	-3.133117	-4.125063	-4.438285
72	C	6	3.666539	-2.926932	3.665926
73	H	1	3.994823	-3.867403	4.100511
74	C	6	1.647741	-1.731485	-0.993716
75	C	6	0.731879	-2.672952	-0.433346
76	C	6	-2.069583	-0.866978	1.210121
77	C	6	-1.870811	2.686200	-0.722116
78	C	6	-0.884110	3.709441	-0.587442
79	C	6	2.823753	-0.503185	2.548306
80	C	6	-3.233155	3.106693	-0.934039
81	C	6	2.468614	2.230315	-0.096826
82	C	6	3.097417	1.509973	0.945283

83	C	6	-5.507525	2.398103	-1.194636
84	H	1	-5.665800	2.970356	-2.120919
85	H	1	-5.830665	3.026886	-0.352325
86	C	6	2.817108	-2.259979	-1.647947
87	C	6	4.827110	-1.727886	-2.837255
88	H	1	4.524506	-2.335455	-3.702855
89	H	1	5.462346	-2.356004	-2.194447

Section S12 Overlay plots of the X-ray and optimized structures

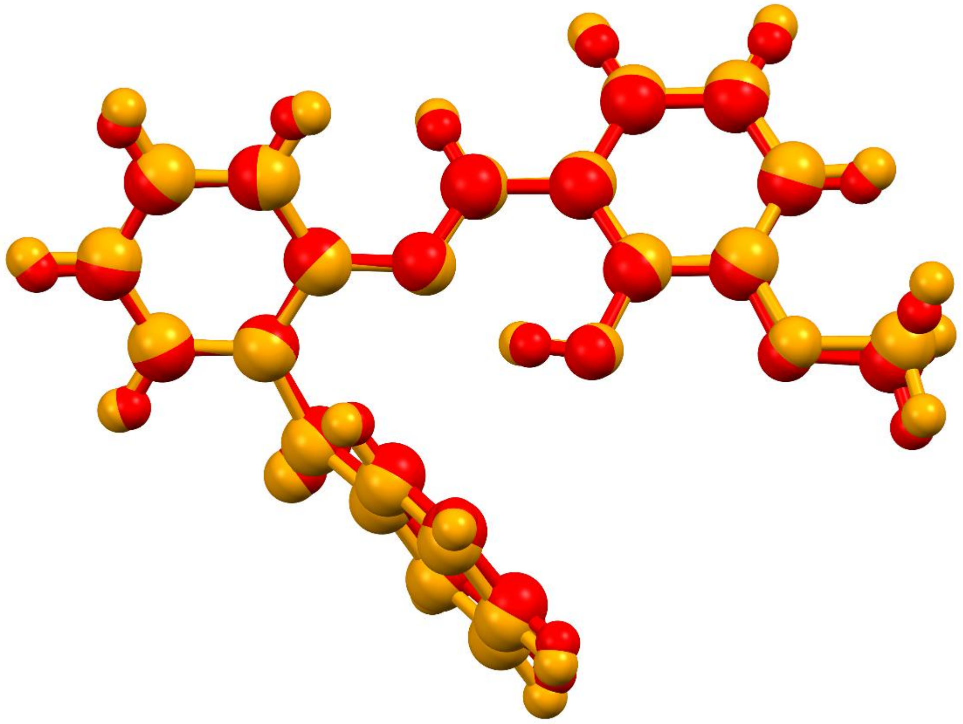

HL1:

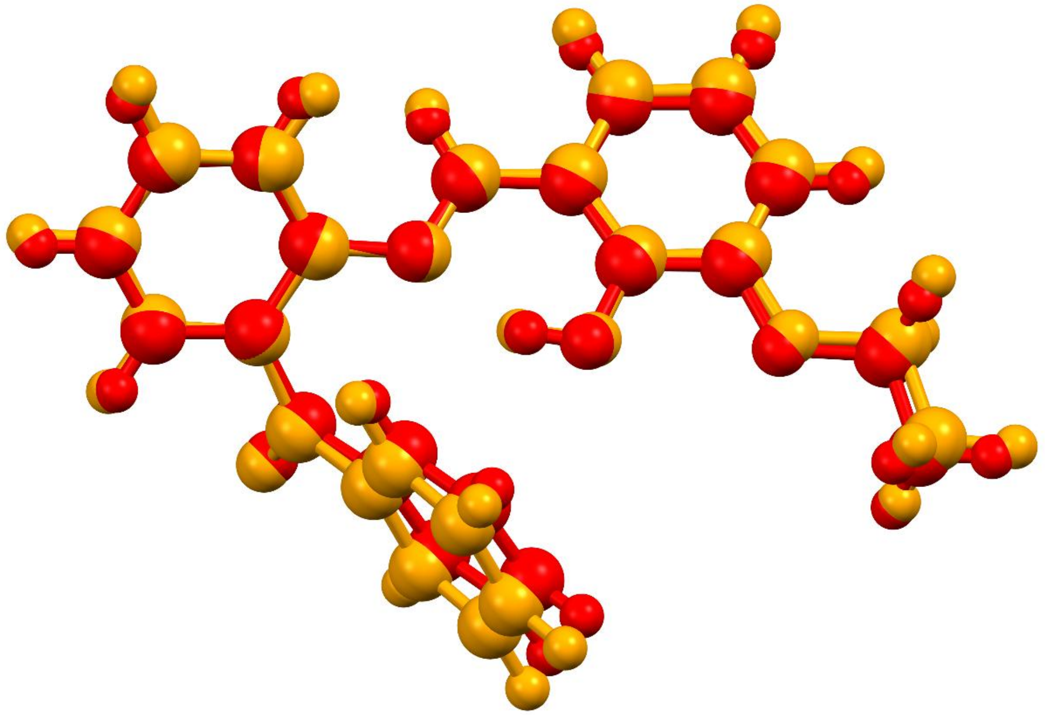

HL2:

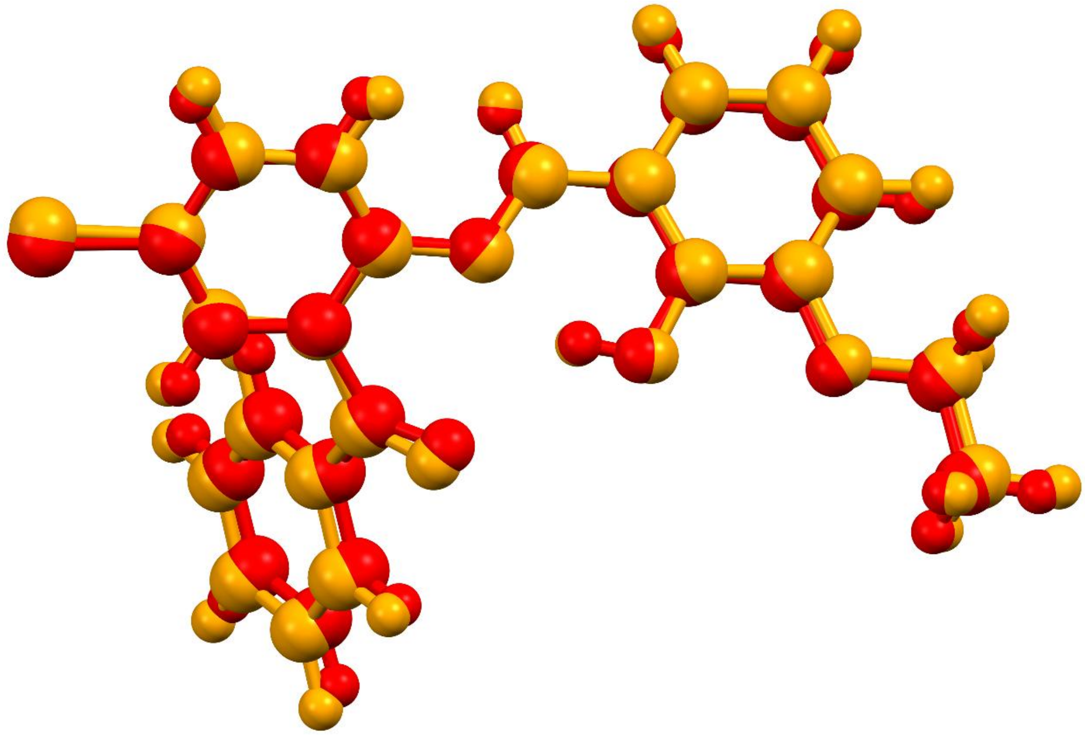

HL3:

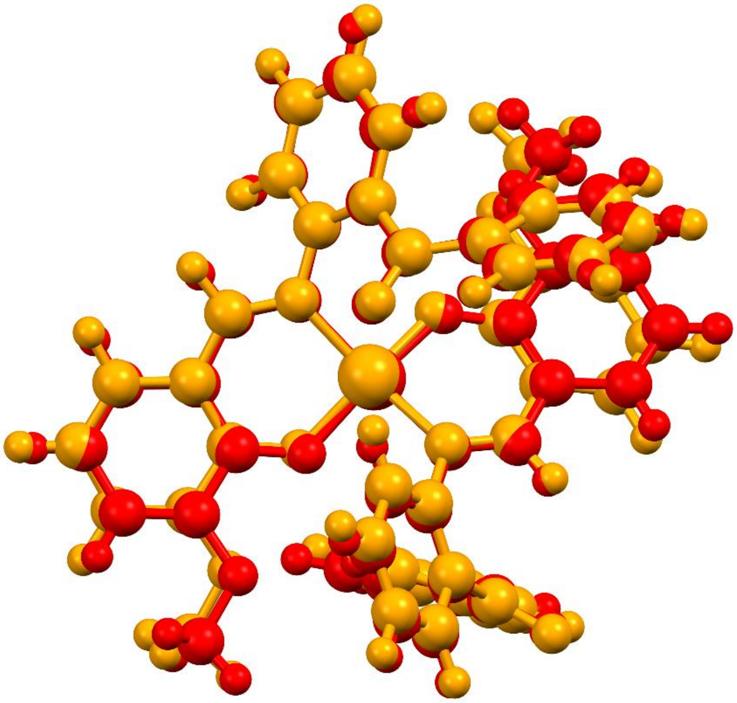

complex **1**:

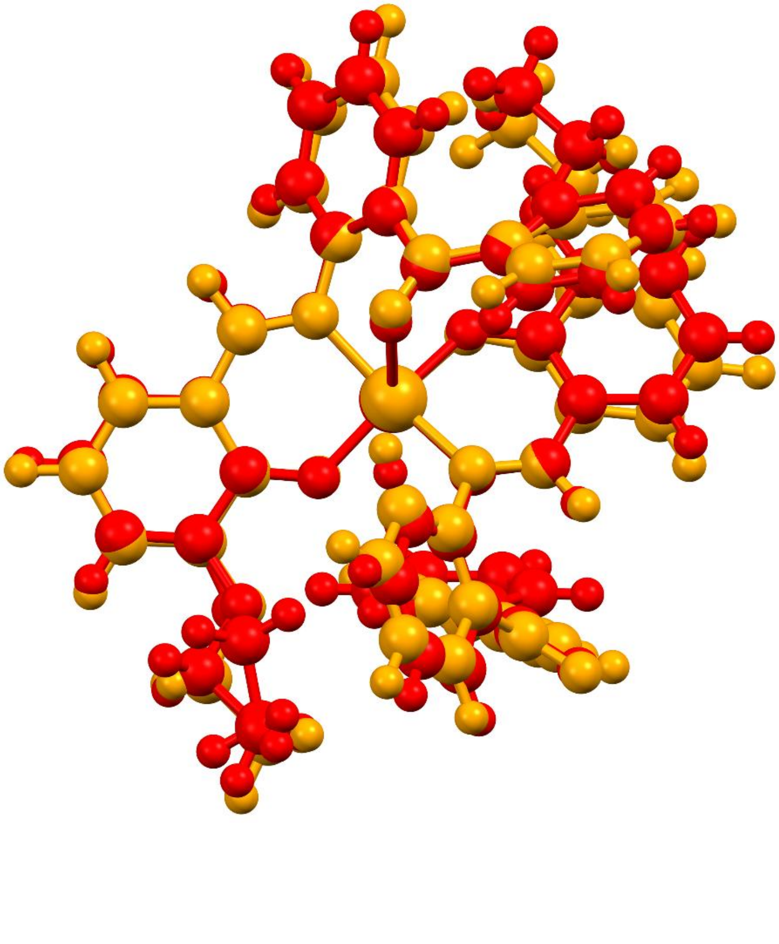

complex **2**:

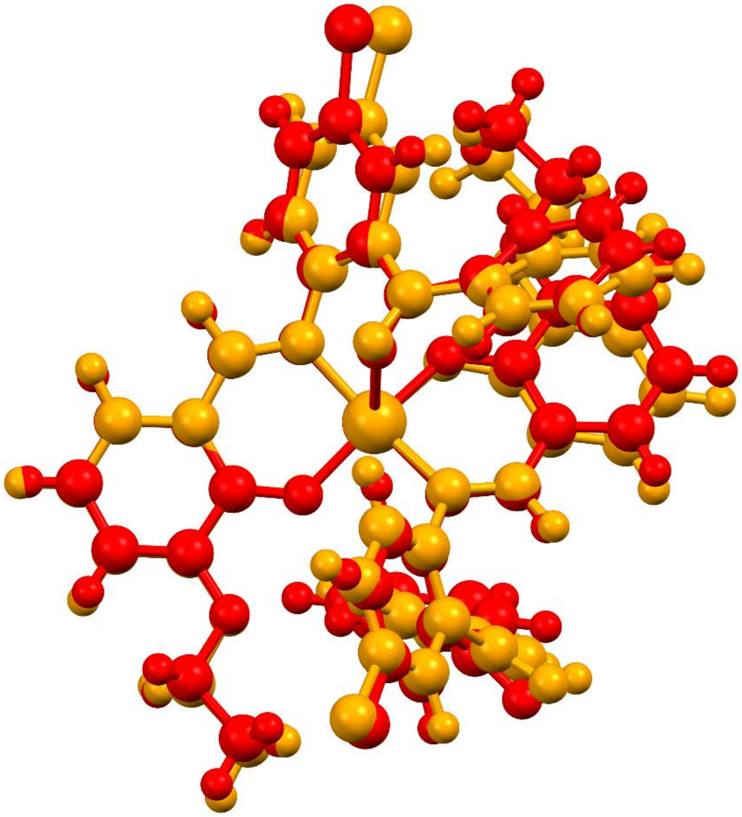

complex **3**:

Fig. S18 Overlay plots of the molecule in the solid-state X-ray structure (in red) and from the gas-phase optimized structures (in orange). The structures were optimized at the B3LYP/6-31G(d) level with the initial geometry for optimization taken from the cif files of the ligands and complexes.
